# Dissemination and Mechanism for the MCR-1 Colistin Resistance

**DOI:** 10.1371/journal.ppat.1005957

**Published:** 2016-11-28

**Authors:** Rongsui Gao, Yongfei Hu, Zhencui Li, Jian Sun, Qingjing Wang, Jingxia Lin, Huiyan Ye, Fei Liu, Swaminath Srinivas, Defeng Li, Baoli Zhu, Ya-Hong Liu, Guo-Bao Tian, Youjun Feng

**Affiliations:** 1 Department of Medical Microbiology and Parasitology, Zhejiang University School of Medicine, Hangzhou, Zhejiang, China; 2 CAS Key Laboratory of Pathogenic Microbiology and Immunology, Institute of Microbiology, Chinese Academy of Sciences, Beijing, China; 3 National Risk Assessment Laboratory for Antimicrobial Resistance of Animal Original Bacteria, South China Agricultural University, Guangzhou, China; 4 Department of Biochemistry, University of Illinois, Urbana, Illinois, United States of America; 5 Institute of Biophysics, Chinese Academy of Sciences, Beijing, China; 6 Zhongshan School of Medicine, Sun Yat-sen University, Guangzhou, China; National Jewish Health, UNITED STATES

## Abstract

Polymyxins are the last line of defense against lethal infections caused by multidrug resistant Gram-negative pathogens. Very recently, the use of polymyxins has been greatly challenged by the emergence of the plasmid-borne mobile colistin resistance gene (*mcr-1*). However, the mechanistic aspects of the MCR-1 colistin resistance are still poorly understood. Here we report the comparative genomics of two new *mcr*-*1*-harbouring plasmids isolated from the human gut microbiota, highlighting the diversity in plasmid transfer of the *mcr*-*1* gene. Further genetic dissection delineated that both the trans-membrane region and a substrate-binding motif are required for the MCR-1-mediated colistin resistance. The soluble form of the membrane protein MCR-1 was successfully prepared and verified. Phylogenetic analyses revealed that MCR-1 is highly homologous to its counterpart PEA lipid A transferase in *Paenibacili*, a known producer of polymyxins. The fact that the plasmid-borne MCR-1 is placed in a subclade neighboring the chromosome-encoded colistin-resistant *Neisseria* LptA (EptA) potentially implies parallel evolutionary paths for the two genes. In conclusion, our finding provids a first glimpse of mechanism for the MCR-1-mediated colistin resistance.

## Author Summary

Colistin is an ultimate line of refuge against fatal infections by multidrug-resistant Gram-negative pathogens. The plasmid-mediated transfer of the mobile colistin resistance gene (*mcr-1*) represents a novel mechanism for antibacterial drug resistance, and also poses new threats to public health. However, the mechanistic aspects of the MCR-1 colistin resistance are not fully understood. Here we report comparative genomics of two new *mcr-1*-harbouring plasmids isolated from the human gut microbiota. Genetic studies determined that both the transmembrane region and a substrate-binding motif are essential for its function. Phylogenetic analyses revealed that MCR-1 is highly homologous to the PEA lipid A transferase in *Paenibacillus*, a known producer of polymyxins. The fact that the plasmid-borne MCR-1 is placed in a subclade neighboring the chromosome-encoded colistin-resistant *Neisseria* LptA potentially implies parallel evolutionary paths for the two genes. Our results reveal mechanistic insights into the MCR-1-mediated colistin resistance.

## Introduction

**Fig 1:**
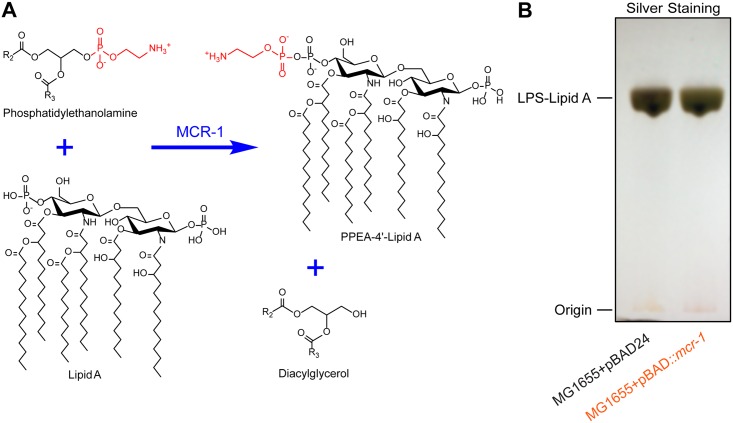
Working model proposed for MCR-1-catalyzed reaction in *E*. *coli*. **A.** The chemical mechanism for MCR-1-mediated colistin resistance MCR-1 catalyzes the reaction of PPEA-4’-lipid A generation from lipid A plus phosphatidylethanolamine in which diacylglycerol is also one end product. In light of its similarity to the *Neisseria* LptA [2], the MCR-1-mediated reaction was given, in which the chemical structures of molecules were generated using the software ChemDraw. **B.** Silver staining analyses for the isolated *E*. *coli* lipopolysaccharide (LPS) containing lipid A (LPS-Lipid A). The bacterial LPS were isolated as described by Wanty *et al*. [2] with appropriate modifications.

The polymyxins (polymyxin E (colistin) and polymyxin B) are a family of cationic polypeptide antibiotics with a lipophilic fatty acyl side chain [1,2]. The initial binding of polymyxins bacterial surface mainly depends on the electrostatic interaction between the positively-charged polymyxin and the negatively-charged phosphate group of lipid A on lipopolysaccharide (LPS) localized on the outer leaflet of the bacterial outer membrane [2]. Following its diffusion from the outer membrane across the periplasm, polymyxin intercalates into the inner membrane and forms pores, which in turn results in bacterial lysis [2]. Although they belong to an old generation of antibiotics, polymyxins represent the last line of defense against lethal infections by gram-negative pathogens with pan-drug resistance [3]. Unfortunately, certain species of the Enterobacteriaceae like *K*. *pneumoniae* [3] have been recently showing an appreciable resistance to colistin. Indeed, colistin resistance (i.e., inefficient binding of polymyxins to the lipid A moiety of lipopolysaccharide) is mainly due to the 4’-phosphoethanolamine (PEA) modification of the lipid A on the LPS [4,5]. This type of chemical modification on the bacterial lipid A can be attributed to either the chromosome-encoded machinery in *K*. *pneumoniae* [6] or the plasmid-transferred mobilized colistin resistance (MCR-1) gene in certain species of Enterobacteriaceae like *E*. *coli* [7]. For the former, two sets of bacterial two-component systems (*pmrAB* [8] plus *phoPQ* [6]) and the regulator *mgrB* [6] are implicated, in which the lipid A of LPS is chemically modified and thereafter exhibits reduced affinity to polymyxin [7]. The latter represents an unique mechanism for bacterial colistin resistance in that the *mcr-1* gene product, annotated as a member of a family of phosphoethanolamine transferases, catalyzes the modification of lipid A moiety on bacterial LPS (Fig 1) [2,7]. To the best of our knowledge, the natural occurrence of the *mcr*-*1* gene has been traced to no less than five species: *E*. *coli* [7,9,10], *Salmonella enteric* [11], *K*. *pneumonia* [7], *Enterobacter aerogenes* [12] and *E*. *cloacae* [12] (of note, it was also experimentally spread/transmitted from *E*. *coli* to *Pseudomonas aeruginosa* by conjugation [7,13].). Also, the range of host reservoirs with potential to carry the *mcr-1*-harbouring enterobacteria extends from poultry/livestock (chickens [11], pigs [7,11,14–16], dogs [17], and cattle [11]) to humans [10], and published data from January-April 2016 suggests that the *mcr*-*1* gene has been disseminated into no less than 18 countries [10]. To a certain degree, the global spread of the *mcr*-*1* gene might be related to a food-chain based dissemination pathway, which was shown by Zhu’s group [11]. Thus, they observed the paralleled existence of *mcr*-*1* in meat/food samples and in the healthy human microbiome [11]. Worryingly, the MCR-1 colistin resistance gene was strikingly shown to coexist with other multiple-drug resistance genes (i.e, carbapenem [18] and extended-spectrum β-lactam [16,19–21]), highlighting the possibility that micro-organisms with pan-drug resistances are emerging [22]. For instance, a variant of the notorious NDM-1 was detected to coexist with MCR-1 in the Enterobacteriaceae (NDM-5 in *K*. *pneumoniae* [23] and NDM-9 in a chicken meat isolate of *E*. *coli* [24]). So far, most of the studies in this field focused on epidemiological investigations, which is in part due to the relatively limited availability of the genomic information. Nevertheless, the mechanism for transfer, origin, and biochemical analysis of the diversified plasmid-borne MCR-1 colistin resistance remains poorly understood, and these questions are addressed here, in aiming to close the missing knowledge gap.

## Results

### Genomic Insights into Plasmid Transfer of the *mcr-1* Gene

**Fig 2:**
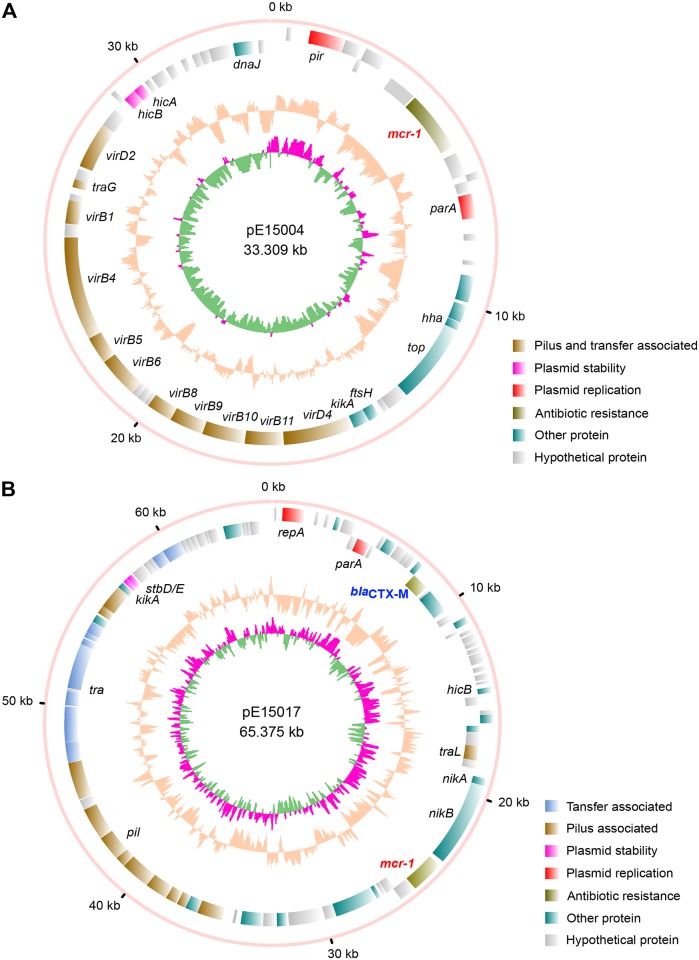
Scheme for the two *mcr*-*1*-harbouring plasmids pE15004 and pE15017. **A.** Genomic map of the *mcr-1*-containing IncX4-type plasmid pE15004 from the human gut microbiota. **B.** Genomic map of the MCR-1 and ESBL-coproducing IncI2-type plasmid pE15017 from the human gut microbiota. Circles from inside to outside indicate the GC screw, GC content and the open-reading frames in different DNA strands. The plasmid sequences were annotated by RAST, and the maps were generated using Circos program.

**Fig 3:**
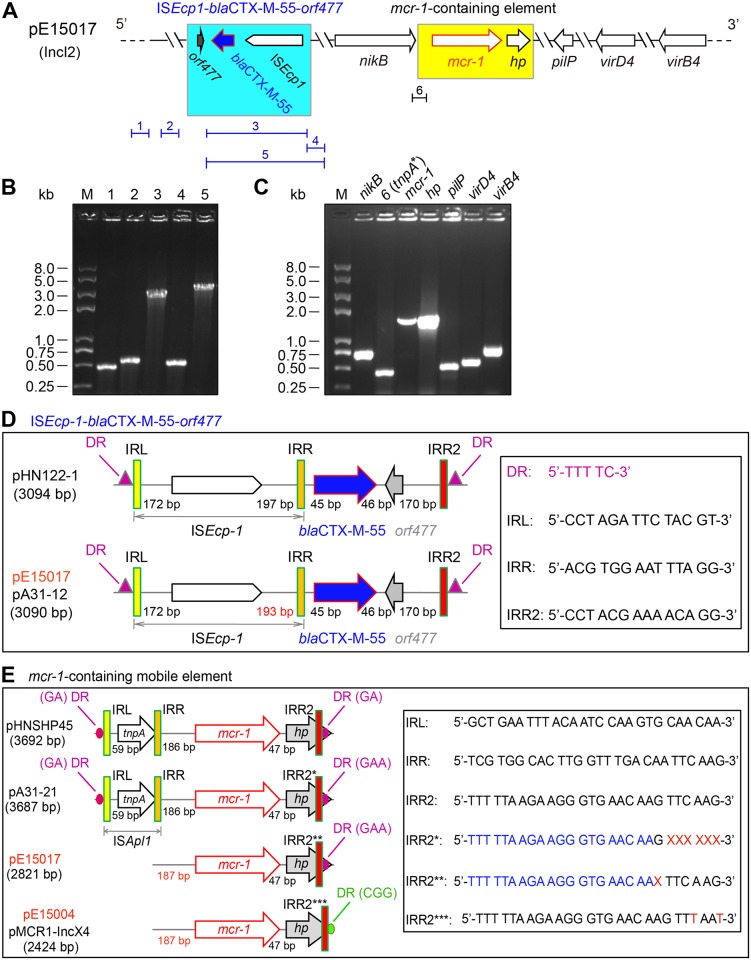
Genetic features of the two *mcr-1*-positive plasmids (pE15004 and pE15017). **A.** Schematic representation of the ESBL and MCR-1-coproducing plasmid pE15017. Arrows denote the genes with specific transcriptional direction. The *mcr-1* gene is in red, whereas the ESBL-encoding gene *bla*_CTX-M-55_ is indicated in blue. The cassette of IS*Ecp1*-*bla*_CTX-M-55_-*orf477* is highlighted in blue background, and the *mcr-1*-containing mobile element is under-scored in yellow background. The fragment “6” (earlier denoted as tnpA*) means the inter-space region adjacent to the 3’-end of the *tnpA*. A set of PCR primers (S2 Table) were designed to further confirm the presence of the two cassettes of “IS*Ecp1*-*bla*_CTX-M-55_-*orf477*” and “tnpA*-mcr-1-hp”, as well as their neighboring loci and/or virulence factors like *virD*4 [10]. **B.** PCR assays for the cassette of IS*Ecp1*-*bla*_CTX-M-55_-*orf477* and its neighboring loci on the plasmid pE15017. **C.** PCR-based detection of the *mcr-1* gene and other six loci. Designations: *nikB*, a relaxase for transposon; *pilP*, a Type IV pilus biogenesis protein; *virD4-virB4*, two genes encoding two components type IV secretion system; *tnpA*, a transposase-encoding gene; and hp, a hypothetical protein. M refers to Trans 2K Plus II DNA Ladder (TRANSGEN BIOTECH, Beijing, China), and kb denotes kilo-base pair. **D.** Scheme for genetic organization of the IS*Ecp1*-*bla*_CTX-M-55_-*orf477* operon from pE15017, pHN122-1 and pA31-12. The IS*Ecp1*-*bla*_CTX-M-55_-*orf477* cassettes on the two plasmids (pE15017 and pA31-12) are identical and only 4bp shorter than that of pHN122-1. **E.** Schematic representation of the *mcr-1*-containing mobile elements from the different plasmids. The insertion sequence IS*Apl1* occurs in both pHNSHP45 and pA31-12 is absent in the plasmid pE15017, which is validated by PCR detection coupled with Sanger sequencing. Similarly, no insertion sequence is found in front of the *mcr-1* gene in the two incX4-type plasmids pE15004 and pMCR1-IncX4. Unlike the DR (TTTTC) for the IS*Ecp1*-*bla*_CTX-M-55_-*orf477* (in **Panel D**), the DR sequences for the *mcr-1-hp* with/without IS*Apl1* (in **Panel E**) are divergent (GA in pHNSHP45, GA/GAA in the two IncI2 plasmids (pE15017 plus pA31-12), and CGG in the two IncX4 plasmids (pE15004 & pMCR1-IncX4)). Abbreviations: DR, Direct Repeats; IRL, l Inverted Repeats at Left; IRR, Inverted Repeats at Right. IRR2 is indicated with red rectangle. The sequences of the repeats are listed in the box on the right hand. X denotes the deleted nucleotide.

The recent emergence of colistin resistance may be attributed to MCR-1-mediated PEA addition of lipid A moiety at the 4’-phosphate group (Fig 1A), a component of bacterial LPS on the outer layer of outer-membrane for Gram-negative bacteria like *E*. *coli* (Fig 1B) [7]. Consistent with scenarios seen in *Neisseria* [2] and *E*. *coli* [7], our MS result suggested that the peak of lipid A with mass of 1797.4 is present in the colistin-susceptible *E*. *coli* MG1655 (S1A Fig), and one more peak of PPEA-4’-lipid A with mass of 1919.8 appears upon the arabinose-inducing expression of the MCR-1 enzyme (S1B Fig). It validated that the essence of MCR-1-catalyzed enzymatic reaction is the addition of PPEA (mass: 123 Au) to lipid A (mass: 1797.4–1797.6). Very recently, we successfully isolated *mcr-1*-harbouring plasmids from the colistin-resistant *E*. *coli* strains obtained from the gut microbiota of clinically diarrheal patients [10]. Here we subjected the *mcr-1*-positive plasmids to genome sequencing by next generation desktop MiSeq sequencer (Illumina). A pool of 350-bp paired-end reads we produced, were assembled with GS *De Novo* Assembler into two long contigs. We then checked the assembly of our plasmids (pE15004 and pE15017) through integrating raw data, PCR assays, and Sanger sequencing. The plasmid pE15004 was assembled correctly, while a ~2.2 kb fragment was missing in the original assembly of pE15017. Consequently, complete genomes of these two clinical *mcr*-*1*-positive plasmids (pE15004, in Fig 2A and pE15017 in Figs 2B and 3A–3D) were acquired.

The *mcr-1*-harbouring plasmid pE15004 was 33.309 kb in length with a GC content of 41.8%. This plasmid contained 51 predicted ORFs, among which 11 were associated with the formation of type IV pilus (Fig 2A). The backbone of pE15004 was closely-related to the *pir*-type *E*. *coli* plasmids pSAM7 from cattle in the United Kingdom and pJIE143 from human in Australia (S2 Fig), both of which are narrow-host-range IncX4-type plasmids [25,26]. Further comparative analysis indicated that plasmid pE15004 was nearly identical to IncX4 *mcr-1*-harboring *E*. *coli* plasmids pICBEC72Hmcr (Accession no.: CP015977) isolated from Brazil and pESTMCR (Accession no.: KU743383) from Estonia, *K*. *pneumoniae* plasmids pMCR1_Incx4 (Accession no.: KU761327) from China (Fig 3E) [27] and pMCR1.2-IT (Accession no.: KX236309) identified in Italy [28] (Table 1). Another *mcr-1*-bearing plasmid pE15017 (65.375 kb) we sequenced contained 91 ORFs (Fig 2B), sharing nearly all its sequences with the first-identified *mcr-1*-harbouring IncI2-type plasmid pHNSHP45 [7] (Fig 3 and S2 Fig). In comparison with the prototype *mcr*-*1*-positive plasmid pHNSHP45, the upstream insertion sequence IS*Apl1* flanked *mcr-1* was consistently missing in both pE15004 and pE15017(Fig 3E) as well as in other recently-reported *mcr-1*-containing plasmids, like pKH457-3-BE [22].

**Table 1:** Comparative analysis of two new plasmids (pE15004 and pE15017) with all the *mcr-1*-harbouring plasmids available from GenBank database

Species	Plasmid	Length (bp)	Type^a^	Accession No.	Country	IS*Apl1*	IS*683*	IS*Ecp1-bla*_CTX-M_
*E*. *coli*	pEC5-1	61,735	IncI2	CP016185	Malaysia	--	--	--
*E*. *coli*	pEC13-1	60,218	IncI2	CP016186	Malaysia	--	--	--
*E*. *coli*	pHNSHP45	64,015	IncI2	KP347127	China	+	+	--
*E*. *coli*	pECJP-61-63	63,656	IncI2	KX084393	China	--	--	--
*E*. *coli*	pS2.14–2	60,950	IncI2	CP016187	Malaysia	--	--	--
*E*. *coli*	pmcr1_IncI2	64,964	IncI2	KU761326	China	--	--	--
*E*. *coli*	pVT553	62,219	IncI2	KU870627	South Africa	+	--	--
*E*. *coli*	pABC149-MCR-1	61,228	IncI2	KX013538	United Arab Emirates	+	--	--
*E*. *coli*	pBA77-MCR-1	62,661	IncI2	KX013539	Bahrain	--	--	--
*E*. *coli*	pBA76-MCR-1	64,942	IncI2	KX013540	Bahrain	--	--	--
*E*. *coli*	pAF23	61,177	IncI2	KX032519	South Africa	+	--	--
*E*. *coli*	pA31-12	67,134	IncI2	KX034083	China	+	--	+
*E*. *coli*	pSLy1	65,888	IncI2	NZ_CP015913	USA	--	--	--
*Kluyvera ascorbata*	pMCR_1410	57,059	IncI2	KU922754	China	--	--	--
*Salmonella enterica*	pSCS23	65,419	IncI2	KU934209	China	+	--	+
***E*. *coli***	**pE15017**	**65,375**	**IncI2**	**This study**	**China**	**--**	**--**	**+**
*E*. *coli*	pECJP-B65-33	33,298	IncX4	KX084392	China	--	--	--
*E*. *coli*	pICBEC72Hmcr	33,304	IncX4	CP015977	Brazil	--	--	--
*E*. *coli*	pESTMCR	33,311	IncX4	KU743383	Estonia	--	--	--
*E*. *coli*	pAF48	31,808	IncX4	KX032520	South Africa	--	--	--
*E*. *coli*	pOW3E1	34,640	IncX4	KX129783	Switzerland	--	--	--
*E*. *coli*	pMCR1-NJ-IncX4	33,395	IncX4	KX447768	USA	--	--	--
*E*. *coli*	unnamed4	49,695	IncX4	NZ_CP016550	Netherlands	--	--	--
***E*. *coli***	**pE15004**	**33,309**	**IncX4**	**This study**	**China**	**--**	**--**	**--**
*K*. *pneumoniae*	pMCR1.2-IT	33,303	IncX4	KX236309	Italy	--	--	--
*K*. *pneumoniae*	pMCR1_IncX4	33,287	IncX4	KU761327	China	--	--	--
*E*. *coli*	pEC2_1–4	230,278	IncHI1B	CP016183	Malaysia	+	--	--
*E*. *coli*	pEC2-4	235,403	IncHI1B	CP016184	Malaysia	+	--	--
*E*. *coli*	pH226B	209,401	IncHI1B	KX129784	Switzerland	--	--	--
*E*. *coli*	pSA26-MCR-1	240,367	IncHI2	KU743384	Saudi Arabia	+	--	--
*E*. *coli*	pECJP-59-244	243,572	IncHI2A	KX084394	China	+	--	--
*E*. *coli*	pS38	247,885	IncHI2A	KX129782	Switzerland	+	--	+
*E*. *coli*	pHNSHP45-2	251,493	IncHI2A	KU341381	China	+	--	--
*E*. *coli*	pKP81-BE	91,041	IncFII	KU994859	Belgium	+	--	--
*E*. *coli*	pKH457-3-BE	79,798	IncFII	KU353730	Belgium	--	--	--
*E*. *coli*	pMR0516mcr	225,069	IncFIB	KX276657	USA	+	--	+
*E*. *coli*	unnamed1	15,998	unknown	KX528699	Vietnam	+	--	--
*E*. *coli*	p100R	26,403	unknown	KX090925	Switzerland	+	--	--

^a^ all the *mcr-1*-carrying plasmids were extracted from the GenBank database by 16 Aug, 2016 and annotated by PlasmidFinder-1.3 server (https://cge.cbs.dtu.dk/services/PlasmidFinder/). The positive sign (+) denotes the presence of IS*Apl1* (and/or IS*683/* IS*Ecp1-bla*_CTX-M_), whereas not for the negative sign (--).

Besides the *mcr-1* gene (Fig 3A and 3C), our PCR assays combined with Sanger sequencing determined that pE15017 carries an extended-spectrum β lactamase (ESBL) gene *bla*_CTX-M-55_ (Fig 3A and 3B). Thus pE15017 represents an ESBL and MCR-1-coproducing plasmid (Fig 3A, 3D and 3E). Previously, the co-occurrence of ESBL and MCR-1 had been found on the IncHI2-type plasmids from *E*. *coli* and *Salmonella enterica* [20,29]. Similar to the other *mcr-1*-carrying plasmid, pA31-12 (Fig 3D) [30], pE15017 might be an additive example of IncI2-type plasmid with above two antibiotic resistance determinants. Interestingly, four base pair (AACA, 1612–1615) is consistently missed in the IS*Ecp1*-*bla*_CTX-M-55_-*orf477* operon (3090 bp) in both pE15017 and pA31-12, in relative to the counterpart (3094 bp) in pHN122-1(Fig 3D) [31]. In fact, the IS*Ecp1*-*bla*_CTX-M-55_ transposition unit flanked by DR was also recently observed in the *Salmonella* plasmid pSCS23 (KU934209) as shown in Fig 3 [29].

Taken together, these results indicate that plasmid pE15004 is an additive member of the *mcr-1*-carrying plasmids, while pE15017 together with other recently identified plasmids such as pmcr1_IncI2 (Accession no.: KU761326) [23] and pBA76-MCR-1(Accession no.: KX013540), are recent variants of plasmid pHNSHP45 (Figs 2, 3E and S2 Fig). Furthermore, the *mcr-1* gene has been found carried by plasmids belonging to at least 5 different incompatibility groups (Table 1), verifying a trend of diversification [10]. Though it is currently only found in Enterobacteriaceae, its dissemination to broad host range plasmids and subsequent spread to a broad bacterial host range cannot be excluded. Moreover, comparative analysis showed that nearly identical *mcr-1*-containing plasmids were discovered in different countries, suggesting that, besides the possibility that the dissemination of the *mcr-1* gene was captured independently from a common ancestor [11], the direct spread of bacteria harboring the same plasmid is not impossible.

### Functional dissection of MCR-1 colistin resistance

Although Liu and coworkers determined that the expression of plasmid-borne *mcr*-*1* confers colistin resistance to certain species of the *Enterobacteriaceae* family [7], functional details of the MCR-1 protein are poorly understood. Here, we attempt to address this issue. Philius Transmembrane Prediction Server (http://www.yeastrc.org/philius/pages/philius/runPhilius.jsp) suggested that the MCR-1 protein is an integral membrane protein with five trans-membrane regions (S3A Fig). Similar to the LptA (EptA) of *Neisseria*, the multiple sequence alignments indicated that the MCR-1 protein also belongs to a family of phosphoethanolamine lipid A (PEA) transferases with putative conserved sites (E246, T285, H395, D465 and H466, in S3B Fig) required for its catalytic activity, *i*.*e*., the addition of PEA to lipid A from phosphatidylethanolamine (Fig 1). Because the fact that the nascent LPS in cytoplasm is flipped by the ABC transporter MsbA into periplasm [32] and the covalent modification of the lipid A component on LPS occurs in periplasm [1], it is speculated that the trans-membrane regions ensures the correct anchoring of the MCR-1 enzyme to the periplasmic face of the cytoplasmic membrane attached to the catalytic domain of PEA transferase. While, experimental evidence for this hypothesis is lacking, we aimed to address them using the integrative approaches ranging from protein biochemistry, bioinformatics and structural biology to bacterial genetics.

We over-expressed the membrane protein MCR-1 and purified it to homogeneity (S4A Fig) and confirmed this by Western blot using an anti-6x-His primary antibody (S4B Fig). MS-based identification further confirmed the identity of the recombinant MCR-1 trans-membrane protein (S4C Fig). To further gain structural insights into the biochemical mechanism of MCR-1, we applied both protein engineering and structure-guided mutagenesis. In particular, the arabinose-inducible expression system pBAD24/MG1655 was also utilized to probe the above concerns *in vivo*. Given the fact that i) the chemical modification phosphate group of lipid A at 1 or 4’-position impair its binding to polymyxins [2], ii) the newly-synthesized LPS is translocated by the MsbA lipid flippase into periplasm from cytoplasm [1,32]; iii) bacterial periplasm is the only place where the moiety of lipid A on LPS is covalently modified with either 4-amino-arabinose or phosphoethanolamine [2], it is prerequisite that the enzyme modifier including MCR-1 should be localized in bacterial periplasm. Thus, we are extremely interested in determining physiological role of the trans-membrane region in MCR-1 function.

Firstly, MG1655 with/without the empty vector pBAD24 (the negative control) fails to grow on the LBA plates with above 2 mg/L of colistin, whereas the positive control, MG1655 with the arabinose-induced expression of *mcr*-*1*, can grow well on the solid media with 16–32 mg/L of colistin (Table 2). In contrast, pBAD24-facilitated expression of *Neisseria lptA* conferred the colistin-susceptible MG1655 strain with an ability to grow on the LBA plates with 8–16 mg/L colistin (Table 2). Though the *Neisseria lptA* encodes the colistin resistance at an appreciable level, the amplitude of drug resistance is less than that of the MCR-1 (Table 2). Subsequently, we engineered a deletion mutant of the *mcr-1* gene (Δtm) whose protein product lacks the N-terminal trans-membrane region to further evaluate its role *in vivo*. Similar to the scenario with the negative control, we found that the *E*. *coli* strain with the araninose-induced expression of the *mcr*-*1* mutant (Δtm) cannot grow on the LBA plates with over 2 mg/L of colistin (Table 2), validating the importance of the transmembrane region in the MCR-1-mediated colistin resistance. Thus it may be concluded that the catalytic activity for PEA transferase depends on its location of MCR-1 on bacterial inner-membrane.

**Table 2:** Comparison of the MCR-1 (wild type, point mutation and transmembrane deletion mutant) and the *Neisseria* LptA in the ability of colistin resistance

MIC (mg/L)									
MG1655	MG1655+Vec	LptA	MCR-1						
			WT	ΔTM	E246A	T285A	H395A	D465A	H466A
1–2	1–2	8–16	16-32	2-4	~2	~2	2~4	~2	~2

**Fig 4:**
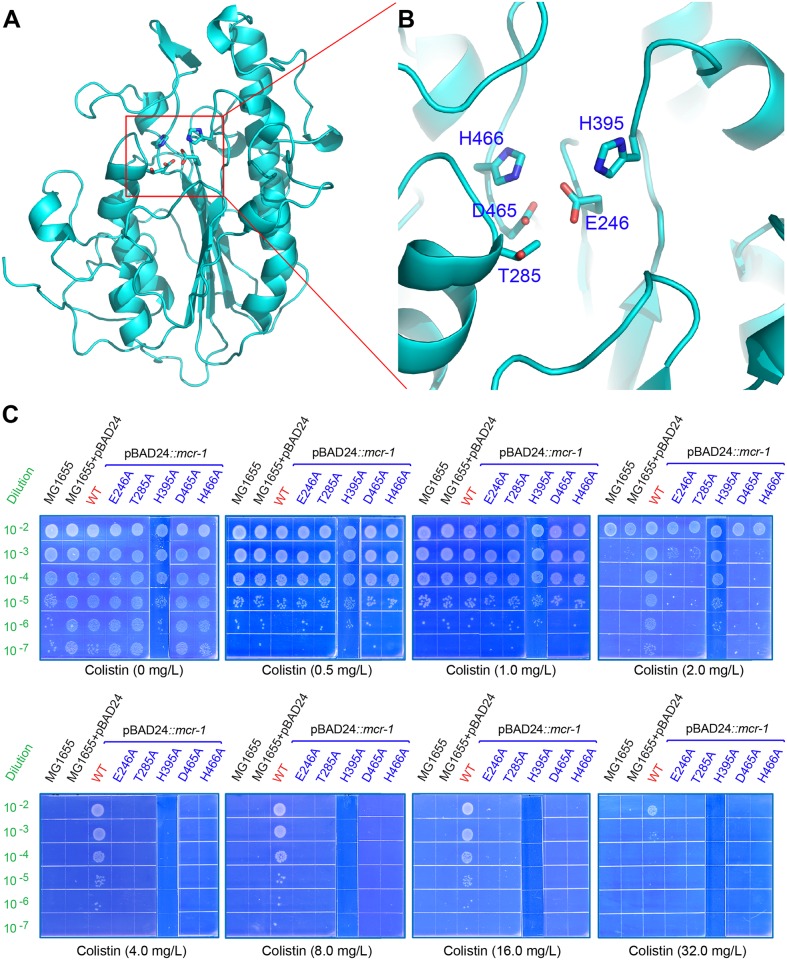
Structure-guided determination of five important residues for MCR-1 mediated colistin resistance. **A.** The modeled ribbon structure for PEA-lipid A transferase domain of the membrane-bound MCR-1 protein. The ribbon structure was given via PyMol software. The key residues proposed by structural docking is indicated with red rectangle. **B.** The enlarged view of the five crucial residues for PEA-lipid A transferase activity of the MCR-1 protein. The five important residues include E246, T285, H395, D465 and H466, respectively. **C.** Structural-guided functional determination of the five residues (E246, T285, H395, D465 and H466) essential for MCR-1-mediated colistin resistance. A representative result of three independent experiments is given. Note: **panel C** is generated using the photograph combined with two plates because that plate size is limited and not allowed us to spot all the samples in a same plate.

To determine the minimum inhibitory concentration of colistin, the mig-log phase cultures (OD600 = 0.7–1.0) in serial dilution were spotted on LBA plates supplemented with colistin at varied level (0, 0.5, 1.0, 2.0, 4.0, 8.0, 16.0 and 32.0 mg/L) and kept overnight at 37°C. In addition to the five strains used in Fig 4, two more *E*. *coli* strains are tested that carry either pBAD::*mcr*-*1*(Δtm) or pBAD::*lptA* (S1 Table).

Expression of both *lptA* and *mcr*-*1* (and its mutants) is induced by the addition of 0.2% arabinose into LBA media. Designations: MIC, minimum inhibitory concentration; TM (tm), transmembrane region; Vec, pBAD24.

Using the *Neisseria* Lipo-oligosaccharide Phosphoethanolamine Transferase A (LptA) as structural template (PDB: 4KAV) [2], structural modeling by Swiss-Model program allow us to visualize the architecture of PEA transferase domain of the membrane-bound MCR-1 enzyme (Fig 4A). Extensive analyses of structural docking together with sequence alignments allow us to hypothesize that the following five residues (E246, T285, H395, D465, and H466) are critical for the substrate binding of MCR-1, and in turn determines the MCR-1-encoded colistin resistance (Fig 4B). Driven by this speculation, we used site-directed PCR mutagenesis to create the following point mutations (E246A, T285A, H395A, D465A, and H466A). In contrast to the positive control carrying the wild-type *mcr-1* gene (Fig 4C), none of the MG1655 strains expressing the *mcr*-*1* point mutants were observed to grow significantly on the condition of above 2.0 mg/L of colistin, which is almost identical to that of negative control (Fig 4C and Table 2). This represents *in vivo* evidence that the five residues are essential for the function of MCR-1.

### Evolutionary Analyses of the MCR-1 Protein

A BLASTp search for MCR1 and *Neisseria gonorrhoeae* LptA (EptA) returned a large set of divergently related sequences (annotated as PE transferases, Sulfatases or membrane proteins. Detailed comparisons of alignment methods applied to divergently related sequences have produced low-accuracy results with sequence identities below 30% [33]. We have thus limited our search scope to 30%. To determine a phylogenetic profile of MCR-1, Multiple sequence alignment of the dataset was performed by *MUSCLE* (http://www.ebi.ac.uk/Tools/msa/muscle) [34] using default parameters and the quality of the alignment was evaluated using Guidance (http://guidance.tau.ac.il) [35]. Consequently, we retrieved 32 candidate proteins that returned hits with >30% identity. Maximum Likelihood (ML) phylogenic trees were reconstructed by using the LG amino acid substitution model with gamma distribution and invariant sites selected using *MEGA* version 6 [36]. To ensure appreciable reliability, the results we obtained were validated by 1000 bootstrap replicates.

**Fig 5:**
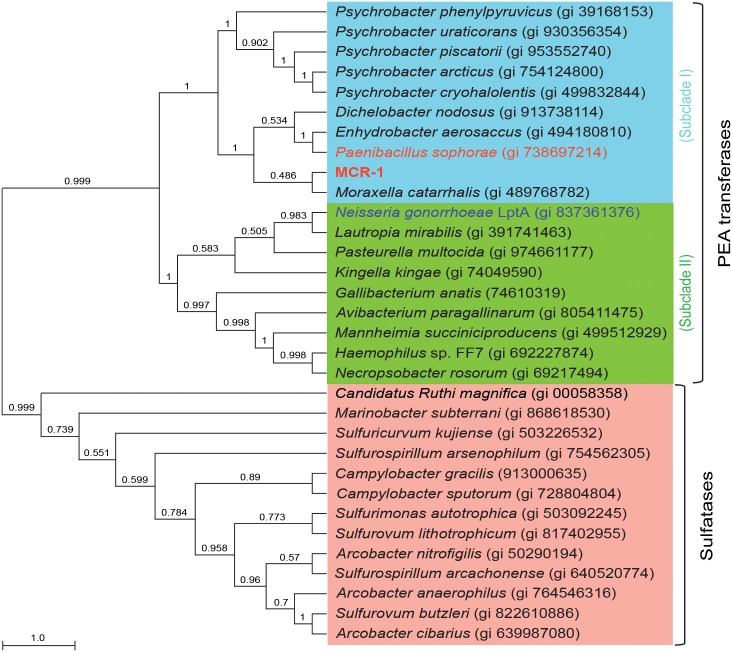
Phylogeny of MCR-1. The method of maximum likelihood tree is applied here. The scale bar corresponds to a 100% difference in compared residues, on average, per branch length. The members in this phylogenetic tree can be grouped into two clades (one is annotated as Phosphoethanolamine transferases [PEA transferase], and the other denotes Sulfatases [marked in pink]. Of note, the group of PEA transferase can be divided into two sub-clades: Sub-clade I (marked in blue) with MCR-1 and Sub-clade II (highlighted in green) with *Neisseria gonorrhoeae* LptA.

Intriguingly, the reconstruction of a maximum phylogeny tree using 32 unique proteins selected from the BLASTp search allowed us to clearly observe two distinctive clades: one containing a family of PEA transferases including MCR-1 and *Neisseria* LptA (Fig 5) and the other containing an array of putative sulfatases (Fig 5). Also, the members of the PEA transferase family are divided into two apparent subclades, one of which features MCR-1, and the other one comprising *Neisseria* LptA (Fig 5). The chromosomally-encoded LptA from *Neisseria* species is most-closely clustered with putative integral membrane proteins found in other pathogenic Ɣ-proteobacteria, whereas the plasmid-borne MCR-1 on the other hand is very close to PEA transferases from the colistin-producing bacteria, *esp*. the *Paenibacillus* species (Fig 5) [37–39]. Despite the fact that MCR-1 and LptA share very low sequence identity to each other and fall into two separate subclades within the tree (Fig 5), they still remain functionally-equivalent (Table 2).

The phylogenetic tree here indicates a divergent evolutionary pattern between the LptA/MCR-1 integral membrane proteins and other putative sulfatases. A domain analysis of MCR-1 revealed distinct trans-membrane helices followed by a sulfatase domain. Sulfatases that catalyze the hydrolysis of a sulfate group are present in all three domains of life and constitute a heterogenic group of enzymes [40,41]. Due to similarity in size between a sulfate and a phosphate group, one can easily imagine why PEA transferases share core catalytic features with sulfatases. In fact, closely-related sulfotransferases from *Mycobacterium* transfer a sulfate group into the glycolpeptidolipids (GPL), the equivalent of the LPS in gram-negative bacteria [42]. Due to lack of sufficient sequence data and experimental validation, it is hard to trace the ancestry of MCR-1 to its chromosomal origins. Given the fact that removal of the trans-membrane region from the MCR-1 protein damages its function of MCR-1-mediated colistin resistance (Table 2), one can speculate that acquisition of a trans-membrane domain could have easily enabled these PEA transferases to correctly localize in the inner membrane and to eventually target a variety of substrates with different implications ranging from cationic antimicrobial peptide resistance in the case of lipid A modification, to changes in motility when FlgG, a flagella rod protein, is modified. Given the fact that phylogenetic tree places the MCR-1 protein very close to the PEA transferases from the *Paenibacillus* family, the known producers of polymyxins, but in a different sub-clade than *Neisseria* LptA (Fig 5), it raises the possibility that 1) the cousins of *Paenibacillus* might be highly relevant to its origin of the MCR-1; 2) a potentially parallel evolutionary path is implicated for the two genes (*mcr*-*1* and *lptA*) under similar environmental selection pressures, *e*.*g*., the massive use of colistin as a veterinary medicine.

## Discussion

The data we present represents a first comprehensive glimpse of mechanisms for diversified plasmid transfer, evolutionary origin, and catalytic reaction of the MCR-1-mediated colistin resistance. The discovery of new *mcr-1*-harbouring plasmids (pE15004 and pE15017) adds new knowledge into the newly-emerging field of MCR-1 and colistin resistance, furthering our understanding of the diversity in the dissemination of the *mcr*-*1* gene [11]. Unlike the paradigm *mcr-1*-positive plasmid pHNSHP45 that is isolated from a swine *E*. *coli* in southern China, the two plasmids we reported are extracted from clinical *E*. *coli* isolates of diarrhea patients. Further functional definition of plasmid genomes delineated that 1) the plasmid pE15004 is a IncX4 plasmid of around 33kb long [25,26], differing from the IncI2-type plasmid pHNSHP45 of about 64kb in length [7] (Figs 2, 3 and S2 Fig); 2) the other plasmid pE15017 (~65kb) seemed to be a recent variant of pHNSHP45 (~64kb) in that an insertion sequence IS*Apl1* of around 1 kb in front of *mcr*-*1* gene of pHNSHP45 is absent in pE15017 (Fig 3E) and an ESBL encoding gene was captured (Fig 3A, 3B and 3D). In agreement with proposal by Petrillo and coauthors [43], the deletion of this IS*Apl1*insertion sequence might be the relic of the *mcr-1* dissemination. It was reported that the *mcr*-*1* colistin resistance gene is present in a multidrug-resistant plasmid [22] or coexists with other resistance genes like extended spectrum β-lactamase [16,19–21], and even the notorious NDM-1 [44] and its variants (NDM-5 [23] and NDM-9 [24]). We also found that the pE15017 is an ESBL and MCR-1 coproducing plasmid similar to pA31-12, a recently-isolated plasmid from China [30]. These facts imply that multi-drug, even pan-drug, resistant bacteria with colistin resistance will eventually evolve, a fact that deserves close attention. By contrast, the pE15017 plasmid carries both ESBL and MCR-1. Consistent with our recent observations with swine lung microbiota [45], the results highlight the differences amongst the *mcr*-*1*-carrying plasmid reservoirs in human gut microbiota [10].

We experimentally validated that the expression of *Neisseria* LptA augments colistin resistance of *E*. *coli* (Table 2), despite being weaker than MCR-1 (Table 2), suggesting the possibility of various catalytic aspects and differing evolutionary paths for the two genes (*lptA* and *mcr*-*1*). To address this concern, we conducted phylogenetic analyses and found they are placed into two neighboring sub-clades of the PEA transferase family (Fig 5), giving a strong implication of parallel evolutionary paths for the two genes (*mcr*-*1* and *lptA*). Additionally, the functional impairment of the MCR-1 colistin resistance by the removal of the trans-membrane regions demonstrates that membrane anchoring of the soluble catalytic domain (PEA transferase) is essential its function (Table 2). In particular, we also determined the requirement for the five motif-forming residues for MCR-1 function (Table 2 and Fig 4), which might facilitate the binding of this enzyme to its cofactor of zinc ions. The mechanistic insights we obtained definitely extended our knowledge on MCR-1/colistin resistance, and might provide a molecular basis for the development of inhibitors/drugs of small molecule via bypassing the MCR-1-mediated colistin resistance in the post-antibiotics era.

Generally, the resistance to colistin is correlated with the decrement in affinity of the lipid A group of lipopolysaccharide to the polymyxin antibiotics. Unlike the chromosome-encoded mechanism with the involvement of a two-component systems (*pmrAB* [8] and *phoPQ* [6]) and the regulator *mgrB* [6], the plasmid-borne MCR-1 represents an newly-emerging machinery for colistin resistance in which the modification of lipid A is catalyzed by the MCR-1 enzyme, giving the reduced affinity to polymyxin (Fig 1) [7]. It seems likely that the current situation of MCR-1 colistin resistance worldwide has been over-underestimated since almost 30 countries have been identified to have the *mcr*-*1* gene present in the past several months [5,10]. Given the fact that i) colistin is the last line of refuge amongst therapeutics against lethal infections by multidrug-resistant Gram-negative pathogens [3,46]; and ii) the extensive consumption of colistin as a veterinary medicine in the poultry/swine production worldwide functions as a strong selective pressure which then imposes a risky burden on food safety and public health, it is urgently needed to reconsider appropriate use of colistin in veterinary/human medicine and restrict global dissemination of the *mcr*-*1* colistin resistance gene transferred by diversified plasmids. In summary, our findings provide a functional glimpse of plasmid transfer, evolutional origin, and catalysis mechanism for the MCR-1 colistin resistance.

## Materials and Methods

### Strains and Growth Conditions

The clinical *E*. *coli* strains from gut microbiota of diarrhea patients (kindly provided by Shenzhen Centre for Diseases Control, China [10]) were grown in the liquid Luria-Bertani (LB) media for the isolation of *mcr*-*1*-positive plasmids. The two genetically-modified strains DH5α and BL21 (DE3) were separately applied for gene cloning and protein expression (S1 Table). The colistin-susceptible strain of *E*. *coli* MG1655 was used for functional assays for the *mcr*-*1* gene and/or its mutants (S1 Table). The solid LB agar plates supplemented with appropriate antibiotics were applied to either screen possible positive clones for the presence of the *mcr*-*1* gene or determine the minimum inhibitory concentration of colistin by expression of MCR-1.

### Plasmids and Genetic Manipulations

The plasmids were isolated routinely from *E*. *coli* strains using an alkaline lysis method. Using specific primers (S2 Table), PCR screening was performed for the presence of *mcr*-*1* gene in the colistin-resistant strains. The full coding sequence of *mcr*-*1* was then cloned in pET28(a) via the two cuts (BamHI plus XhoI), giving the recombinant plasmid pET28::*mcr*-*1* (S1 Table). Both the wild type of *mcr-1* and its deletion mutant, *mcr*-*1*(Δtm) were directly inserted into the two cuts (EcoRI and SalI) of an arabinose-inducible expression vector pBAD24, giving the plasmids of pBAD24::*mcr*-*1* and pBAD24::*mcr*-*1*(Δtm), respectively (S1 Table). Similarly, the recombinant plasmid pBAD24::*lptA* was constructed through cloning of the *Neisseria* LptA-encoding gene into pBAD24 (S1 Table). Using the pBAD24::*mcr*-*1* plasmid as the template, The experiments of site-directed PCR mutagenesis were conducted as we earlier described [47]. All the acquired plasmids were verified by PCR assays and direct DNA sequencing.

### Measurement of Colistin Resistance/Tolerance

The minimal inhibitory concentration (MIC) of colistin was determined using liquid broth dilution test as recommended by EUCAST with Cation-adjusted Mueller-Hinton Broth. And survival ability of *E*. *coli* expressing different protein was determined as follows: mid-log phase cultures diluted appropriately were spotted on LBA plates supplemented with colistin at varied level (ranging from 0, 0.5, 1.0, 2.0, 4.0, 8.0, 16.0 to 32.0 mg/L), and maintained at 37°C overnight. In the assays for colistin resistance/tolerance, the colistin-susceptible strain MG1655 acted as the negative control, the MG1655 strain carrying the empty vector, pBAD24, referred to blank control, and the MG1655 strain with pBAD24::*mcr*-*1* is the positive control. All the other strains that expressed either *Neisseria* LptA, the trans-membrane deletion mutant of *mcr*-*1*(Δtm), or one of and five mutants of *mcr-1* with single point mutation, were tested for their different ability of colistin resistance. 0.2% arabinose was added into the LBA plates to induce the expression of *lptA/mcr-1* (and/or its mutants).

### LPS Extraction and MS-based Identification

Ultrapure LPS was extracted using the hot phenol method as described by two different groups [2,7] with minor modifications. Briefly, overnight *E*. *coli* cultures (~10 ml) collected by centrifugation were washed with 5 ml of 50% cold acetone before re-suspending in 0.55 ml of water at 70°C. It was then mixed with 0.45ml of phenol (pre-warmed to 70°C) by vigorous vortexing. This mixture was incubated at 70°C for 30 min before spinning at 16,000x g for 15 min to collect the aqueous phase. 1.3 ml of cold absolute ethanol, 6.7 μl of 3 M sodium-acetate and water were added till a final volume of 1.9 ml. This was incubated at -80°C for 15mins to precipitate the crude LPS.

The resultant crude LPS from the aqueous phase was dialyzed against de-ionized water using the aqueous phase 1000 MWCO dialysis tubing. After dialysis, the samples were freeze-dried, and re-suspended in 55 μl wash solution (20 mM Tris-HCl (pH 8.0), 2 mM MgCl2, DNase I (100 μl of 7 mg/ml) and RNase A (100 μl of 17 mg/ml)). The mixture was maintained for 3 h at 37°C prior to adding 5μl of Proteinase K and further incubating it at 56°C for 1hr. An equal amount of phenol was mixed with the mixture and centrifuged for 30mins at 16,000x g to collect the aqueous phase. 193 μl of 50 mM TRIS, 7 μl of 3M sodium acetate (pH 5.2) and 3 volumes of cold ethanol were added to the aqueous phase and incubated at -80°C for 15mins to precipitate the LPS. The precipitated LPS was collected by centrifugation at 16,000x g for 15 mins and re-suspended in 50 μl water. Finally, Lipid A samples were assayed with electrospray ionisation mass spectrometry (ESI-MS) as Liu *et al*. [7] reported.

### MiSeq Sequencing of Plasmid Genomes

The *mcr*-*1*-positive plasmids that met the requirement of quality control were subjected to library preparation prior to the whole genome sequencing. The next-generation Illumina MiSeq sequencing was conducted as per protocols recommended by the manufacturer, generating a pool of 350-bp paired-end reads. The draft assembly of plasmids was performed with GS De Novo Assembler to give two long contigs. PCR and Sanger sequencing were then performed to verify and correct the contigs. As a result, full genomes of the two plasmids of clinical origins (pE15004 and pE15017) were produced. The plasmid sequences were annotated by RAST, and the genome maps were drawn with the Circos program. Comparative genomics of plasmids were carried out with the tools,Glimmer and BLAST, to probe the potential origin/mechanism for transfer of the *mcr*-*1*-carring plasmids.

### Sequence Acquisition and Alignment of MCR-1

To identify sequences homologous to MCR-1, a *BLASTp* search was performed using the amino acid sequence of MCR-1 and *Neisseria gonorrhoeae* LptA (formerly named EptA) as a query. In order to avoid hits from very closely related species, *Escherichia coli* and uncultured environmental samples were excluded from the search and the max target sequences acquired were 500. The top unique protein sequences were selected and submitted to the web-based program, *Guidance* (http://guidance.tau.ac.il) [35], to evaluate the quality of alignment and to identify potential regions and sequences reducing the quality of alignment. Multiple sequence alignment was performed using *MUSCLE* with default parameters (http://www.ebi.ac.uk/Tools/msa/muscle/) [34]. The alignment was also manually evaluated and adjustments were made as necessary.

### Phylogenetic Analysis

The best amino acid substitution model to be used for reconstructing a tree was identified using the Models function in *MEGA* version 6 [36]. The model with the least score (LG with G+I) was used to reconstruct Maximum Likelihood trees while treating gaps/missing data as partial deletions. Results were validated using 1000 bootstrap replicates.

### Bioinformatics analyses

Philius Transmembrane Prediction Server (http://www.yeastrc.org/philius/pages/philius/runPhilius.jsp) was applied to probe the topological structure of the MCR-1 protein. The protein sequences of MCR-1 and the related proteins were subjected to the program of Clustal Omega (http://www.ebi.ac.uk/Tools/msa/clustalo/), and the final output of the multiple sequence alignments was given processed by the program ESPript 2.2 http://espript.ibcp.fr/ESPript/cgi-bin/ESPript.cgi) [48]. Structural modeling for the PEA-lipid A transferase domain of MCR-1 was processed by Swiss-Model program, using the *Neisseria* Lipo-oligosaccharide Phosphoethanolamine Transferase A (LptA) as structural template (PDB: 4KAV) [2], the resultant result in ribbon structure was given via PyMol software. Plasmid typing was performed with the help of PlasmidFinder-1.3 server (https://cge.cbs.dtu.dk/services/PlasmidFinder/).

### Nucleotide Sequence Accession Number

The genome sequences of the two plasmids (pE15004 & pE15017) were deposited into the GenBank database with the accession no., KX772777 and KX772778, respectively.

## Supporting Information

S1 Text Genome sequence of the plasmid pE15004. (DOCX)

S2 Text Genome sequence of the plasmid pE15017. (DOCX)

S1 Table Strains and plasmids in this study. (DOCX)

S2 Table Primers used in this study. (DOCX)

S1 Fig MS verification of the MCR-1 enzymatic activity. **A**. ESI-MS-based analyses for the lipid A profile of LPS extracted from the negative control *E*. *coli* MG1655 strain carrying empty vector pBAD24
**B**. ESI-MS determination of the LPS Lipid A components from the *E*. *coli* MG1655 strain with the plasmid pBAD24::*mcr*-*1*
Bis-phosphorylated hexa-acylated lipid A (m/z = ~1797) and the mono-phosphorylated derivative (m/z = ~1717) were detected in the two *E*. *coli* strains. The MG1655 strain with the expression of the *mcr*-1 gene was consistent with one PEA added to the bis-phosphorylated structure (m/z = 1920; i.e., 1797 + 123).
(TIF)

S2 Fig Comparative analysis of representative *mcr-1*-carrying plasmids. The comparative genomic analysis was performed using Mauve alignment software [49]. The alignment was shown using Mauve’s locally collinear blocks (LCBs). Each LCB is a homologous region that was shared by two or more plasmids. The comparative was viewed using the solid LCB coloring style, that is, solid color was drawn for each LCB. Open Reading Frames (ORFs) were displayed as blank rectangles below each plasmid sequence, and the solid red rectangles indicate the *mcr-1* gene.
(TIF)

S3 Fig Bioinformatics analyses for the MCR-1 colistin resistance protein. **A**. Transmembrane prediction for the MCR-1 protein
**B.** Multiple sequence alignments of the MCR-1 protein with the *Neisseria* LptA protein
The topological structure of the MCR-1 protein was predicted with Philius Transmembrane Prediction Server (http://www.yeastrc.org/philius/pages/philius/runPhilius.jsp). The alignment of protein sequences was conducted using Clustal Omega (http://www.ebi.ac.uk/Tools/msa/clustalo/), and the output was given following the process by the program ESPript 2.2 (http://espript.ibcp.fr/ESPript/cgi-bin/ESPript.cgi) [48]. Identical residues are in white letters with red background, similar residues are in the form with mixture of red/black letters, and the varied residues are in black letters.
The important residues critical for Zn^2+^ binding and/or substrate binding are highlighted with arrows. Abbreviations: TM, Tran-smembrane; PEA, Phosphoethanolamine; Nm, *Neisseria meningitidis*; Ng, *Neisseria gonorrhoeae*, LptA, Lipid A PEA transferase
(TIF)

S4 Fig Visualization for the transmembrane protein MCR-1. **A.** SDS-PAGE profile for the purified transmembrane protein MCR-1
**B.** Western blot analyses for the purified MCR-1 protein with the anti-6XHis tag primary antibody
Designations: M, marker; kDa, kilo-dalton; WB, western blot.
**C.** MS verification of the recombinant MCR-1 protein
The tryptic peptides with 79.5% coverage to the MCR-1 sequence are given in bold and underlined type.
(TIF)

## References

[1] Raetz CR, Guan Z, Ingram BO, Six DA, Song F, et al (2009) Discovery of new biosynthetic pathways: the lipid A story. J Lipid Res 50 Suppl: S103–108. 18974037 10.1194/jlr.R800060-JLR200 PMC2674688

[2] Wanty C, Anandan A, Piek S, Walshe J, Ganguly J, et al (2013) The structure of the neisserial lipooligosaccharide phosphoethanolamine transferase A (LptA) required for resistance to polymyxin. J Mol Biol 425: 3389–3402. 10.1016/j.jmb.2013.06.029 23810904

[3] Paterson DL, Harris PN (2016) Colistin resistance: a major breach in our last line of defence. Lancet Infect Dis 16: 132–133. 10.1016/S1473-3099(15)00463-6 26603171

[4] Baron S, Hadjadj L, Rolain JM, Olaitan AO (2016) Molecular mechanisms of polymyxin resistance: knowns and unknowns. Int J Antimicrob Agents 10.1016/j.ijantimicag.2016.06.023. [Epub ahead of print]: S0924-8579(0916)30193-30195. 27524102

[5] Schwarz S, Johnson AP (2016) Transferable resistance to colistin: a new but old threat. J Antimicrob Chemother 71: 2066–2070. 10.1093/jac/dkw274 27342545

[6] Cannatelli A, D'Andrea MM, Giani T, Di Pilato V, Arena F, et al (2013) *In vivo* emergence of colistin resistance in *Klebsiella pneumoniae* producing KPC-type carbapenemases mediated by insertional inactivation of the PhoQ/PhoP *mgrB* regulator. Antimicrob Agents Chemother 57: 5521–5526. 10.1128/AAC.01480-13 23979739 PMC3811314

[7] Liu YY, Wang Y, Walsh TR, Yi LX, Zhang R, et al (2016) Emergence of plasmid-mediated colistin resistance mechanism MCR-1 in animals and human beings in China: a microbiological and molecular biological study. Lancet Infect Dis 16: 161–168. 10.1016/S1473-3099(15)00424-7 26603172

[8] Gunn JS (2008) The *Salmonella PmrAB* regulon: lipopolysaccharide modifications, antimicrobial peptide resistance and more. Trends Microbiol 16: 284–290. 10.1016/j.tim.2008.03.007 18467098

[9] Hasman H, Hammerum AM, Hansen F, Hendriksen RS, Olesen B, et al (2015) Detection of *mcr*-*1* encoding plasmid-mediated colistin-resistant *Escherichia coli* isolates from human bloodstream infection and imported chicken meat, Denmark 2015. Euro Surveill 20. 10.2807/1560-7917.ES.2015.20.49.30085 26676364

[10] Ye H, Li Y, Li Z, Gao R, Zhang H, et al (2016) Diversified *mcr-1*-harbouring plasmid reservoirs confer resistance to colistin in human gut microbiota. mBio 7: e00177 10.1128/mBio.00177-16 27048797 PMC4817250

[11] Hu Y, Liu F, Lin IY, Gao GF, Zhu B (2016) Dissemination of the *mcr*-*1* colistin resistance gene. Lancet Infect Dis 16: 146–147. 10.1016/S1473-3099(15)00533-2 26711359

[12] Zeng KJ, Doi Y, Patil S, Huang X, Tian GB (2016) Emergence of plasmid-mediated *mcr*-1 gene in colistin-resistant *Enterobacter aerogenes* and *Enterobacter cloacae* . Antimicrob Agents Chemother 60: 3862–3863. 10.1128/AAC.00345-16 26976876 PMC4879368

[13] Yang C (2015) Emergence and spread of a plasmid-mediated polymyxin resistance mechanism, MCR-1: Are bacteria winning? Infectious Diseases and Translational Medicine 1(2): 56.

[14] Gao R, Li Y, Lin J, Tan C, Feng Y (2016) Unexpected complexity of multidrug resistance in the *mcr-1*-harbouring *Escherichia coli* . Sci China Life Sci 59: 732–734. 10.1007/s11427-016-5070-1 27178474

[15] Gao R, Wang Q, Li P, Li Z, Feng Y (2016) Genome sequence and characteristics of plasmid pWH12, a variant of the *mcr-1*-harbouring plasmid pHNSHP45, from the multi-drug resistant *E*. *coli* . Virulence 7: 732–735. 10.1080/21505594.2016.1193279 27221541 PMC4991314

[16] Zhang H, Seward CH, Wu Z, Ye H, Feng Y (2016) Genomic insights into the ESBL and MCR-1-producing ST648 *Escherichia coli* with multi-drug resistance. Sci Bull (Beijing) 61: 875–878. 27358749 10.1007/s11434-016-1086-y PMC4899488

[17] Zhang XF, Doi Y, Huang X, Li HY, Zhong LL, et al (2016) Possible transmission of *mcr-1*-harboring *Escherichia coli* between companion animals and human. Emerg Infect Dis 22: 1679–1681. 10.3201/eid2209.160464 27191649 PMC4994340

[18] Poirel L, Kieffer N, Liassine N, Thanh D, Nordmann P (2016) Plasmid-mediated carbapenem and colistin resistance in a clinical isolate of *Escherichia coli* . Lancet Infect Dis 16: 281. 10.1016/S1473-3099(16)00006-2 26774246

[19] Falgenhauer L, Waezsada SE, Yao Y, Imirzalioglu C, Käsbohrer A, et al (2016) Colistin resistance gene *mcr*-*1* in extended-spectrum β-lactamase-producing and carbapenemase-producing Gram-negative bacteria in Germany. Lancet Infect Dis 16: 282–283. 10.1016/S1473-3099(16)00009-8 26774242

[20] Haenni M, Poirel L, Kieffer N, Chatre P, Saras E, et al (2016) Co-occurrence of extended spectrum β-lactamase and MCR-1 encoding genes on plasmids. Lancet Infect Dis 16: 281–282. 10.1016/S1473-3099(16)00007-4 26774244

[21] Haenni M, Metayer V, Gay E, Madec JY (2016) Increasing trends in *mcr-1* prevalence among ESBL-producing *E*. *coli* in French calves despite decreasing exposure to colistin. Antimicrob Agents Chemother 60: 6433–6434. 10.1128/AAC.01147-16 27503658 PMC5038315

[22] Malhotra-Kumar S, Xavier BB, Das AJ, Lammens C, Butaye P, Goossens H (2016) Colistin resistance gene *mcr*-*1* harboured on a multidrug resistant plasmid. Lancet Infect Dis 16: 283–284. 10.1016/S1473-3099(16)00012-8 26774247

[23] Du H, Chen L, Tang YW, Kreiswirth BN (2016) Emergence of the *mcr-1* colistin resistance gene in carbapenem-resistant Enterobacteriaceae. Lancet Infect Dis 16: 287–288. 10.1016/S1473-3099(16)00056-6 26842776

[24] Yao X, Doi Y, Zeng L, Lv L, Liu JH (2016) Carbapenem-resistant and colistin-resistant *Escherichia coli* co-producing NDM-9 and MCR-1. Lancet Infect Dis 16: 288–289. 10.1016/S1473-3099(16)00057-8 26842777

[25] Partridge SR, Ellem JA, Tetu SG, Zong Z, Paulsen IT, et al (2011) Complete sequence of pJIE143, a pir-type plasmid carrying IS*Ecp1*-*bla*CTX-M-15 from an *Escherichia coli* ST131 isolate. Antimicrob Agents Chemother 55: 5933–5935. 10.1128/AAC.00639-11 21911569 PMC3232798

[26] Stokes MO, Abuoun M, Umur S, Wu G, Partridge SR, et al (2013) Complete sequence of pSAM7, an IncX4 plasmid carrying a novel *bla*CTX-M-14b transposition unit isolated from *Escherichia coli* and *Enterobacter cloacae* from cattle. Antimicrob Agents Chemother 57: 4590–4594. 10.1128/AAC.01157-13 23836183 PMC3754312

[27] Li A, Yang Y, Miao M, Chavda KD, Mediavilla JR, et al (2016) Complete sequences of *mcr-1*-harboring plasmids from extended-spectrum-beta-lactamase- and carbapenemase-producing Enterobacteriaceae. Antimicrob Agents Chemother 60: 4351–4354. 10.1128/AAC.00550-16 27090180 PMC4914624

[28] Di Pilato V, Arena F, Tascini C, Cannatelli A, Henrici De Angelis L, et al (2016) MCR-1.2: a new MCR variant encoded by a transferable plasmid from a colistin-resistant KPC carbapenemase-producing *Klebsiella pneumoniae* of sequence type 512. Antimicrob Agents Chemother 60: 5612–5615. 10.1128/AAC.01075-16 27401575 PMC4997870

[29] Yang YQ, Zhang AY, Ma SZ, Kong LH, Li YX, et al (2016) Co-occurrence of *mcr-1* and ESBL on a single plasmid in *Salmonella enterica* . J Antimicrob Chemother 71: 2336–2338. 10.1093/jac/dkw243 27330065

[30] Sun J, Li XP, Yang RS, Fang LX, Huo W, et al (2016) Complete nucleotide sequence of an IncI2 plasmid coharboring *bla*CTX-M-55 and *mcr-1* . Antimicrob Agents Chemother 60: 5014–5017. 10.1128/AAC.00774-16 27216063 PMC4958226

[31] Lv L, Partridge SR, He L, Zeng Z, He D, et al (2013) Genetic characterization of IncI2 plasmids carrying *bla*CTX-M-55 spreading in both pets and food animals in China. Antimicrob Agents Chemother 57: 2824–2827. 10.1128/AAC.02155-12 23478963 PMC3716176

[32] Doerrler WT, Gibbons HS, Raetz CR (2004) MsbA-dependent translocation of lipids across the inner membrane of *Escherichia coli* . J Biol Chem 279: 45102–45109. 10.1074/jbc.M408106200 15304478

[33] Baker D, Sali A (2001) Protein structure prediction and structural genomics. Science 294: 93–96. 10.1126/science.1065659 11588250

[34] Edgar RC (2004) MUSCLE: a multiple sequence alignment method with reduced time and space complexity. BMC Bioinformatics 5: 1–19. 15318951 10.1186/1471-2105-5-113 PMC517706

[35] Sela I, Ashkenazy H, Katoh K, Pupko T (2015) GUIDANCE2: accurate detection of unreliable alignment regions accounting for the uncertainty of multiple parameters. Nucleic Acids Res 43: W7–W14. 10.1093/nar/gkv318 25883146 PMC4489236

[36] Tamura K, Stecher G, Peterson D, Filipski A, Kumar S (2013) MEGA6: Molecular Evolutionary Genetics Analysis Version 6.0. Molecular Biology and Evolution 30: 2725–2729. 10.1093/molbev/mst197 24132122 PMC3840312

[37] He Z, Kisla D, Zhang L, Yuan C, Green-Church KB, et al (2007) Isolation and identification of a *Paenibacillus polymyxa* strain that coproduces a novel lantibiotic and polymyxin. Appl Environ Microbiol 73: 168–178. 10.1128/AEM.02023-06 17071789 PMC1797129

[38] Kim JF, Jeong H, Park SY, Kim SB, Park YK, et al (2010) Genome sequence of the polymyxin-producing plant-probiotic rhizobacterium *Paenibacillus polymyxa* E681. J Bacteriol 192: 6103–6104. 10.1128/JB.00983-10 20851896 PMC2976442

[39] Shaheen M, Li J, Ross AC, Vederas JC, Jensen SE (2011) *Paenibacillus polymyxa* PKB1 produces variants of polymyxin B-type antibiotics. Chem Biol 18: 1640–1648. 10.1016/j.chembiol.2011.09.017 22195566

[40] Gadler P, Faber K (2007) New enzymes for biotransformations: microbial alkyl sulfatases displaying stereo-and enantioselectivity. Trends in Biotechnol 25: 83–88. 10.1016/j.tibtech.2006.11.006 17150269

[41] Hanson SR, Best MD, Wong CH (2004) Sulfatases: structure, mechanism, biological activity, inhibition, and synthetic utility. Angewandte Chemie International Edition 43: 5736–5763. 15493058 10.1002/anie.200300632

[42] Converse SE, Mougous JD, Leavell MD, Leary JA, Bertozzi CR, et al (2003) MmpL8 is required for sulfolipid-1 biosynthesis and *Mycobacterium tuberculosis* virulence. Proc Natl Acad Sci U S A 100: 6121–6126. 10.1073/pnas.1030024100 12724526 PMC156336

[43] Petrillo Mauro A-L A, Kreysa Joachim (2016) Possible genetic events producing colistin resistance gene *mcr-1* . Lancet Infect Dis 16: 280 10.1016/S1473-3099(16)00005-0 26774240

[44] Delgado-Blas JF, Ovejero CM, Abadia Patino L, Gonzalez-Zorn B (2016) Coexistence of *mcr-1* and *bla*NDM-1 in *Escherichia coli* from Venezuela. Antimicrob Agents Chemother 60: 6356–6358. 10.1128/AAC.01319-16 27431212 PMC5038285

[45] Li Z, Tan C, Lin J, Feng Y (2016) Diversified variants of the *mcr-1*-carrying plasmid reservoir in the swine lung microbiota. Sci China Life Sci, 59: 971–973. 10.1007/s11427-016-5111-9 27520829

[46] Nation RL, Li J, Cars O, Couet W, Dudley MN, et al (2015) Framework for optimisation of the clinical use of colistin and polymyxin B: the Prato polymyxin consensus. Lancet Infect Dis 15: 225–234. 10.1016/S1473-3099(14)70850-3 25459221

[47] Feng Y, Napier BA, Manandhar M, Henke SK, Weiss DS, et al (2014) A *Francisella* virulence factor catalyses an essential reaction of biotin synthesis. Mol Microbiol 91: 300–314. 10.1111/mmi.12460 24313380 PMC3933004

[48] Feng Y, Cronan JE (2011) The *Vibrio cholerae* fatty acid regulatory protein, FadR, represses transcription of *plsB*, the gene encoding the first enzyme of membrane phospholipid biosynthesis. Mol Microbiol 81: 1020–1033. 10.1111/j.1365-2958.2011.07748.x 21771112 PMC3204382

[49] Darling AC, Mau B, Blattner FR, Perna NT (2004) Mauve: multiple alignment of conserved genomic sequence with rearrangements. Genome Res 14: 1394–1403. 10.1101/gr.2289704 15231754 PMC442156

