# Supplementary material for: Dissemination and Mechanism for the MCR-1 Colistin Resistance
Source: PLoS Pathog. 2016 Nov 28;12(11):e1005957. doi: 10.1371/journal.ppat.1005957 (PMC5125707; doi:10.1371/journal.ppat.1005957)
Supplement: S2 Text — (DOCX) [file ppat.1005957.s002.docx]

>pE15017

CTAGATAATACTGTTTGTGTGGTTTATTCGATCGTGTGAATGGGTGTATGCAGTCAAAAAAACTTTTTTGAATATACAAAATTCTGAATTTCTGATTTTGTAAGGTTTTGTATGGGGAACGTCAGTAAAGAAGGGGCTTCAGTAGCTGTTCTGAAAACGATTTTGAAGCAGTCACAGATGTGTGCTGAATCAAAAAGGAAACGAGCCAGGCTTGAACATCGTTGATCGATTGCGCCCATGCTGCTAAATTTGCAGGCAAGTTTTCCGCGATTTGTAAATGCAGAAAACGAGGTGCTGAAATTTTGTTCGAGAACGTAAATTTCAGATAAAGAAAACCCCCGTAATCTCGTCTTTTCCAGGGAGCGCAGATTAGCGGGGGTTGACCTCAAGGCAGAGCCTTGAGCTGATAACATTTTATTTCAGGTAATCGCTTACTACCTGAAGTGGAGTTTAATTCCATGAGAAGGAAATATCAACTCTTACAGTGCAAGCTAAGTGCAGCTTGCTGTGATTAGCAAACGTCACTGGTCTGAACTATCACCGGAAGAGCAGGTCCGTTTCTGGCAGGACTACGAAGCGGGTATTGAAAGCTCATTCCTGGTCCCCCAGGAAAACAAAGGCGGAACAACAAAACGTCGCCGGGGTGAACATTCCACTAAGCCAAAGTGTGAGAATCCAGCCTGGTTCCGTCCCGACAACTATAAGGCGCTGGGCGGACAGTTGGGCCACGCTTACAATCGTCTCGTTAAAAAAGACCCGGTTACTGGTCAGAACACACTCAGAATGCACATGTCGTTGCATCCATTTTATGTACTGAAACGTCAGAGTGTCGGTCGCAAATATAAATTCCGACCGGAGAAACAGCGGCTGCTTGACGCAATATGGGTTGTACTTGTGAGTTTTTGTGATCGTGGATTGCACACTGTCGGCATGTCTGTCTCCCGCCTGGCAGAAGAAATAAGCCCAAAAGACAGTAAGGGAGACGTGATTCCTGAAACCGCAGTCACAGTATCGCGCCTTTCCCGTCTGCTGGCAGAGCAGGTTTCCTTTGGCACGTTGGGAACGTCAGAAAAGACGATATGGGACCGTGAATCCCGCCAGCGACTGCCGAAATACGTCTGGATCACCGAAACTGGCTGGAAAATGCTGGGAGTTGATCTGGTGAAACTTCAGGAACAGCAACGTAAACGCCTTGCAGAAAGTGAAATCCGTCTGCAATTGATTAAAGAAGGCGTTATCCGCGAGGGCGAAGAAATCTCTGTTCACTCGGCTCGTAAACGCTGGTATGCGCAGCGTTCTCTCGATGCCATCAAGTCCCGACGTAAGAAAGCAGCGGAGCGCAAACGTGCTAATCGCCTGGCAAAACTGCCGTATGATGAACAGCGAAACGAAATTGCGCGGTTTATTCTGAAGCGTATGCCGCCGGACGAAGCGTACTGGTGCACTAAAGAAAGATTAGAGCAACTGGTAGCCAGAGATCTACGTCAGCTTGAACTGGCCCTGACAGCTTCGCCACCCCACTAGTTTAGCTGCCTGATACAATCGGCACTCTCTTCAGCCCCTTCGGGGCTTTTCGTGTTGCCTGCATTTCATGAAATTCCTCATATTTGACGACCGTTTTTCATTTTGTCCCGCTCTGACAGTTTTTTGATGGCCGCAATAGGGTATCGGAGTAAAAACGCCTTCGGTTATCCACAAAAACCCGCAAAGGAATAGCTTCAGAAAGCAGCAAACATAGAACATCAAACCCGCAATATATTCTTAACCCCTGTTCTTTAATCCCCTGCGTTGCTTCGCCGCAGGGAAAATATTTATCTTTGAAACAACTGTGGATAATTACAAAAATGCCTTCGCTTGCAGCGGCTAACGCCGCGCCGCTCAGAATATAAAAGTACCTCCCACCGCTTCGCGGCGGGCATATGGACTTCTGCCTAATGAAAAACAAAGCCCCTCCAACCCTCTTACAAAAAAACGCTGCAAATACTCAGCCACAGCGCCCAACCCATCACTGAAAAGCGCCGCCGCCCCCGCCCGAAGGGCGGGAACAACATCGCTTTCAATAATGGATGTTGTAACTAAGAAATTACATGGCTGTCAGTCTTCTGGCTGGAAGTACCAAGTACACGCTCGTAAGCGGTCCTGGCGGCCCGCTAACGCGGAGATACGCCCCGACTGCGGGTAAACCCTTGTCGGGACCACTCCGACCGCGCACAAAAGCTATTTCATGGCTGAAAGCGGGTATGGCTTAGCAGGATGGGGATAGGTAAGGTGAAACCTATCAATCAGTACCGGCTTACGCCGGGCTTCGGCGGTTTTACTCCAGTATCATATGTAACAACGGAGTGCCGCCTTCCATGCCGCTGGCGCGGCATCAAAAAAGAAAGCCCCCCAATGACGGGGGAAATAGCGTTTTGATCAAAATAATAATGCTAGTGTGCTTCTTGCGTGCCATTCCAGGCACAAACTAAATTTAACGTTTCGTTAACTACAAAGAAATCCGATGAGTTAATCACTTGCGAAATAAAAATCATATAGTAAATTACTATATGATTTTGCGTTGTTTCGGAGAATTAAAAAATGCCTTTGATTGAATACATCAACAAGTTCTATCGGGGGAATCAGGCATCGTTCGCCAGGCTGACCGGAGTTCAACCTGCGCAGGTAACACAGTGGATCAACAAAGGATTCATCGTGGTGAATCACACACTGTATAGTCCACGCCGAAAGCTGGGAATTTAATTCCTGGTCAATATTTGACATCCTCCACGCCCTGAAGGACGGGGATATAAGGCGCACTGTATCCTGTTGTCTGGCAGAAAAAAGAAAGCCCCGTAGTTAATTTTTCATTAACCCACGAGGCGATCCCAATACTTCAACAACACCAGGATAGACTTTTACCGCCCTTTACGCAATACCATGTATTTTCAAGTTTCTGGTCGATATATAATGTTGTTGGCGCTTGAGGCTTTCTGCCTCATGGCGAAAAGGTGGTTTGTATCTTGTTGTGCGGCAGAAAGAAGAAAGCCCCGTAGTTAATTTTTCATTAACCCACGAGGCGATCCCAATGCTTGAACAACATCAGGATAGCCTCTTACCGCGCGTTACGCAAGGAGAAGAAGGCCATGAAACTACCGGGAAACGCCCTTATCTGGTGCGTATTAATCGTGTGCTGCACGTTGTTAATATTCACACTCCTGACCCGGAATCGCCTGTGCGAAATCCGGCTGAAGGACGGATACAGGGAGGTTACGGCAAGCATGGCTTACGAATCCGGCGGTAAGTAGCAACCCGGAGGCGGGCGAAAGCCCGCCTTTTTGGGGATGATGTGGTTCTGGCATTAAGCGCCTTTACAAAGGGGGTATTCTGTACGGATACCCCCTTTTTCCATGTATTTTCAGGATATTAACTGGTGAATATGGTATAATTAGTCAATATGACAGCAATTATTGAGGCGAGTATGCAGCCCAAAAAAACACCCGTGATTGTGGTCAAAAAACGCCGCGTTCTGGTCATGCCAGAAAATCCTGTGGTGAATGAAAAGCCGCAGGAAGTACAAAAATCAGCAGTTAATGAAAACAAGAAAGTACAGAAGAAAGATGCTGTAGCAGAGAAAACACGTAAAAAGCAGCCTCAGCCCTGGTATTTGAAAAAACAAATCACTTTTCCCCCAAAATATCCGAAAGAATATTTTGAAAAGTGTTTCAATAAAGTTCGTGCTGTTTTTCCTGAACTATGGACAGACGAAAAAAAGAACTTGCCCCTGAAAAGCGGGATTCTCCAGGACGTTGAGAAATACCTGGCGGATAACCCGGATGTGGATCTGACGATTGAAGAGTGGAATTGTGCGGTTCAGGTGATGACGTTCCGGTGGCAATATCTTCAGAATTGTACTGTACCAGGCGCAACACGTTACGACCTTTACGGGAAACCTGCTGGTACAGTAAAAAAAGCACACGCAACTTATGCGCAACTGGTTCTCGATGCCCGTAAGAAAGCCAGCGAGAAGAAACAATTAAAACGTAAGGGATAAAGCCCCGAAGGGCTTTATCATTTCAGCCTGGCTGACTGTAGAATCCAGTGATTTTTTCCGTTTTCGTCCAGGCCGTGTTCGTAAGCTGCAAGAGCCGCTTTCATTATTGCTGTTTTTGTCTGGCCTGTTCGCAGTGCTTCCCGTTCCAGAATATCTTCGTAAACGTCCCCAAGCCGGACATTGGTCACTTTAGTTGCTCCCGATTTTTTGCGGGTAGCTTCATTGATGAATCGGGCTGTGTCTGCATTGGTTTGTTCTGTTTTTTGTCCTGGTATTACCGGAGTTTTCAAGTCAAGAGCCATTATATCACCTTTCGTGTGTTACATTAGCTTTACTGTGTAAAGTTAATGATGTAAAGCTGTCGTATTACATCCATTAGGGATTGATGCCTAACTCTTGCGCCAAAAGCTCTATTTGTGCTTTAGCTGTAGAAAGGCTGGATGCGCGACGTACATCATGAACGCCAGCGCCTTCATTACAGGCCGCTTCAAAAATATCTAAATCTGAAATACGTGTACGAAGTGGCTGTATCCACACCGGGTTCTCCCGGAGTAACTTGTCGAGATCAATCGCTGCTTTTCTGTGGCGTGGTTTTGTCGTGTTAATTCTCGTGAAAAGTACCCAGGGTTGCAGGTCTGGATTGATCTGTTGAGCTGTGCGAACTTTTTCTGTTACATGAGTCAGCGTTTCTGCTTCGAAATCTGAAGACGGCTTAACGAGAGTAAGCAGAATGTCAGCTACAGTTAAGGCACTGCGAAATTCCTTGCTGTCATGCCCTGGACAGTCAATGATCAGCACTTCACATATTTTTTTCAGGCGCTTAATGACTTCTGATACATCGCCATACTCTTCATAAACAGGAACCGGTAATAAGCCATTCTGCTTTCTTTTTTCGTTCCAGCTAAGAATATCATTGTTTTTATCGGTCTTCAGAATGATGACCGTTCGTGAATTATTTACCAGATAAGAAGCCAGGTTTGTTGCTGTGGTGCTTTTTCCTACACCGCCTTTATCTGATGCGACAAGTAATGCTCTCCCCATAGAAACCTCGGATATACAGTGTAAAGGTTTATTTGTCTTTACATTGTATTCATTATAGTTACGTTGTAAAGATGTTTGTGTCTTTACAATGTAATTGTTTTTTGCGCTTTACATTGTGTGTATGTTACAGGGTAACTTTTCAGGATGCGATTTTGCCTGGTGTGTTGTTGCGCCCGTAGCTCGCTGACGCTCACACGCGCAGTACAGCGCCCCGCCCTGCTGCAATGCACCAGGACGGCGTAAACTCGCTGGCGCTCGCGCTAACTGCGGCATTCTGCGGCGCAAATCAGCATGATCATGGCCTGTCAGTCCGTTGGGCTTTGCAGGGTGAAGGGATGGCCCTGTGCATCCTGGTATGTTCGTCAGCGGCTGCGCCGCGCTTCGGGGCGATGTGGCCCTGCTGCTCCCTGGTATTTTCTTTCGCCCCTTCTATCCGCTGACGCGGATTAAGAAACGAGCAAATCATCAAGTAAATTGCTGTTTTCAGGAAATAAATCTTGATAATACATCTAAATGGATGTATTATTGTTTCATCGAAAGGGGATTGGCTCCTTTCCTTCTTAAGTCCAAACGGACAGGAGATTTAAAATGAATATCCAGGAAGCATTAAACGTTTTTGGATTATCCGGCGAATTAACTGAAAAAGATATCAAAGCAGCATACAGAAAAGCCGCTTTAAAATATCATCCAGATCGTAACCCGTTAGGGGCTGAACTGATGAAAGCGGTAAATGCAGCTTTTGATGTCTTGATGGCAAATATTGATAAAATAAATCAGTTCCAGAGCGCTGATGAACATGCACGATATAATTACGGTGATGACCTGGAAAAAGTATTAAACGTTCTTTCTGGTTTATCTGGTCTGGTATTTGAAGTGATAGGTAACTGGGTATGGATTAGTGGAGAAACTATTACACATAAGGAAACTTTAAAAGAAATCGGGTGTAAATGGGCGGCAAAGAAAAAACAATGGTTTTATCGTCCAGACGAACATAAAAGTTACTGGAATCGTGAAGAACACACGATAGAAGAAATCCGCGCAAAATACGGTACAACCGGACAGCGCAGGGCGACAGGGTGGCAACGCGTGGAAACCAGAGCGTAACCAGAACGGGGGCGAGAAGCCCCCGATATCATACCAGCCCGTCTTTTGGGGCATCGAACCCAAAAAGACGGGAACAGCAGCCACGTAGCAAAAAGGGAAACAGATGAACTCTTTTTTTGAACAGTATCACCCTGTCTTTGAAGTTGTCTGCCGTATCCTGGGGAACGGCTGGCGCGTGAACAAACTTGATGATTGTCCGTCTCGTATAAAACTGACGTCACCGCAGTTTAAAAATTACTCTGTGCATATTCGGATGGAAAAAGACCGATTTTCTGTTGTGGGGAGTGTGGATAGTCGTTCCTGGAGTAGTCCGTATCATGTTTGTACGTTATCCAGGAAGCGGAATCCCGTTGATATAGCAGCCAATATCGAGCGAAAAATTTTGCTAAATGCTTCGCAGGAGGTGTTACAGGCGATTGAGTATGAGAAACGCCAGGCGGCAAAAAAGGACGAAATTCTGATCCTGAAAGGTATGTTATCGCAACTTGTCCAGCTTGAAAGCTGGTATGGGGCATTGACAGGCTTTAAAGCTGAAAATGGATTGAACGGTAAAGTCACCGAGCAGGGCGAGCGTTATGATTTGCAGATTAGGGGTTTGAGTATAGATCAACTTGTTAAAATTACAGGATATTTGAAACAGTTATGAGAAAAAATAAAGGGCGATTGACTTATTATCTTGAAGTGATTGATAAAAAATACCATTTTGTAAAAAAAATAAGCAGTTATTCAAAGGAGTTCACTGACGGAAAAACAAAAAGAACAAAGAGAACGTTAAGTGAGCTGGTTTTTAATGAAAGTGAGGTCGAGGCAATAGACTTTACAAAAAATGGTTTAAGACCTGTTGATAAGAATATTCTCTTAACTATGGTGAAAGAATATAAGGAGAGTGATGCATGACTATTGAAAATACACCGGAAAACATAAAGAAATTACGAAAAAAAATTGGCCTTACCCAGACAGAGTGCGGTGAGATTTTTGGCGTAGGCTTAAGTACATGGCAGAAAAAAGAAGCTAAAACCCACAACCAGCTTAATTTATCGAAAGGTGAATTTGAGTACCTGTTATTACTTGCAGGGGAGCACCCTGAATACGTCCTGCATAAAAAAGCTGAATCCCGCTCACCGGAGGAACCGTGAGCGAGAAGAAAACCTGTTTTCGTAGGAGCATACTGATGATTTGAACATGGCGTCCTCCATCATAGATGGGGCGCACCATGCTCCCATCAGGGGCCGGTTCGCCCCTGGTTAGGGATTAAGATCACCTGGGACCTACGTGCGCCCGCACCGACACCCTCACACCTTCGAGCTACTGTTGCCATTAAGGGGTCGTTTCGTGGTGCTGAATTTTGACGATCGGGGTACCGTCACCCATCGGGCGATATTGGGGGAAACCTGTACGGTGCTGGAGATGGCCGCAGGAACCTGGCATGCCGTGCTGTCGCTGGATACCGGTGGCATAATTTTTGAAGTAAAACACGGTGGCTATCAACCCGTGGCTGCCGATGACTATGCGCACTGGGCTCCAGCGGAAGGAGAACCAGGAACCACGGAGCTTATGGCCTGGTATGCGCAAGCGCAGGTGGGCGACAGCACTTTTGCCGTCTAAGGCGATAAACAAAAACGGAATGAGTTTCCCCATTCCGTTTCCGCTATTACAAACCGTCGGTGACGATTTTAGCCGCCGACGCTAATACATCGCGACGGCTTTCTGCCTTAGGTTGAGGCTGGGTGAAGTAAGTGACCAGAATCAGCGGCGCACGATCTTTTGGCCAGATCACCGCGATATCGTTGGTGGTGCCATAGCCACCGCTGCCGGTTTTATCCCCCACAACCCAGGAAGCAGGCAGTCCAGCCTGAATGCTCGCTGCACCGGTGGTATTGCCTTTCATCCATGTCACCAGCTGCGCCCGTTGGCTGTCGCCCAATGCTTTACCCAGCGTCAGATTCCGCAGAGTTTGCGCCATTGCCCGAGGTGAAGTGGTATCACGCGGATCGCCCGGAATGGCGGTGTTTAACGTCGGCTCGGTACGGTCGAGACGGAACGTTTCGTCTCCCAGCTGTCGGGCGAACGCGGTGACGCTAGCCGGGCCGCCAACGTGAGCAATCAGCTTATTCATCGCCACGTTATCGCTGTACTGTAGCGCGGCCGCGCTAAGCTCAGCCAGTGACATCGTCCCATTGACGTGCTTTTCCGCAATCGGATTATAGTTAACAAGGTCAGATTTTTTGATCTCAACTCGCTGATTTAACAGATTCGGTTCGCTTTCACTTTTCTTCAGCACCGCGGCCACGGCCATCACTTTACTGGTGCTGCACATCGCAAAGCGCTCATCAGCACGATAAAGTATTTGCGAATTATCTGCTGTGTTAATCAATGCCACACCCAGTCTGCCTCCCGACTGCCGCTCTAATTCGGCAAGTTTTTGCTGTACGTCCGCCGTTTGCGCATACAGCGGCACACTTCCTAACAACAGCGTGACGGTTGCCGTCGCCATCAGCGTGAACTGGCGCAGTGATTTTTTAACCATGGGATTCCTTATTCTGAAAGAGACGAAATAACAACAACATGAATACCTAAATTCCACGTGTGTTTTTTATTAGCTTCAAAAATCACTATTTCACGAAGAATTTAGACTGCTTCTCACACATTGTAACATTATTTACAACCACCTTTCAATCATTTTTGATAAATCATTGATTTCATCTTTGCTGCAATGATACTTAATAAACTCTGCAAGTTATCCACAGAGCAACACTCAATTTTATTGATGATATTCTTATTATACCAGACATTTTTCATACACTCCCTTGTACGGATAGTTTTCCGACAACTTCATGATTACATATCTTGCGGTTTTGATTATTTTTGCTGCAAGAAATACATACTTCAAACGAAAGGTCTTTATTTGCTGTCTGTATTCTGAAGAGTCCAAGGAATCAAACTTGAACAACAAAAATAGGTTATATGAAAGCATCATCATTTGAAACACGGCTTCATTCGCCCAAAATGACTTTAGCAAGAGATGACCCACCGCCATGTCGTATTTGGCTTCTTTGATATAGTTTTCAGCATTACCACGCTTTTCATAGTATATAACTACTTTTTCAGAAAGCAAGGTAGTATTTGTTACAAAGAAAAAGTAGTCGTATTCGGAACCTTCTAAAAGTGATAATTGTGCTCTTTCTTTTTCTGGTTTCAGTACGCGAGATACGACAAATCTTCTGTCTTTTTCCCATTTAACTAATTTTGTATACAGTTCTGTAGTTTCTCTACCTTCTTCTCCTTTAACGAATACAATTGATGAATTCGTTGCTTGTGAGGTGAGTGTAGAATAACTTTTGGCTTTAATTAAATATTTGCATCCAAGAGATTCTATCGTTTCGATAATTTTTTCATCAAAGTAGCCACTATCCATTCGAAATAAAATTTCTAAATCGTCTGATTTGATGTTAGCAACAATTTCTTTGATCATTTCCGCAGCACCGTTTGCAGTGTAAGTATTGCCACTTCTTACAAATCCGGTAACATATGCTTTTAATTCGTCGCAAAATGCAAATTGGATATTGTAGCATCGGTTTCCCAGTTTCTTAGGATTATATCCTTTTGACGCACCTTCTTGATGACCTTCTACGTTAATTACACTACTATCAATATCAATCGTAATGGATGTCAATTTACTTTTAGTGAGCAGTTTTTTAAAGACTTTAAAATTAATGTCTCTAAACATTTGGGTTGTCTTGAAGTTGAAGTTTCCTAGAAACCGTGACACTGTTTCAGGTTCTTTTACGGAAATATCAAACTCGTTGACGAGGGGATCATTTTGAAGTAGCTTTAGACGTTCTAACTTATCAATGCCAATGAAGTGACCGCAGAGCATGGTCTTTATATGATTCATCTTGATTTTATTTGTTGAGTCATTATCAAATACGAGGTCATTTTCAATAAAATCAAAAATCCCATTGCTTTTTGCATTCTCAAGGAGCAGAAAAAGACCTGCATTTGATGTTAGATTCTTAGCTTTGAAATCAATTTTATTAATCATAATTAGAACCCCTTTTTACTACTTTTCTTACTATTATTTTACCATATATCGAGTCATAAAAGCTGATAATTTAACATATTTTTGAGCACTTTTCTTTCACCCAATGGGTGAAAGCTGAATTTCGAAGGAATGCATATTTATCAAGGCTTTGATTATGCTTTTTGAAGTACTGACGTAGAATCTAGGGAAAAACACCACGACGCAGAAATCTAAGATATACAGTGTAAAGGTTTATTTGTCTTTACATTGTAATTGTTCTTTGTCTTTACACAGTGTGTGTTTGTAACAGGGCGACTTTACATAATTGCGATTTTGCCTGGTGTGTTGTTGCGCCCGTAGCTCGCTGACGCTCACACGCGCAGTACAGCGCCCCGCCCTGCTGCAATGCACCAGGACGGCGTAAACTCGCTGGCGCTCGCGCTAACTGCGGCATTCTGCGGCGCAAATCAGCATGATCATGGCCTGTCAGTCCGTTGGGCTTTGCAGGGTGAAGGGATGGCCCTGTGCATCCTGGTATGTTCGTCAGCGGCTGCGCCGCGCTTCGGGGCGATGTGGCCCCGCTGCTCCCTGGTATTTTCTTTCGCCCCTTCCATGCCCTGTCGGGCATCACATGAATGTAACTATATGTATTTGATAGTGTTTTTTTTTGAAGAATGGAGGGAAAAATACTTGATAATAGGTCAGTTCTGACCTATTATAAACACATCGGGAGGGGATGGTCCCCAACCACCAAAGCCGCTCAGCGGCAGGAGATAAAAATGAAAACATTAACTTTTAATAACGGCACTGTTTCTGTTGGCGATGTGTTTGTATCTTCCTGGGGATATGAGCAAACGAACGTTAATTTCTATCAGGTTATTTCTGTTCATGGCAAAAAAACCGTAACTGTTCAGGAGATTCGCGCTTCGGTTCATCGGACCCATTCAATGAGCGGATATAAGACCCCATTACTCAATGATTTCTGTGGTGAGCCATTAAAACGGCGAGTGCGTGATTATTACAGTACGCCAGCAATTGAAATTGAGTCATTCGAAACAGCATATAAAGCATCACCGGAAGAAAAACACGAGTTCACATCATACTACTAAGAAAATAGTTGCAGGGGGTATCCCCTGCAACCATGTTAAATTGTTAATAGTGAATACAGGGGCTTAATCGCCCCTTTTTTTATCTGGTGCGCAGTAAAACTCCGTCCTTCAAGGCGTGGAGAATGCCAGGCGAATGCAGCAATGTAATGTTGCGCCCGTAGCTCGCTGACGCTCACACGCGCAGTACAGCGCCCCGCCCTGCTGCAATGCACCAGGACGGCGTAAACTCGCTGGCGCTCGCGCTAACTGCGGCATTCTGCGGCGCAAATCAGCATGATCATGGCCTGTCAGTCCGTTGGGCTTTGCAGGGTGAAGGGATGGCCCTGTGCATCCTGGTATGTTCGTCAGCGGCTGCGCCGCGCTTCGGGGCGATGTGGCCCCGCTGCTCCCTGGTATTTTCTTTCGCCCCTTCTATGCCCTGCCGGGCATTGCATGAATGTAAATATTTGTATTTAACGATATTTATTTTAAAGAAAGGAGGAAAAAATACTTGATAATAGGTCAGTTCTGACCTATTATAAACACATCGAGAGGGGATGGTCCCCAATCACCAAAGCCGCTCAGCGGCAGGAGCTTAAAAATGGAAAACCACGCCAAATTTGTTGCTACTGAAATTCTTAATCAGCTTGGTGGAAACCGCTTTATTGCTATGACAGGCGCAAAGAACTTTGCCTGTTTTGATGAAAATGGTGAGTCTGGTCTTTGCTTTCGTTTACCTTCAAATTTTGCAATGAAGGGAATTAATTTGGTTAAAATCAAATTAACCTTCTCTGATACATACCTGGTGACATTTTCCCGTGTCCGTGGAGCTACAGTAAAAGAAATATCAAAATTTGATAATATTTATTGTGATCAGCTTGAATGTTTATTTAATGAACAAACCGGACTTGCAACCAGATTGTAATTTTTAATATGGGGGGGATTCCCCCCATATCAGGAGGATAAATGAAAGTTCTTATTGGTAATATAAATATTGATAATTATCATATGCTGTCTGCATTAGCTGGGATTGCGGGATTTGACAGGAGCATTGAGTTTACATGTGAAATATCAGCAAGTATCGAAATTATGGAGGATGATTTCGTAAATAAAGCTGGTATATTAAAGATGTTAGATGAATTTATTGAAAATGATTTTTCAATAAAGCTCGTCTAGAAAATACTTGATAATAGGTCATTTGTGACCTATTATTAATGCATTGAGAGGGGATGGTCCCCAATCGCCAAAGCCGCTCAGCGGCAGGAGTTAAACATGAAACAATCTACTTTTCCTGCTATCGTCTCTACAACTGGTCATGTGTTTTCTGTTGTGCGCGTGACGCTTTGCACAATTTGCCTTAAACATGAAAAAACAGGCGAGGCTTATGTCGTCATTTTTACAGACTGCCATAATATCCGTGATTATAAAAAAGGCGTTGTTCCGGTTCTTGGGGAGCTTTATCAGGAAGATGTCGATTTGATTACCGGAAAAAGTTAAAAGAAAGGGGCTATATGCCCCTTTACTGTTAAAAGGGGAGACTATCTCAATATGAAGATAAGAAAAGGTGACAGACAATATTATCTGAATAAAGAAGGTGATACGTTTCATCTGGTGAAGCGAGTAAAAACTTTTTCTAAGTCTGCGACTTTAGGAAAAACGAAAGCAACGGTGAAAACTGTTGCCGATCTTGTTTTTCATGAGAAGGCATTCGACACTATCGACTTTGCCAGCGACGGATTAAGAGAGAATGATAAAGAAATTGTTTCTATGATGATTCAGGAAATGAGTGAGGGAAAAAATGCCAAATGAAAACACACCAGAAAACATTAAACGTCTGCGGCAAAAAATTGGCCTGACACAGAAAGAATGTGCCGAAATATTTAGCATGAGTCCGCGTACCTGGCGTCGCAAAGAAGAACCTGTCGGAACTGCAAGCGGTACAGCACTTACACCAGTAGAGTTTAAATTTCTTCTGCTTTTAGCTGGAGAACACCCTGATTATGTACTTTGTAAAAGGAATAAAAAGAATTCTGATTCTGGAAGTAATTAAGGAATATTAAGATGAAAATAGAATTAACGTATGACGAAATCACGGAATAAGCTACAAAAACCATAACTGAATTTATGGCTGGACTTAATAGCCCCAGTTGCATTGAACAATGAGCAATTGCGCCCGTAGCTCGCTGACGCTCACACGCGCAGTACAGCGCCCCGCCCTGCTGCAATGCACCAGGACGGCGTAAACTCGCTGGCGCTCGCGCTAACTGCGGCATTCTGCGGCGCAAATCAGCATGATCATGGCCTGTTGGTCTGTTCTGGCTTTGCAGGGCGAAGGGATGGCCCTGTGCATCCTGGTATGTTCGTCAGCGGCTGCGCCGCGCTTCGGGGCGATGTGGCCCCGCTGCTCCCTGGTATTTTCTTTCGCCCCTTCCATGCCCTGTCGGGCATTACATGAATGTAACTGTCTGTATTTGATGGCGTTTTTTTTGAGGAATGGAGGGAAAAATACTTGATAATAGGTCAGTTCTGACCTATTATAAACACATTGGGAGGGGATGGTCCCCAACCACCAAAGCCGCTCAGCGGCAGGAGCATGAAAATGCATTTCACAACTTTTCTAAAAAAACATTTCGATATAGAAAAAGTCGTTGGTACTTCTGATTCTGGAAATGATACCGAATCTATTTATGTTTATGAAAAAGGAAATGATTGCGAACCGTTGTTTATTCTGCATGAATCCTGGCTAAATGCAGAAATTAAAAAGTGTGGGGTATGGACTATTGGCAATATATATTCCACATTGGAACACGGAAAAGAATATTCTGAACAGGAACTAATAAAAATGATTAAGGAGGGTAAAGTAATCAGCAAATACTAATTTGGGGTTGGTAACAGGCTGTGGGGCAGCCTGTTACCAAATGACAGATGCATCTTTGGAGATTCTCAAATGTCAGTATGTGAAATGATAGAGCTTGCAAACCGCATTGTAAATGGGGAACCTGTAAAAACACCCGCGATAGAAGCATGGATTATTAGCGAAATCATTTGGTGGGCGCATAACCGGGATAATGCTTTAGTTTGAGTCATATATGGGGAGAGTGTCATGCTCTCCCTTTCTTATTGGGTAGCGCGTTGTATTACATAGTTAGCTTTACGTTGTAAAGATTTTATTGTCTTTACAGTGTGGTGCGTTTTCCGGCGGTTGCGCTGCGCTTAGGGGTATGTGGCCTCGCTGCTCCCTGGTATTTTCTTTCACCCTTCTATCTGATAACTTTTCTTGAAAGAGGCTGGGGTTTTATGAAACATTTAAAATATAAAGGATATTTAGGTACAGTTGAGCCGGATTTTGAAAATAATATCCTGCATGGAAAGCTGGCATTTATTCGAGATCTGGTGACTTTTGAGGCAGAAACATTAGCTGACCTGGAACGGGAATTTAAAACATCGGTTGATTTGTATTTACAGTCCTGTGTGGAGGACGGAAAGGAGCCTGACGCGCCCTTTAAAGGTGTGTTTAACGTCAGGCTTGATCCAGAGCTAGGCACTGGAAAAAGAAGTCATTAATCATCGCGCAGGGGCTTAATCGCCCCTTTTTGTTGCTCGCTGGCGTTGGTGTTTTCTGGTATTACGATGTAGTGTGTTGTATTTACAAGTAATGCTTTACGTTGTATTACGATGTATACTCTTTAATTGCTTGCAATCTCTTTTCACGTTCGTTGCTTTGTTCAGCCTTTTTCAGTTCACTGAATATGTACTCTTCATCTTTACCCGTGAGTTTTGAACCGGAGAGCAAGCGATTAACAACTTTACGTCGAATTGTGGGGTAATCTTTGGTTTGAGCGAGTATGGACAATATGCGGTCACTGCTAAGAATCTGGCCTTTCCCTGATTCTACATAATGTAGAAAAAATGCGATTGCTGACACGCCCAAAAGAATTGTAAGGACGGCTACACCATCAAGACGAAACGTTGATATAAAGGAATGCGCGACCACTCCAATAATTGCCAAGCAGAGACATAAATAAATGAAGACGTTTTTGTTGCGTTGTTTTTTTTGTGCGCGATCATGATCATCTATGATTGCCAGTATCTTTTCTGGGGTTGGTTGAGCCATTTTTTTTCCTTATCAGGGACACGGAGTGTTTAATTACATAGCTTTACATTGTAAAGCTATGTTTGTCTTTACGATGTAAATGATGAGATGTGCAGTTAAAAGTGTCAATCAGGTATCATATAGTAAAACACTATATGATTTTCTATGTATCACATGCATAGCGATAAAACTGGATAAATGATTTACACTGTAAAGATTGAACTGTCTTTACAGTGTGTTTCATAAGTCATACGAATGAAATAATTTGCTTTTGTATGTATGCGTGTGTATAATTGTTTTCAGGTTAGGGAGGACATATGAAATCTGCTGACCTGTTGAAAGAACTGATAGCTGCTGGTTGCGAGTTGAAACGGCATAAGGCAAGCAGTCACCAGATATGGTGGTCGCCAATAACAGGAAAAACTTTTCCTGTTCCTCATCCTAAAAAGGACTTGCCGCTAGGTACTGTCAGATCGATCAAAAAAATGGCGGGGATTTAATCCCCGCCGACTTTGGAGGTAGCCATGTTCTTTTCTGTTGGTGTCGAAACACCGAAAGATGATCATACAGCATACGGTATCACCGTTCCGGTTTTTGATTGCTTTGATTTTGGATGTGTTTCTGCTGCTGATTCTCAGGCAGAAATACCCGCAATGGCGCGTGAAGCGATATTGGCAATCGTGGAAGAGATGGTAATAAGCGGTGCTCATTCTGTTGATGATATTCATGATGAAGGGTGTTTGACTTATTCCGCCAACCCAAATTATAACCACTGTGATAGCTGGTTTGTAATTGATGTTGATTTGTCAGAAATTGAGGGCAAACAACAACGTATTAATATTTCATTGCCTGATGTATTGATTCGTCGTATTGATGGATATGTGAGAGAAAGTGGTGGCGTATACAAAGATCGTAGCCATTTTCTGGCTCAGGCGGCACGTCATGAACTTGCATATAAATAAAACTATCCCCGCGTTGTGCGGGGATTTTTTTTGATAGCTAACCGCTCGCCGCAGTCCAGCGACCGAGCATAGCGAGCGAGCGACGAGGAAGCGGAAGATCCACCGTTGCCAGGAGAAAAGCAGGTACGCACTATGCGTTCTGCCCTAGTGATGCTTTCACAAACCACGCAGCCTGAACTATTACGCCATGAATCCAGCGCAAATCGGCATCACGATTTTCTTTTTTCATTTTTTCGCCACTTAAAGCCTTGCTTACGTGACTTTTTACCAGATTTTCGTGTAATTCTTTTGCTTCTTCAATTGTGAAACCACCAGGCAAATTTGGCGCTTCTGCTTCCAGCGATGCCAGAGAAATTTCATAAGCCCGACGCTCAATATTGTCTCGCACGTCCAGGCTGCCGATTCGCTCCCTGATTTCTTTAATTAGTTTCTTGTCGTCGTCGGTAAAAGTTGTCATTAGTGACTCCTTTTCATATCTATGGTTTTCTTGAATCAATTTCGTCTAGTTTGAGTTTTATATTTTGCTGGTCTGATTTTGGTAATTTTCTCATGCTCTCTTCAGTTATTTTTTTCATATCAAGAAAAGCTCTTGCATAGAAGCGCAGGCTTGCCTCTTTTTCATGTTGTGCAACATACCAGGTTGCGCCAGAAAATGCCGCAGACAGGCATAATGTGCACAAGGCACAAATTGCAATGGCTGTTCTTGTACTGAAGGGTTTTACAGCCTTAGATAATGACTTCAACTTTTCTTCATATAATTTGTTGTTTTTTTCCTGGTTGCGTTTAATTCCAGATATGAGTTCAATAATATCATCTGTTAATTTTTCTTTTACCTCAATACTTGTTTCTTTTAGATCGTTTTTTCCCTCTTCTACTTTTTCTTTTATTTCATTATCCAGATCGCCGCACGTCTCAACAATATTCTTGAATCTTTGAATATAGTCATTTTCTACAGATGAAACGACCTGTCTGAAGCCCTCAGACAGGGCTGTGAAGTTAGCTTGTAACTCATTGCTGAATTTTTCCTGTATCTTTTGGTTCAGAAAAATTAATGAAATAACTGGATCATCTTTTGATATTTTATGCCCTGTCTCCTTGAATACATCAACAATAAAATTATCAATATCTTCACTGGAAACAGCCTTTTCAGGCTGTTTTAATTCTGGCTGATTAACTGTACTCATATTATTCACTGTGTTCATCGTCATAGATGCTATCTAATTGTTTGTAAATGTCATTGAAGACTCGTTTTAATCGTGATTTAGCCATTAATCCAAAATGGTCGGACTCAAGAGCTTCTTTCAGTGTTAGTGATTTTTCTGTTAGCTCTTTGATGTCAGAGGCAAAGGCATCACTTTTTCTGTCCTGAATAACCACAACGCCAGCGATTACATCCTTGTTTTTTTCAATGAATTTAGTTTCTATTAATGGGATATTTTCAAGTGCTGGAATACCCTGAAATTCATTTATCCAGACAATAAGTTTTACCTTACTTCCTTTAACCAGTTCTTTCAGTTCTTCAAATCCCTGCAAGGTATCCGCAAGAGCTTGTCCACCAACGATAACAGTATGGATATAAACGTCTTGTTCTACATCTTCAAACATGTCCATAACACAGTTATCATTGAAATACTGAATTAACGGTAAAAATGTAGATGCGCCATTGTCAATAACAAAAGTATTCTGACTATTGGTCAGCATTGATTCGAACATTGGATCGAATTTGGATTGTATTACTTTGCTGTTTTCAGTGATTTGTATCAAATCTGCATTTAATCTTTTTACTTTAACCGTAGTTGTATTAACCGGGTCTGTGTCACCGACTACGATATTATCCATGTGATTTTCATCAATAAAGTATTGTGCCAGTATTGCGGTCGCGAATGATTTTCCAACACCACCTTTACCCTGAAGGATGAAATTGATTGAGTTTTTCATTTATAATGCTCCTTTACTTAAAGATATTTATTTATGAATTTTTCATTCGTAGCACTTGGATCGTGCTCTATTTCTTTTTTGTTCTCACTAATGTTTGAAAAAAAACCTTTTACCCCCCCTCTCGGTTCATCGCCTTTAGCTTCATTCTGATCAATTACATCTATGGTTTTTGCCTGTTCTTTTGGTTCAGTGCATTTTTGTCTTTTTTTAGCCCTCTCTGTCATATTTATATAAGTCTTTTGGGCTAGTTCTGTCTTGGTGTTGTTGTTTATGTAATCAGCTATTTGCTCCCATGTATAATATTTCTTTGCTTCAAGTATATTTTGCAAATACATATTGAAGACAGCCTGCTTTGATTTCGATTTTGTTTCGTTTATAGCCTCGCATATTGGAGATAATAAACTCTCGATCTTTTTCATTTGTTACATTTCCTCTCTCTCTCTACCTCCTACATTGGCATAATACCTTATTTAAAGCAATTTGCTCGCCGTTTGTGTGGGTGATTATGTGGGTTGTTTTGTGGGTTGTCAATGGGGGTGTGGTTTTTTTGTGGGTTAAAGTGTGGGTGATTGTGTGGGTTGTGTGTTTTTGGTTGTGGGTTATTATGTGAGTCATTGTGTGGGCTGTATGCACGGCATTTTTTGTTCTTCTAAAACACAGGCACGATACCGATTGTACACAAGACCGTTCCGGTCATGTTACAATCGTGTGCCAAAGGGGGACGCCCCTTTGAAACCCCGAACGATGCAGCCGTTCTTATGCGGTTCTACGAACCTTGAGGACATATGAAGAAAAAAACCAACAAAAACGTTCATGTAACATTCAGACTTACCGAAGAAGAATATGCTCCGTTCGATAGGGCTATAAAAGAGCTTAATATTAGTAAGTCTGAATTTTTCAGGCTGCTTACTATTGGTAAAATAAACACATATGCATCTGATAAACGTAACATACCAGAATACAAACGTTGTCTTTCTCAGTTGAGTTGGGCAGGAAACAACATAAATCAAATAGCGCACCGATTAAATTCAGATCATTTAAAAGGTATTATATCAGAATCGCTTTATAAAAAGGTTTTAAATGGACTAATCGGTATTCGTGATCGCCTTCAGGAGATAGCTAAATGATTGTCAGATACGGTGGTGGTAATGATGGTATCGTTGATTATCTGATAAATGGTCGCAAAGCAGAACGCCAGTACACGCGTGATGAACTTGATCATCGTGTTGTTCTTGATGGTGATTTACAGACTACAGATAAGATTATTGATTCTATTGAAAACAAAAGTCAGGAACGTTATTTGCACATTACTCTGTCTTTTCATGAAAGTCATGTGTCGAATGAAGTGTTAAAGGCTGTTGTTGATGATTATAAAAAATTATTGATGAATGCTTATCATCCTGATGAATACTCTTTTTATGCGGAAGCTCATTTGCCTAAAATTCGTCATATCCAGGATAATAGCACTGGTGAACTTGTTGAAAGAAAACCACATATTCATATTGTAATTCCTAAAGTAAATTTGATTACTGAAAAGTTCCTGAATCCAGTGGGAGATGTTACCAAAGGGCATACAATTGAACAGCTTGATGCTATACAGGAATTTATTAATAATAAATATAATCTTGATAGCCCTAAAGACTATCCGCGTAAAGATGCGGATTACGGAAAAATTATAAGTAGAGTTAAGGGGGATCTTTACAAAGAGCATCACTCTGAATTGAAAGGTGAGTTGCTTTCGCGTATAGAGAACGAGAAAATTGAAAATTACTCTGTATTCAAAGATATTGTTGCAGAGTATGGTGAACTTCGTATAAGAAATGCTGGTAAAACCAATGAGTATCTGGCTGTAAAATTGCCTGGAGATAAAAAATACATAAACTTAAAAAGTCCTCTTTTTCGTCAGAATTACATTGAGACAAGGACATTAACACTTGAGAAACCAACACACAAAGAGATAGAAAAGAGACTTAATACCTGGTTAAATAAAACAAGCCAAGAGATTAAGCATATTTTTAACCAGGCGGAGAAAACAAGGGAACTTTACAAGACATTAAGCCCTTCACAACAAATTGATTTCCTGCAAGAGAGGATAAAAGAATATGACTCAAGAGAAAAACTTAACGAAAGAAATTCTCAGCAAACGTCAGGACGAGCGGGAGGTTACAAGTCGTGTCCTAAAAAGTTTGCCAGAATCCGTCAATCTGAAGCAACAGTCGGATTGTCACGTATGCCCCAACGCGGTATGGTTTACGGAATCAATGGATTCACAAGACCCGACTCTGTCAGTGTATTGTCAGATATTTCGCAGCGTGATCTGGCAGAGCAATTATCGCAAAGAGAACATCCTGGTCAGGATGTGCGACGGGATTATGATAGACAATTCACAGAGTCAGGAATAAAAAGTCTTGAACGCTCTTCTTTTTTGTGTGAAACGATGTTTCAGACTCTGAATGAGGCGGCAGAGAAAAACGAAATTGCAACGATGGCAGAAATTCGCAGAAATATTGATCCTGTCAGGTTTCTGTCTTCTGCCGCCGAGCGTTTTAATATTATTCCAGCGCAGCATAAAATCAGAACAGCAAAGGACGGTTCCCCCCGGTTTTCTGTGGGGAATCGTAATATGAACGCATCTGATTTTCTGACAAAGCATATTAATCTTGCCTGGAAAGATGCGAAATCCTTTCTGCTTGAAGTTTATTCACAGCAGTTAGAAAACACGCCATATACGCGCTATCCAACCTACAGACGCCTTACGCATCATGAAGCCCGTGAGCGTCTTAACTCACTGAATTTATCGGAAAAAACATTACGAAATACTATCAGGTTTGAGCGTGGCAAGCTGTATAATGATCTGCGAGAGATGAGACGCGAGTTGAAGTTAATACCACGTGAACAGCGTGATATTGCCGTGGGTGTGATTGTTTACAAAAAACTAACCACACTTGAGCGTCTTTCTGAACTCGACACAGAGGGGCGACACATAATTCGTCAATATCATGCTGACTGGCATAAGGATAAAGATGAAATGAAAGCCCTTGAACGTCTCAAAAGCTATCTCAACTTCGATGAAATCAACGCCATTTCTGCTGACGAACCCGAACTTTCGCTTCAGAAAGCGGTGGATTCCCAGCGCCGCTTAGAAGAGGCGAAAAAGGTTAACTCCAAACTAAAAGATCTTGTGATGGATAAGCAGGATTCCAGGATTGTTTATCGCGATCAGGAATCGGAAAAGCCTGTCTTTACCGACAAAGGGAACTTTGTCGTTGCGGGTAAAAATCCTTCAAAAGAAGAGATCGGAATAATGCTTGAGTATTCCAGGGAAAAGTTTGGTGGTGTACTCAAACTTACCGGCTCTGAAGATTTCAAAAAAATGTGCGCTGAAGTTGCCGCAGAGCAGGACATGAAGATTATTTTACGCCCGGAGCAGTATCAACAGATGATGCTGGAATTAAAAGCAGAACTTCAGGGTAATAAGTTTGAGCAGGTGGAAACACAGGAAAATAGCCAGGAGTCCGAATCCAGAATAGAGAAAGGTGATGCATTGAAGGAACAGGCCACCGAACAGGAACAGGCCACAGAGCAGGCACAGGCCACAGAGCAAGCACAGGTCGCAGCGCAGGCCACATCTTCATATGATCCTGGTGTTATTACCAGGGCAAACACCCTGGATTCACAGATGCTTAGTAAAGGCACAAATGGTGAATTTGGCTATTTGAAATCTCTGGATAGCGATGAAAATGAGATTTGGGAAGTGCTGGGACATGTACCTGGAGACAGCGATGATATTTTTGACGTTGCCAGTTTTGATAATGAAAACGACGCAAAAGAATTTTGCAAAATTGTGAATGAGTTAGGTATTGACAGAACACAGGCTCTGATTCAGGAGCAGCTTACTACTCAACACGATCAGGCTACTGCGCAGGTGCATAATAAACAGGAAATTTATTGTATAAACTTTAGTCGCTTCCATGATTTGAATGAAGGTATCGTCTTTCATTCTAAAGATGCGGCTATTCAGTGTTATGAGGAAAGCAAATCTTCTGCAATAGAAAAATATGAAAGCAATTATTTAAATGGTGATGGTTTTGATACTGTTGTGCTTATGTCTAAAACTGTCTCAACCGATGAATTATCCTCTTATCCAGAGGGGGCATTAGTAACGTTTGATAGGCCATTTGAAATAATTGCCAATTCATATGAAGAATACCGTACTCCAGTGTATGCGGTTTCTTTTTCGAAGGATGAGTTTAGTGAAGATATTAAGACTTTTGAGTCATTGCACGACGCATCTGAATATAAAAATAAGATGCTTCAGGAGCACGGGCTTAACCAGGATGATATCTTAATCACACCTGTAACAAGAGAAGAAATCGCCTTTAAAGGAATTAAGGACGCTGTTAATGACGCTAATATGGCAGTGATGGAACAGGCGGGTGATTCGTCCAGAGAATCACCAGAAGAGATACTCGCCAGTATTTCAGCTAACGAGCATATGATCTCCGGGCTTGAAAACTTCCTGGTTAAAGACCGTTCTCAGTTCAGTTCCTGCAATGGGGATATCGTTGTGGAGGCGGAAATTACCAGGGGGGAAGGTGGTCTTTATCACCTTGCGGTCGCTGGTAAGCATGGCTTAGAGCGTGGTGATGCAGTTGCCAGAGTGGATGTTACGGAGCAGCAATTTGCCGCGATCACTGGAAAAACACCTTCTGAAGTTTTAACGGGGGACCAAACATCGGCTCGTGTTCCGGTAATTACAGGTATCCATTTCAGTACAAGGGCTATTGAAAATGTGAATAAACTGGAACAACAGAAGGATTATGTCTATTTCTCAACACATGAAGGCCTGAATGCTGAAATAAAAGATTTCAGCTCTCTCAAGGATGCTATTGAGTGGGGCCGGGTTGAATGCGAGCTACATGATCTCAATAAGCGCGATACTGTCATTTATCGCGTCGAGTCTGAACATATTTCTCAGGGGATTGACGCTGTAATGAAAAATGCTGAACGCGTGGAGCGTCATGAAATTGAGAAAGCACAGGGACGGGATTGCACGCCGGAAGACGGCAAGATTCTTGAGGCAATTGATCGCTTTGAGGACAAGTTCAGAGGGGAAGGACTGAAGTTCGAAAGGGAGAAGGCCGAATCCGATTTGCTTAATCACGGATTCACTCGTGAAATGGCAGAAGATGCTCTTGGAAAACAATTTGTCCAGGCCAGAGAAGAACATCTGGAATTGCAGCAACAGCGTGACAATTCACAGGACATGCACTAATTAAGAAAAAAGCCCGGTTTTCGGGCTTTTTAAGATACAAATTATAAATACTCTCAAGTGTATATTCAGTATGGGATTGCGCAATGATTGCCTAATAAAATTTCTGAAATATTTCTGTATCGCATAATTTTTTATATCAGATAAATTGTACTGGATTTCTTAAAAAATTGCAGTAAAATTGCCGCAATTATCCCACCGTTTATTTTTTGAGTAGTTTCTCATGATGCAGCATACTTCTGTGTGGTACCGACGCTCGGTCAGTCCGTTTGTTCTTGTGGCGAGTGTTGCCGTTTTCTTGACCGCGACCGCCAATCTTACCTTTTTTGATAAAATCAGCCAAACCTATCCCATCGCGGACAATCTCGGCTTTGTGCTGACGATCGCTGTCGTGCTCTTTGGCGCGATGCTACTGATCACCACGCTGTTATCATCGTATCGCTATGTGCTAAAGCCTGTGTTGATTTTGCTATTAATCATGGGCGCGGTGACCAGTTATTTTACTGACACTTATGGCACGGTCTATGATACGACCATGCTCCAAAATGCCCTACAGACCGACCAAGCCGAGACCAAGGATCTATTAAACGCAGCGTTTATCATGCGTATCATTGGTTTGGGTGTGCTACCAAGTTTGCTTGTGGCTTTTGTTAAGGTGGATTATCCGACTTGGGGCAAGGGTTTGATGCGCCGATTGGGCTTGATCGTGGCAAGTCTTGCGCTGATTTTACTGCCTGTGGTGGCGTTCAGCAGTCATTATGCCAGTTTCTTTCGCGTGCATAAGCCGCTGCGTAGCTATGTCAATCCGATCATGCCAATCTACTCGGTGGGTAAGCTTGCCAGTATTGAGTATAAAAAAGCCAGTGCGCCAAAAGATACCATTTATCACGCCAAAGACGCGGTACAAGCAACCAAGCCTGATATGCGTAAGCCACGCCTAGTGGTGTTCGTCGTCGGTGAGACGGCACGCGCCGATCATGTCAGCTTCAATGGCTATGAGCGCGATACTTTCCCACAGCTTGCCAAGATCGATGGCGTGACCAATTTTAGCAATGTCACATCGTGCGGCACATCGACGGCGTATTCTGTGCCGTGTATGTTCAGCTATCTGGGCGCGGATGAGTATGATGTCGATACCGCCAAATACCAAGAAAATGTGCTGGATACGCTGGATCGCTTGGGCGTAAGTATCTTGTGGCGTGATAATAATTCGGACTCAAAAGGCGTGATGGATAAGCTGCCAAAAGCGCAATTTGCCGATTATAAATCCGCGACCAACAACGCCATCTGCAACACCAATCCTTATAACGAATGCCGCGATGTCGGTATGCTCGTTGGCTTAGATGACTTTGTCGCTGCCAATAACGGCAAAGATATGCTGATCATGCTGCACCAAATGGGCAATCACGGGCCTGCGTATTTTAAGCGATATGATGAAAAGTTTGCCAAATTCACGCCAGTGTGTGAAGGTAATGAGCTTGCCAAGTGCGAACATCAGTCCTTGATCAATGCTTATGACAATGCCTTGCTTGCCACCGATGATTTCATCGCTCAAAGTATCCAGTGGCTGCAGACGCACAGCAATGCCTATGATGTCTCAATGCTGTATGTCAGCGATCATGGCGAAAGTCTGGGTGAGAACGGTGTCTATCTACATGGTATGCCAAATGCCTTTGCACCAAAAGAACAGCGCAGTGTGCCTGCATTTTTCTGGACGGATAAGCAAACTGGCATCACGCCAATGGCAACCGATACCGTCCTGACCCATGACGCGATCACGCCGACATTATTAAAGCTGTTTGATGTCACCGCGGACAAAGTCAAAGACCGCACCGCATTCATCCGCTGATTTCTCCCTGTATTTTTTCCAAACCCACCGCACACTCCATTCGTATTATGGGCGGTGGGGTGGGGTTTGTTATGCCGTATTTATCAAATAAACGCCTACTTGCTGAGATGAGTATCGCTCTTGTCATGGCGATCGTTGCCACGCTGACCCTTGAGCACAGTCAGATTGATCTGATGGTCGCTGATTGGTTTTATCTGGGTATGGGGCATTGGATGGTTGCCAAGCAAGCTTTTTTGCCAGATTTGCTACTGTATTCTGGACTAAAAAAGCTGCTGATGGCGATGCTGATCTACTTGCTGGTTGCGACCATTTGCCGTGCTTATCATGAGAAAAAGGGCAATGCTATCACTGCCAAGTGGCTTGTCCCAGTGACAAAATTTCGCGTGCGTGAGCTTGCGTATCTGGTGCTGACTTTGATCCTAGTGCCGACAGTTGTCGCGTCATTGAAGGCATATACTCATGTGGTCTGCCCTGTGCATTTGACGATTTTTGATGGTACGCTGCCGTATTTGCCGATGCTTGATAGTATGCGTAACACCATTCCTGATAAGTGCTTTCCTGCGGCGCATGCCAGTAGCGGATTTGCGCTGTTTGCCTTTGCGTTTGCGCCAAGTTTGCGCCGCCGTCGTGGTGCGATCATCATCGTGGTGATGGCATTGGGCTGGGCGATGGGCTGCTATAAGATGATTATTGGCGATCATTTTTTGAGCCATACGGTGGTGTCGATGATGCTTGCGTGGGCGATGTCGGCAGGGCTTGCGTGGGTGTTTTTTAAGAAGGGTGAACAATTCAAGGAAAAAAAATCAACCCCAAACAGAAATAACAGCTACAAAAATGGACAGTAATAATGCAATAAAAGTAATGTTGAGCCATATGTTGGACGAGTCTTTTAAGACTAATGAGGCTGTTAATGCCATTGTAATGAAACTAATGATAATAATGGCTGCAAATAAATATTGTACAATGTTTTTTATTGTACTTGGTTGCTCCCCAATTCTTTCTCTGTAATTTGCATCAACATTAGATATCCATTGAAGTAATAGAGCAATAAAACAGCCTACTCCGCAAACGGTGAATTGCACAGACGTCGGGTTTGAATCAAACAACGAGATCGCTAAGTTATATAAGTATACCGCGCCTGAAATCACCCCGACAACTAAAATAGAGATGAGTATAATCCGAAGAACTAATGTATTGCTTTCCTTTAAGGAATATATTAAAGCCGCTTTTTCGTATTCTTTCATGATTTAATCCAGTCTATAAGTATAAGAACCAACAAAACTGTCCAGGATTCATTTTCTGTGAGCATATGAAATCCCATCCAGAGTGATGAAACTGGCAGAAACACGCCCACAGCCGGAATTATTAGTCTGGCGGAGAATATCCTTATCAGGAGTATCAATAGTGATAAGAACAGAAAAAGAGTGATCATGTGGAAAAACATTCACATCACCTATAACACTATTAAGATAGCCTGCCAGATATCCATTTCAGACAGGCTATATACAGGGTTCAATTCATTCTATGAAAGGTGTATAGGGATTGCTGATGTTTGTGTCGATCAAAAATCTAATATTAATAGATGAGAACTAATATACATATTTCCAGACGGATGGGGGGATTTTATCGTAGAGTTTATTCATTGTGAGTTCTGCAAGCCTGTGATCGGCGGCGGCATTAAACGACTCAAGCTCAGAAGGAGGGAGGCTGTTTTGGTTTTTTTCAATAATCTTTTCAAGTGTGTCGAGTGTTGAGCAACGTCTTAACTGAAAGAGCCAGTCTTGTTTTGTTTTCATTGTACAATTCCTGAAGAACATCCTTGTCCTATAGTAGCTACAACGATGTTTGATTTCTACTGCTGGATTTATTTCTTACTTTTTTGTGTAAACTCAAATCCCAGTTTCCCGGTTGCTTTGTCAACCAGTTTTACTTTTGCTGAAAATTCTTTGTTTGTTTTTTGACTAATGAAACCTTTCAATTCACTTGTAACTCCTTTTGTTATTAACGTTTCAATTTGTTTATCGGTTAATGATTTGCCGCAGAAATCTTTCCATATCTTAAATTCACATCCTGTACAGGAATAGCTTTTTGGCCTGATAACAATCTGTTTACCACAGGAAGGACAAGGAGAGGACAGGCGTTGTGACTGACCAGACGGCGCGCTGGGGGAAACTTTTATTTCAGCACTGTTCGCATTACTGATCATCGGTATCAGATCATTGTACAGTTCGTCAACGAACTGTTCGATGGTCATTTCTCCGTTTTCAATGAGTGTCTGTTTTTCCGCCCATAATGCCGTCATATCTGGATTAACAGCTATATCCGGCAGAGCATCAATAAGCGCATACCCGGTATCTGTCGGAATAAGTTTTCCTTTTTCCAGGGTGATATAGTTCCGTTTTTTCAGTGTTTCCAATATGGAAGCCCGCGTAGCTGGCGTACCAATACCGCCATGTTCGTCTTTTTTATCCCGATCTTTATCCTTCAGTAACTTTTTAATCACTGGATCAGTGACAAAATCTGCGACACGAACAAGCGCGGCAAGTAAAGTGGCTTCGGTGAATAATGGCGGTGGCGTTGTTTTCTTCTCATTCACAACCACTTCTTTTGTCGTCACTGTTTCTCCTGTGCGAATTTTACAGAGCAGATCAAAAGCGGAATCATCATTTTCGGCTTCTGATTCCTCATCCCCGGTATTTTCCTCACCAAGAAACGCTTCAAATCCGCTGTCTGTTGTTTTCCTGGCACGGGCATAAAACGACTCATCACCACACTGAATGGCAACGGATACTTCCTGGTATGTTTTTTCAGGCATGAACTGAACAAGATAGTGTTGTGCGATTGCCAGGTAAACATTGCGCTCGTCACTGCTCAGCGCGTTTACATCCGGTACGTTAACGGTCGGGATTATCGCCGTATGCGCAGTCACTTTCGCACTGTTAAACGCCTTGCTTTTACGTGTCGAATCAACGTCCAGTGGCTGATCGAATACTGACTTCAGGGCATCGAGAACCTGTGGTGCTTCGCTGAATTGTTCATCAGAAAGATATGAGCAATCTGAACGGTTGTAAGTAATTGCTTTGTATTTTTCACGTAGCTGCTGTGTGATATCCAGCGTTTGTTGTGCCGTCATTTTGAACTTCTTGTTCATGTACTGCTGAAGCCGGACCAGGTTAAAGGGCAATGGCGCAGCCGTTTTTTTATCGTCTGTTGCTGCTGCTTCAACTGTCGCTGGTTTTCCTGCAAGGGAAGCCGCTGCTCCATTCGCCCACGCCTTATCAAGTAATTTACGGTCTGTCAGTGGTGCGAACTCACCAGGTTTCCAGTTCGCCCTGATAACATCAACGCCGCGTTGAAAATGCCCGGTCATGGTGTAGTAGAAACTGGATTTATGATTCTTGTTGGCACGGGTGCGATTTACTATCAGGCCAAGAATAGGCGTCTGAACACGCCCGACCGAAAGTGTCCCTTTATAGCCTTTAGCCCGTGCCGGGATCGTATAAGCACGCGTCATGGATAAACCGTATATCGCGTCGGCTACAGAACGCGCCAGAGCCTTGAGATAGAGTCCTCTGAAGTCACGGTTATTTTTGGGATTTGCCAGTGCCTTTTTCACTGCCGGAAGGGTGTTGTCGTTGATCAGAACGCGCTTAACGGGTTTTGTGTTGCCTGCATATTCCAGGACCTCATCGACAAGTAGCTGTCCTTCGTCATCTGGATCGCCAGCATGAATAATTTCGGTCACATCGGCACGCCTGATAAGCTCAATAATCGTTTTAACTTGTTTTGCGGCGCTTTCTACTGGCTGGTATTTTACGGGATAAAGACGCAAAGGGAGTGTTTCAATCTTCCACTCTTTGTATTCAGGATTATAGTTTTCGGGGGGCTGTGATTCGATAATATGACCAAAACAGTTAGTCACAATAGTATTATCACTTTCAAACCAGCCATCATGACGGGTAAAATTGCCACCAAGCGCCTTAACAATATCATTCGCTACTGCGGGTTTTTCTGCGATAAAAAGTTTCATTTTGTTATCCTTTACATTGAATTAATCTTTTGGCACAACGGATAGCACGTTCTTCTGATACTGCCTGTTGCCAGTTATCTTCACTTACTGGATGTCTGAATCTGCGTTCACAGCCATTAAAAGCAAAAATCCCCTCACGGTTAAGTATATTAGTTGCAATATCCTGGTCTTTAATTCTCAAAAAGGCTGTTACGGCAAAACCAGAATACGAAGAAATTTTTGAAATGATGCATTCATCAGCTTTTATTAACGCGGCAAAAGGAACTATCTTTTGATGACCACACGAAATCAGAGGGAGTGTGTGAATATCATAATAAGCTATTGAATGACAATCCATGCGGGTATCATCAATAGCTGCCAGCCAGAATCCTGTCTGGTCTTTTATTTTTCTGCTAATATCTGAATCTGAAACACCAGGATTTAATAAATATCCACCATAACAGACATGGAAGGGGTGAGCAGATTCATCAATATCAGGAAGTTCTTTTACCGGATTTTCTTTCCAGTCCTTCAGAACACCGAATTCAAGAGCATCCAGCAAAAACTTTCGTTCAAATCCATCAAGCAATGCATATTGCATGTTAAACCTCTCAAAGAAGCTCTGATTTGTTGACCTCATGGTGAACCAGTTCACCATTGCGTAAGACAAACAGGTAAGAAAACTTTCTTTCCTTATCTTTCAGTCAGTTTTTGTATTCAGAAAGAATAAACACCCAGTCATCGGTGCTGTAATGAGATGCGATGACTCATTATCTTTTTAGTGATGAGTTGATTATTTTTAAAATATGCCTGCCTTTAATTAGCATGTTTCTGGAAACAGCCTGATTACGAATACCTCTCATCTGGTCAAACTGATAGGTTAAATCATGTTCTTTTCGTGAAAGTCCAAGAGTGGCTCTTTCATTATCCATCAGGACTGAAATTGCAATATATTCTCCAGATGCACACCAGTCTGCATAACTGGCAACGCAATGTTCCATCTCCTGACCTTCCTGACACAGAGCATCAAATGTAATTAATTCATTTACACCGTTATCAACTTTCTGCCAGTTTATCCCAGTTCCTTTCCATTTTGGGATTACGGGAATAACGTTATTCTTCACTTGTCGCGTCCATTCATCAGATAGTCTCCAGAAAGAAGGCCACTCCTGGTTTTTATGAATTAACCGTTCTTCTGCTAACAACCAGTCAATTGCATGACACAACTGATTCGTTTCCATTTCCCAGCGTGATGTTTGCCTGGATTCAGTATTACGGTCATAAAAGCCAATGTTTTTAAATAAATCACGATGATATTCTAGCCATTTTGAGCATATACGATAAATATCATTTTCTTTTTCTGAGTAAGCTCGAATGCTAATATAATCGATAATCCAGTAAATTGCTCTCACAGGATAGTTCTTGATAAGTGGATGGCGTAATAATCTGACTGTAAATCTGAATGATGCATTATCATGTTTATCATTTAAAGCATATACTAATGACAAACTAACATGTCGTAAACTTCTGAACTCACCATAACTAAGTTTGCAGGGAAAACTGATTTTTTCATATAACTCTTTTGTCTTTCCAGATAGTATAAACTGACCATTTTTATGTTGTTGTCTTAGACGACCAACCATAGGCCACAATCCGAAAAAATCCATATCATTCACAATATGGATTCGCTTTGAAAAATCCAGATAGTCCTCAAATAATGCCTGACGAATTGAATTGTAAGCTATCGAACAATGTGAAGCTCTGGCATATTGTGCAAATATCTTTTTATCTACCATTATCCATATAGCTTTACTCAATGCTTTTGAAGCATTAAATTCATTATCCACCCCTATAATTTTTAATGCTCTCTGATAAATTTTGTTTTTTATCTCTGCGAGTAAAATATGTCTGCTGATATATCGTGTATTATAGGGGTAGTTGAACGATGCTATTTTACCAAGGCAATGAACAATGTCATGTATTCCATACTCATATGAGTAATATACATTGGATGGATATAGTGTGGAAATATAACGACTCTCACTGTTTTCCTCTTCCTCTCCAATGCGTAACAAATAGAGTTTGTCTTCAATTGAATATCCAGCCTCAATCAATGATTTGATGATGTAGCTAATCGCATATTTTAACCAGTCGTCCATGTCGTATGCAGATTTTTTAGACCAGAATAAAAAAATCTTACCAGGAAATGAATTTCTTCTGGTCCATACCCCAGTGTGCTTATCTCTTAGCTTGTATCCCCACAAAGTGATTTTGTTATTCTTTTTGTTTTTTCTAATACGAAAAACGCCACAAATGATAATGGAGATGTACTTTTCATTCTCCTCTGTTTTTACAAGACGAAGCCCCCCTGTATTTTGATAGATTTTAAAAACATCATGCAACGTAAGAATATTATTGTCCCTTGTCTGTTGATTAATTACATACATGCCGCCCCCTGTTAAAAAGAAGCCTTTAATATTGCACAAAGATAAAAAGACAGAATCATGGCAAACCAAAAGAAGAATGCATTTTCTTGCCCCCTTAATTTCTGATACGCCATTCCCACGGTGGAACCGGATTTGCATCGGCCCACAAACCACGTTTTTGTTCCTGCGCTTCCCGTTGCAGAGCCGGTAATGACTCATCCACGTTGTAACGTTCATATACCCATGCAGCGCCAGACTGAACCATAAAGCGGCTGGCATCCGTGCCGTTCGTCGTGAACACATGACCGATAATGCGTCCGTAGCGGTCTTTATGTGAATAAGAGACTGTCACCTGTTTACCTGCAACCAGAGTTTTAAGCTGACTAGTTGACCAGCGTCCAAAAGGTTGTTTCTTTTCAGGGGCATCGATGTTTATCAATCGGACTCGGATCGGAACTTCATACACAACAATTTTTGCGGGTAGTGTTTTAATTTCTATGGTGTCGCCATCGAGAACGCGAATAACTTTCCCCTGAAGAATTTTCTGGTTAAATGTATAAGGTATTCCACCTGCATTTGCAGACTGAAAAGCGGTAAGCAGATAAATAAATATTGTTAATAAGATTTTCATAAAAGATTAACGTTAATTTGTCTGTGCAATGGATAGAGCGTTATTCTTATTTTCTTCCTGCCGCTTCATCGAAAAATGATGATTTTCCTACTGCTGCGGCATCATACTGAAATGAATATCCAAAATCATCGTCATGCTCAAAACCTGTATTTTCTCCATTTTTTAGTCTGGAAAGAATAATCTCAAGTTGACTGATTATATCTTCCCTTTCACTATGACGAGTGCCTTGTAAGAGAATAGTTACTTGTTGCAAAGGATATGCATGTTCAATCCATGCCTGGCTAGGTCCAGAGTGCGTTGTATATAAATTTTTTTGTGCCGAAATATTATTCAGAAAACAGTTTAAGTCATCATTGGAATAAATTTCTTTAATATTGTTGATGTTGGATGTTAGTTTTCCAAGTTGGAATGGTGGAGAATTTTTGTCTTCATTGTTGTCTCCTTTATCGTATTCTGGAAACACAATTAACAAAGGTGCACCATTAGTTCTCTTCATAAAACGGGTTACTAGCCGCCCAATATATTTATTGGACATATGCTGTTCAATGTCCAGGACAGCATATTCCTCCGCTGGGAATATTTTGTTTTTTTCGATGGATTTAATTTTTCTGGTAAGATGAATGTTTCTGATAAGCAAACCTACTATCAGGAAGTAAATAAATATCCAGAATAAAAAATCACTCTGTAGTTGTTCTATCATAAGTGAATCCTCTAATTTGTTAATGGACAAATCATTACCCGTTCATCAATGTTTGTAGGAAGTTCGCCAGGAGTAGGGACGCGTTCTCCTCGCTGGGCGGCTTGTGCCAGTGTTCGTAGCAGCAACACGCTGGCATGAAATATGGCCTGTTCTTTTGTGGCTGCGCTTGTTTCCAGGTCGAAATCACTCAGAGTCACAACGACTTGCCCGTTTCTGTTCTCGACCGTTGCCGGGTAGGGAACAAAATAGGGAGCGATTTTGCTGGTTTGCTTGCGTCGGGCATCGAGTTTACTGACAAGTTGATAAGCCCGAAGGTGTGTGTATCGTTTCAACATGTTCAGGGAGCGATGGCCTGAAATAGCGGCAACCTCAATGACGTTCAATGTACCAAGTTCGAAAAATCGGCTGATAGCCTCATGACGGAGGTCATGAAAATGCAAGTTTTCTATCTTCAGTTCCTGAAGTGCTGTTCGCCAGGCACTTTTGAATCCCGAAGAGGTATAAGAAAAAATATTACCGTTAAGCTGTGTCGGCAGCATTTGCAGGTAATTACGGGCTTTCCTGGATAGCGGAACATCGCGTGGAGCACCGTTTTTGGTTGTTGGAAGATGAGCTACCCCATGTTGCAGATCGACGTGTTCCCAACGCAATGACAAGATTTCACCTTGTCGCATTGCTGTTTCCAGCGCCAGATGAAAAATGACGTAAAGAGCCTGATTTTTTTCTTTGAAATACCGCGAAAGTCGGCGTTCTTCTCCTGAAGTCAGGCGACGATCGCGCCCGCTACTGATTTTGGGTTTTCTGACCAGTTCGACAGGATTCATGCGGCATGTTCCCCATTCCACACGGGCGATATTGAATAATGATGACAACAGGGCAAGTTCAAGACGGACGGTGTTTCCGGTTATTTGACGCCCCGTTCGTGGGTTAATTTGCGCCAACCGTTGATCCCGATAATTGGCAATATCAACAGTGGTAATGTCATCCATATAGCGTTCAGCAATAGGGTGACGTTTAATCACATTTACGCGATAAAACTCCTGTAGATGGCCTTTTTTGTGGATGGATACTGTCTTCAGGTATTTGTCCAGCGCCCGGCTGATTGTCATTTTGCGAATTTTGATTTTCTGAAACATATCCCCTCCAGACAAAAAAGGGGGGAAGGTAGAGATTCTTGCTTCGAAATGCAATATCACCGTGCCAATCCGGTGTGTGGAGGGCATTAGGTGGAAAGCTCAAGGTTACTCAACTTTCCAGCACTGGTTACTTGGGGCAATTTGACTTTTGTGCCATTGCCAGAATGGGAAATGCTGAGGATTCTCATTACTGCCAGGTAGTCGAGAGTCCATCTGGTTCACGAAAATGGTACAAATACGAGCACAAGACAGGTTGTATTGCGTCCTGTGTAACTCTCAATTAAGTTTGGTATCCAAATACTGAAAATACCCCTGAGCCACAAGAGTAGTTTTGGGCCGGATAAGAGGTGATCTGATAAGTTGCGCCAGCAGGGACAGCAAAAGAGATGAATGCTGTCTTCCCATAAGATGGGTTATTACTCGCATTTGTGCTAATCAATGCCCCTGCAACGTATCCCTGCAATCTGGATGTGTTAGCACAATCTCCCCCGGCGGAACCACCATTCCCTCCAGAGGCGTAAACAAACAGCGTACCACTTCCCGTATTGCGACCAGTAAATGATCCTCGGTGTGATCCAAGATTACTGTAACTACCGTTAGAGGAACCGGAAGTTCTCCACACACCGGATTGGCACGGAGGCCAGTGCCAATCCGGTACGTGGAGAAGGGCATCAGGTAGCACTGTTCTGACAGGAAAAATAGCAAATGGACAACAGATCCCATTGCCATCTGGTTTTTCTGCCAGCCAGTGTACCTGGAGTGTCAGTAATGCAGAAAATCCGCACGGGTGGAAGCCGAATTATTTTGCGGGGTCAGTTGCAACCTATGATGCCAATCGAATTGTGAAATGCGGCTTCTATGACGAATACAACTTTTATGGCGGTACTCACCGAACTGACCTGTCGGGGAAATGTAGTTACATCGTGGTTTGCCAGTGATAAGTATCGTCACTGGCAAATGGCGTAAACTGTAATTGAACCATTTTGATCTTCGGTCGTGTCGCAACTGGCAGTCCATGCATTGTTGCCTGAAGGCATGGAACGTGTGAGCCATATAAATCCGGTATTTGATCTGCATTGACCGCCTCCACCAATGACTTTCTTGCCAGATGGACATGTTGCCGTTGCTCCAGGCCAACGCCACGCAGTAGCTGAAGCGGTAACAATTTGGCTATCACCTGCGCCAATTTTTTTCCACGTACCGGATTGGCACGAAAGTATCGCCCCTGTACTGTCGCGGCCTACAAGCCCGTTAGGGGAGCAGGAAGCGCCAGCCGTTGCTGTTTTTTCAAGCTGTAAATACTCGCCTGTATAGAGGCGTCCATCAGCGCGTACAGTTCCACCTTTTACCTGGCCGCCAGTATAAATCCCTTTATTATTCACGCTGCGCAACCAGGACGAATCGGACATATACCAGCCGCCACCATAGGTACTGTTCATCCAGCCTTTGCTGTTTTTTGTGACCAGCCAGCCATTATTCGAAGTGATATCGCCGTTGGTTATGGCTGTATTTCCTGAAATTGCGCCAGAGAACGTACCGTTACGTGCGCTGATATCGCCGGAGAGCGCGGCGGCGTTGCCGTTTAAAGTGCCGACGTTGTTCAGATTGTTGCTGTTCATATCAATCGCCGTATGCATCCTGTTAAGGTCGGGACGATTGGTTATGGAGTAACGATATAAACGGTCGCTTTCCTGCAAATCAGCGCCAACAAGTGCCATTGCAATATGCCCGGTCGCGCAGTTCAGCCCGTAATTTGAAGGCGTGTCAGACCAGCCGCCACCCGCGCCAATCGCCTGCTTGCTGCTGTTGATGTAGCCGCCAAGCCCTTCAATCATGCTTGCTACCGAGCGCATTCCGGCTTCTGACAGGGACTGACCGCCATTACTACAGGTCAGGGCTTCCAGACGGGATGTTTTGCTGTTCTTTGCAATACCGGTAATGTAGCTTTGTCCAAAATTGGATTCAGAAAAACCAGATTTCAGATAGCCTTTCTGAATGAGTGTTGCTGGCGTGATTGTTTTGGGTAAAGAGCCAGCAAGTAGTGTTGATGCATTGTCAGCAACATAATTACGAACAGCGGTGTTATAGGTATTTGCGTGTTCTGCTGCATTCTGCCAGCGAACATTATTCAGATGGTTTTCAATGTTTTCATAAACTTTAGGGATAACCATCACCATTATGCCGATGACAAGCAATACCTCAAGGAGAGATACACCTTTATCTGTCTTTTTCATTGCTCTTAATCCTCAGAATAATATTCCCGGTACGAGTGAGTAAACAAACATTCCGCCACAAAGAAATGGGCCAAATGGGATATATTTTTTTTGCTTGTTTAATCCGCCAAGAGTGAAGAAAATAATTCCGCCGATAACGGCAATAAATACAATTGAACAGGCAATCTCAGGCGTTGGAAACCATACGCCAGTTCCTGCAATAAGTAGCACATCTCCCATGCCGAAACTTTCCGTTCCGTTTTTATATGCAAAGTAAAGTCTTACAAACAGCATAATGAAGAACATCAGTATTGCACAAAACAAACCAGACAATATATTGTTATTCCATGTCTGATATAACAGGCCGGAGAAGATAAATGTCACGGTATAAAGGCGTGGTAGTAGAAATGAAAGAAGGTCTATACAGGCCATTAATGTAAGGAAAGCATAGAAAATAATCACTATTATAGATTGAATCAGTGATAGCTGATTTAATGAAAGATACATAACGGCAAGCGTTGTCAGTATCAGGCAGTAATATTTCTCTGCTCCGGTTTTATAAGATACATTAAGTTTATTTAAACGGAATTTAACTTCATTGTAGGCGTAGCATTGACAGACGATATTTGCACAGCAAAGTATCGCCGTATTAAATATGCTGTTTAGCATAAGTTACCTTACCATGTATTTATTGTAGACATAGCGTGCGTATTTGATTCTTTTCTGTTCGTTACTTTCTTTGAAACCAGCGTTATAACTGCCAAGACAACTCCAGTTTATGCCGCATTTCTGAAAATGTGTTGCCAGAATCCACGCGCCAATCTGTATATTGAGACAGGGGTTATCAATAAGCTCTGATTTGTCTTTTATTACGCCAAGTTTTTTCAGGTTAGGGATGTGTGATGAATTAATTTGCATTAGTCCGTAATCGGTGCTTAATATTTTTCCTTTTTCTTTGTTGTGATTTATCCGGTCTGGTATGAGTGAACTTTCTACAGTGGCAATGCTTTTCAGAAGCAGGGGGTCAATCAGGTAACGTTGCCCGGCTTGTGCAAAGCAGGCATCAAATCGGGTCGTTGTCGCTGAATATGCGGGTTGCAATGAAAAGATTAATGATGAAATACATGATAGCAGGATATAAAGAACAGAACGCACAATACTCCTCCTTAAACATGGAAAAAATAAGCGCCCGAAGGCGCTTATCCGATGCACATGAAAAATCAGGAATCAGTGCTGAAGGTCAGCGTATTGCTGTCAGATGCGCAAACAGTTGCTGCACTGACGGTTGATGTGGAAGTGTTGTTAATTTTGGAGATTGCGCTGGAAGAACGCAGGGCGTTTACCATTGCCATGCAGTTTTTCTGTGGGACATTGGCCTCTACAACATTGAAGGAGTATTTATCCGAAGAGGTGGTGATTGTAACGGAGCCGCCCCACGGGTTTTTCGCGCTTGCGCCAGTTGTATCTGCGATCATATCGGATGGCAGTAATCCTTGCGCGTAGAGAGTCTTAATATAGTTACTGTCAGTATAACGCCCCTGGAATTTCAGTGATTTCATGTTGGCAATCACTGTCAGGACATTGTTTTGTTCATTGGATGATTGAATGTTGGATTGCACCATTGAATAGAGTTTATATGCACTTGCTGCAAGTACAACAATAACACCAACAACCAATAACACTTCCATCAGTGTTGCCCCTTTGTCCTGTTCTTTTTTACGGCGTGCAGAAAGAGAAGAAAAAACAGAACGCATATTTAAAATATTAATAGAAGACATAATAAATAATTCCTTATTTATTGAGAGGTTAAAAATATTAGGGATTAGTGTTGCAGGACGTTACTGACATCGTAGAGAGCAGTAATCATCATTACAATAAATGCGAAAACAATTAATGCGGATACTACAGTTATCCAGACTCCTGTTGATTTGACTTTTCCTTTTGCTTCCTCCAGCCATTTATCAGCATAGTTAGACATCATCAATGCATTGTCAGCATTTTCAGAATGAAGTAATAGTTTGTTAATGCATAATTTCGATGGGAAGTCATAACCGCAACTTTTCATTGCCATTGCAAGTGACTGTCCAAGTAAAACATATCTCCTGATTTCTTTTATTCGTTCGAGTAGCCATCTGTTTTGAGTGCTGTTTTTCTCCAGCCTTTCCAGTGCTTTTGCAACGGGAATATCAATACTTAACATGGAGGCCATGTTGAAGAGAAAACTTACACCATTAAATGTTTTATAAAGTGACCAGGGGGGAATGTAATCAAGATACTGACGGCCCGGTCCTGTATAACGCGCAAGCGAATATCTGATCAGGAAAAACAGTACGATGCAGAAGCAGAGAATATACGTTCCGTTTTCTATCAGGAAACCAGACGTATCTGTCAGTAATCGCATAGAACTTGTCCAGCGTTCTTTCGGTGCGAGAGAAAGGAAAACAGGTGTTATGGTATCGTGGACCATATACATGTTTGCCAGACAACCAAAAACCAGTACGGATGGATAAATCATCGACATAACAAGAGTCATTGTCATTTCAGAACGACTTCTGACAATTTTTATGGCCTGAACTAACCCTCCCGCAAGGTCGCCGCGAATTTCACCGCTGGATATTAATTGATATTCATCCTCCTTTATATATTTTTTTAATGCTGAAGATATTGCCCCTGTGTTATTCAGTGCATTAAGGCACTCACAACAAATATAGTAGGTTGCAGAAGACCCCGTTTTCTTCCCGTAGTCTGTTTCTACATCAATCAGGTGTGTAAGAGCACGCTTTACATTTATGCCATCATTTAACATTCCAGATAAATCTTCATATAAACTTATTCTGGAATACCTGAATGTGTATTTTGCCAGCCAGTGGGATATTCCAGAGAAATCAATTTCTATCGAATTGAGTTTCTCAAATATATCTTTTATCGCCATGTCCCCTCCGGGAGCATTGAGTATTTATCTTCGTCAATGGGGGATATGAAGTGTGCGGACAGCGGATCTACCAGGCCATGATTAATCAGGTGTAATAAGTGCTGATTACGGGTAATTCCTCCAAGTTCATAGTGCCAGTATGATTTTGCTTCCAGACGCCCGGACTGACGATATAACTGAAAAAAACGTGCGTCGGGTGAGACGACTTCTGCTATAACCGTTCTTCCCGTTACGCCTTTATAACAGTGCTCGCAACCGTCAAGATTGCGCAACCGGACCTGTTCCGGCAGGCAATTTTCCTCAATGAGTTTCCGTTCTTCTGTACTCAGTTTGTCTGCCATTTCAGCATAAGTGCGCTTACAGTAAGGACAGAGTTTTTGTACCAGCCGTTGACTCAGCAGACCGATAAACAGAAGAGGATCTGCAATTAGCCTGGCCTGAATATTCTCCATTTCAAGGCGCTCAATAATATTCAGGGCATCGTTGGCGTGTAAGGTCGTCAACAGAAGGTGGCCTGTCATCGAGGCTTTGATTGCTGCTAATGCCGATGCGTGATCACGAATTTCGCCGTTCAGTATCGCGTCTGGATCAAGTCGCATAGCGGATTTAATCGCGTCAACCCAGCCATCGACGGAATCCCTGACTGCCGTCTGTATTGCGCCGGGAATGCGGCCCTCCGGCGGTGATTCAATGGTGAAAAGGCGTTTTCGGGGGAGACGCATATTATCGTTATGGTTGAAACCAAACGTGCTCAGATAGGCTTCTGAAGCCGTTCTCAGAGTCGTTGACTTACCGGACCCTGTAGGGCCGGATAAAACGATAATTCCTTCCGGGCGTTGTAACATTCGCCTGATTGTCTGGATTTGTTGTGGCATGTAGCCAAGTTCTTCGAAAGTGGGAATATCCTCGCTGTCATCTTTAATCAGGCGGAAGACCGCGTACACACCACCCACGCACGGATAATGCGAATAGCGCGCCCCAAAAAGATTTACAGGCTTCAGAAAATTCTCTGCGATACGTGCATCCTGCGGTATTGCCGGGTCAAACTGCGTACCTTTGATAACATCGCTCATTCCAGAATAGGCAGCACCCAGCAATTCCAGCCCTTCTTTACGCGGGATGACATCCAGAATTTCCAGTTCCCCATGCACGCGTGCTTCGACATAAGTGAACTCGGAACCGTCCCGACCAGGTGTGATGTGCAAATCGGAACTTCCCAGGTTGTTTGCTTTCGCCAGGTAGCTAATGACCTTTTCCTGGTTGCTGTTTAAGTGTTGTGATGAGTTTGAATCAGACCTTCTTTCGCTGTCCTGGTAGCGGGATGCGATAACATCAAGAGTGGTCCAGGTTATATTTACCTTTCCGGGATATTTAACATTCAGAGTGCCAATATAAGCCTGAACAGCACGCTGTCCTTTACTGTTATCAGCGATATAAACATGAACAGGTTTATCGTCTTTTTCAGATATACTGACGTATACAACATCAGAAATATCTTTTCCGGGATAGTCTGCGTCAGAAAAAAACATTATGTGTCGTCTCTTTCAATAGCATTAAAGAATAAGCTGGCGTTTCTCTGTACCATTCTGAATCCAGACGCCATTACCGGTGATTGATGCCACCTTATAAGAAGTACCAGGAATACTTTGCCCGACCTTGACATCAGTTACTGACCCGTCCAGGAGAGAAATTCGTGCTGACATTTGTTGTGGTGTACCAAATATTTCAATAACACGAAGTGATTTAGCGGCTACACGTTGCGCTGGTATATCGGTATCTGCAAGAGAAGAATACCCGGAAGAGGGCGTTATTGCTGGCGTTGCGGACGCTACACCATTCAAATCAAGATTACGCAATTCACTGACAGCCTTCTGACGCGCCAGCTTTGCTTCGTAAATTACCGTTTCAGCCTGAATCTGCTCTACCTGTAAAATGGTCGGAGATGAATATTTTATTTCGTCTTTTGGCGTGCTTTCAGATTCTGTTGCGTAAGCAAGTGAACTGAAAAAACAGACAGAAATAATCGTTGGTTTAATTATTTTTAACATAGAATGCACCTTTAATCTCATAATTCAGTCTTCCTTCTTCCAGTTTTATTCCTGCATACTGGAAGCGAACGGCAGGTTCATGAAAGTTAGCGAAAAGTATTGAAGGCGGGGTGCTGGTCTGAATCATTAATTCATACTCGTTCCACGGGAGAATTATTGGACGGCCTTCTTCATCTGTTTTTGTGTTTTCAATTTCCTGCCAGGTGAGTTTTAACCTCATTTTTTGTGCAAATGTTGTCAGGCGTTCCTGAATATCTGTTGCCTGTGGCAATGTGTCCGGGGTAATTGGATCAGGTGAAACGTCAAAACTGACAGGAAGAGAAAATCCCCCGGCGCTGCCTTCTGGCAATACAAAAGTTGCTGTGGTTGTCCCCTGGAATATTTCTCGTATCCGTGTGCTGAAGTCCTCGACCGTAACGCCGCTCAGTTCCTTATATGATGTGCGTAACAGCCCGTCATTACCGGATGTGCTGCATTCAGCAAGGTCAAAACGCCATCCGGCGATCGATAAGGGCAGTGCATCCCATTTATCAATGCATTTATCAATAAATGGTTTTATTTCAGGTAAATGTTGCCACGGTGGGGCAATCTGTATTGCCTGTTTGTCTGCCATTTCTTTTGCCAGACGAAGACGTGCAGCTTCTGCCGCAGCTTCTTTTTCCCGGTATTCCTGGTACATGGTGATCCCGTTCCAGAGCAGTATGGCCAGAATGGCACTTCCGCCATAAATCATGAACTGACGTTTTCGGGATACGCGAGTAAACGCCGCCTCTTTCGGCGGTTTTTTTACGTCAATCAACGCAGACAGAGTGAGTGAGGGGAGTGCCTGGCTGATATCCCATGATTCAGGCTGGTATAGTTTCCAGCCGGGTTCTGGTGTTTCATTAAACTCAAGAAATGTTGTCAGAGCACTCATGATCTGACTTTTGTTACCTACAACATCGTTGACGAGAACGTTATTGACGCAGGAAACAAAGCCATAAAGATCACCGAGTTCGCAGATAGCGTATCCGTCAGGTTCGATGAGGGGTTTTATCATCAGTGCCAGTGACCAGAATGAGGACGCCCCTGTGGGAAGATCCTCTTTTCTGATAAATGCCACCATCGTTTTGGTTTCATTTTCTGAATCCTGATACTGACAGGAGAGAAAATATTCAGCGTCGTTTTTTCTTGCGAATGTACGCGCATTCTTTTCTTCTGAAGGAAGCGTTTTCCAGTCCAGACCGGAGAAAAAAGCACGCTTGTTTTTAATAACCGAAGTGAAATTCCGGTTATTCTGATTATTCTCAGACAAAGTGACCTCCAAAAAAACTCCCTGTGTTTTTTCAGGGAGTTTTCAGTTAATTAGTTCACATTTTTTATTACTGCACTGGAGCCGGAATTAAGCACATTTGGTGTAATCAAAACGACCAGAATCGTGTCGCCGTCTTCGCTGTTAATGCCACCCCCAAGTCCAAAGAAGGAGGGGGTTATTACACCTTGCTTACTGGTTTTCCGGTTTGATTGCTGGAATCCACTCAGAATAACTGTCTGGCCTGATTTCAGGTCAATAGTCTGATTAATTGTTTTCAAATCCACATTCGGTGTTTGTGCTTTACTGCCGCCACTTTCGAACACCTCAAAAGTAGGTTTGTCAGACAGACTCATTGAGAACAGCAATTGCAGGTTTTGTGAATCCGGCTGAATGTATGGAAGCAGAGTCATATTGAATCCGGTCGTTATTGTTGCCGCATTCAGTGACGTGGATGATCCCACATTGGCGGTGGAATCGGTTGTGACACTTTCAATGAAGCCCTGCTGATTGGCAATTTGCATAGGGACGGGCGTCAGGTTTTTAGTCACAGCGGAGTTTTGCGTTACGACACTGACGCTGCCTTGTGACGACAGGGCTTTCAGAAATGCTTTTGAGCCAGCCAGCTTACCGTCCACGATGGATACACCACTGGTAATAACATTCTCGGCGGCATTACCAAATGAGCCACCAAGACTCAGACCAAGATGACCATCATTAAATACTGCGTTCCAGTCGATTCCCGCCTGTTCCTGGCTTGATTTCTTGATACTCAGGATTTCAACGTTCAGCACAACCTGACGACTCATTTCATTATTGCGCTTGTTCACAATCTCCTGAACGCTGTCCAGTACATCCGGGGTATCTGTTACTGTCAGTGAACCAGTGGACAGATACATGCGCCCTGTGCCTGGTGTCAGCATTGAGGAAACTTCGCTTTTCAGGTCGTTGTACAGCGATGATTTCATTTCCACGGTTGTAGTTTGCGTATTTGATGCGTCGCCAGTCATACCGCCAGAGCTGGTAGAGCCGGTACTGCCGCTGGAAGACATTGTGCCGCTCACAACTTTTGAGTTGTATGCCACATTGCTGTCCATATATGTGATCGGGAATGAGCGCGTTTCCAGATAGAAGAATGTGATACGACCTTTATCGTATTTCCAGGATATTCCCAGCCGTGAGGATACGGTTTGCAGTAGGGCAGGAAGCCCGGAGATATTCAGTTCCGGGAGTGATGTACCGCCAGAAGAGACTTTTACTGGCTGACTGCCTACTGACGCCAGAGGAACCATTCCATTTGCATCCGGTGCTGGAATTGATCCTGTCATTTGTTGTGTTGAGCCAGTGCCTGTACCGTTCAGATAGGTCCAGACATCGGCGCTGATTCGTACCGGGATATGACAGGTTTGTGTGATGCGTTGTGCGATCTGGCGTAGCTCAATATCTGAACGAGTATTAATCACAATCGGGCAGGGCGGCGCTGATTGCTTTACCTGCTTTTCAATAATTGGCGTTGTATTAATCCATTGTTTTGTTGAGTCCTGGATAACCGGGTTATTACGAATTTTTTTCGCGTATTCAGTCGCTTTTTGTGCGTCTTTTTCGGCTGCAAGTTCATTAGTATTGATTTTTTTGAAGTTTGCACAGCCTGATAATAAAAAAGAGGATATTAATAAACAAAGCAATTTTTTATTCATATTTAATTAACTCCGGTACTGGAATGATAACTGTGCATCCTATTTCATCGTGAGAGCGGGTTAACGATATAACTGTGTCGAAAAGGTTTTGTATCTGCACGCCGCCATCTGAAGGAGTCGTGACAACAATAAGACGTTTGCCACGTTTCCTGGCTGTTTTAATAAATCGAACAATAAACTGAACAACTTTTGATTCCCGTAAGTTGTTACCGATGGCTACACTATTAAGAACCAACGTTTTTTCAGGTGCAGATAAAAAGTCAAAATAATCAAAATTTTCATCGCAAAGATAATGGTGTTTTAGAAGGGGAAGGTGTTCATGGCGTTTATAAATATCACAGAAATCAAAATATTTAACGTCAGTGTCATTGATTAGCAATTCTGCTAAAAGTGTTGATTTTCCAGTTCCAGTTTCGCCTATAATCAGAATATGTTTTTCAGAGTTTATCAAAACATCAATGGCTTCTTTCATCATATTCATATTCATATTCATATTCATATTTATCATATTCGAGTGTTTTATTCTCATAATAGAATCCTGTCTTTGCTAATCAATAGTATCTTTATTGCGAGGATTATTCAGATACGTCTGTTTTATCGTGATTATCAAGGTCTGGATTAGTAATCACATTGCCTCCGGCAACCATGACTTTTTTTCCTCCAGCTTTAGGGAGATAAATTTCTGCAATTTTTGCTATTTCATCCATGACTTCTTGTGTAAACAGGTTTGGAATGTCAACATGATTGTTACGGGCGATATCCACGCGGCGTTTAAACTCTGGTTCTTCAAACCAGATTATTTTGTTGGCAATAAATGGTCGGCTAAATTCACTGGTTATAATTTCACGGATTGCAATATTTCTGGCTTTATTCTTCTTATCCCGGAGAGACACAATTTCTTCAGGAAGAAGAACCGGGCGCTCTATTACATCATCATTCCGTGTACGTGAACTTCCTCCCTTACCACCACCACTGGATATTGAGCGTTTGGATATTTTCATATCACGCACCCCAATCTCTTCTGAAATCTTTTTAGCCAGAGCATTTTTTGATTTCGGCGGATAGTAAAGCACAACAGCACTGTTTTCAGTGAATGTGGTCCATCCTTCCTGACCGTACATATCGCTTTTTTGCCCTTGTCCTTCATTCTGAAGAATGAACATAAAGCGAAGGTTATAACCTGCTGTAAAACCAACAGCGCGTTCAATGACCTCCGATTTACCCATTGATGTAAATTCATCCAGGAGAATAAGGCACTGGTATTTCAGGTCCGGGTTATGTTCCGGCAGTTCACGACAGTTTTCATTAACCAGTAATGAGAAGAAAAGGTTTACAATTTTCTCATGAGTGATTAAGGCATCCGGTGTCAGACCAAGATAAATTGACATGGGTTTTTTACGAATATCCCGGATATCAAAATCACTGAAATTCGTCGCTTCAGCAGTAACAGGGTTGCTGAATATATTCAGTGGTGATGAAAAGTTCGTTTTTATACTTCCCCTTGTGCGATCTGGTGCTGACATGAACTCAAAGAAGAATGATTTTGTTTTATCGCTAATCCAGCTACGATTTTCAACTTCCTGTCCCATCCATGCGGCGAGATCTTTTCCATTATCGGGAATCGACGTTTTCAGTATGGCGCTGATGGAAACAAGGACGTCCGGTGCATTATGACCTTTGGCTTTCTGGTCCAGGTGAAAACGCTCCTTGTCCAGCAGGTAAAGCCCTAACCCGACAAAAAGGTTGCGTGCAGAATCAGACCATATAGGATCATCACTTGCCGGGATAAGGATTGCGGCAATTTTTGCCAGGTCCGTTTCACGAAGTAAATCACTGCGACTGACGCAATCCAGAGGATTCCAGCGGTGAGAGCGTATCTGTCCTTTAATTGCCTGGTCGATAGTTTCCGCGTATCCGGCGGGCGCAAAGAGAAAGCATTTTTGCCCTAATTCTTTTTGCCTGAATCCGGCTGATAAAAACCAGTTTTCAAGTTTGATATCCAGTATGACCATACTTCCTGGATAGTTTACGCAGTTGGGGATAACAATCCCCACCCCTTTACCAGAACGTGTCGGGGCATAAAGAATTAAAAATTGTTGTCCGGCGAAATAAATAAATTGTTTTTTGTATCTCCCCTTGAACATTTTACCGATGAGTATTGGTGGATGTTTGTAAGGTGATTTTTTATCCGGGAAAAAACCTGATTTACTCAGGTCCATATCAGTTGCGAGCCGGGCGTCACCGTAGATGACTTTTTTAGGCATAATCCCAATCACGACAATTAACATGAATATGGCAAAGAATATTAATGGAACGCTGAATCCAAACCATGCCATAAAAACGAGTTTTTTATATTGTGGGTTGCCAGAAAATGTTGAGATTACAGAATGGAATGTATCCCACTTGAGCAAAGACATATCAACGGATGAATATCTTAATGCTGTGTATCCACCGAGATAGTTTGAGGCTATAAGCACACCCACAAGAAGCAGTAACAGGAAGAGAATTAACCCCCCCATCTTTTTTGCATTCATAGTTATCTCCACAATTTACATTGTGCTCAAAAAACGATTTTTTTCTTCGGGTTCGTAATACAGTTCAGTCATTCTGGTATGGTTGAAAAAAAGAATGACATCAATTGATGTTTTTATCGTGCGCATGATGAGGTCCATATCAACAGTCATACCAACATCAGATTGTTTTACAATACTTGCCAGTCGTGAAAATGAGGCATAAGTATTATTAGCATGAATGGTACTGATTGAGCCTTCATGCCCTGTATTAAGCGCCTCCAGATAACTCCATGCTTCGTTACCCCTAAGTTCAGCAAGAAAGATGTGATCTGGCTTTAATCGCATACACGCTTCAATGATTTCCTTCGCTTCAACGGTATTTTTTTTGTAGAAAAGCCGGATGTGGTTTGGATGCAGCGGCAGAGACATTTCCGGTACATCTTCAACGGTTATATAACGCCGTTCAGGTGGAAAGATATCTGCAATTGCCTTTGCTATAGTTGTCTTACCGGAGCCAGTCCCACCTACGATTAAAAAATTAAGACGGTCTTTAACTGCCTCTCTTAAAAATTGAGCTTTAGATTGACCATCCGGACGCCGGGATAATTCATACAGGTAGCGTTGCCGTTCAGTCAGTATTGCTTCGTGTTTTGTTGCAATCCTGACATTATCAAAACGCCCTGTTCGGACATAATCATCTATAGTGAAGCGTGTTAATGATGGCTTGCGGATAGATATCACAACGCTGTTATTTTCCGTTGCAGGTGGAATGATGATTTGTCCACGTTCCCCACCGGGAAGAACTACCGATGCAATGGGATTATCATGGCTAAGTGGTATTTTTATCTTTGATAAGTTCGTCAGAGTTTTGGCAAGTGTCATTAAATTATCAAAAGTCGCGTCTGGCGCATCTTTAGATTCCCAGCCATTTTTCCCTTCAAACCATATTGTTCCTGGTTTATTCACAGATATTTCTGTGACATGTTCAATATTAAGAACAGACTGGATACCCGTCATATCCAGATATCGTCTTGCGGTTTTACTGTTATCTCGTGGTTGTGATGTATTACTGGTATCTCTGATGTTATTCATATCAATTGCTTTACTGAATTACTGAAGTTCATATACGGATGAAAAGTCGATATTTCGCGGAACAATAACAGACAATACAGTCCCCTGATTAATATATGCCGTGGGGGGAATATTTATCGTATTGTCCAGCGTTGTTTTTGCCATTTCTTTTGTTGCGTCAGATGTGTTTTCGTAGGTGATATTGCTGTTTGAGCCTGTTTGTTGCGTGCTGTTTTTCAGAATATCTAAACCATCACCAAGTAATGACAGCAGCAATGCACCACCAAAACGCTGACCAAAATGATTATCGACCCATGCAGGAAGGCCGGAAGCGCCCAGACTGTCAGTGCCAAGTGCGCCAATGCGCACATTGACATTTTCATCTTTGAGCGTTGTCCAGTTAACGAACACACGGGCTACTCCCTGTGTCATGACTTTATTCTGCTCCCCGATCAGTAATGCTCCTTTACGGGCAAGAAGCACTTCGCCGTTATCCGACCAGACGTCTCGTGTTAACTGACACATTGTGATACCGGGATAAGAGGTTACGATTTTCGTTTTGAGCACACAGGATAATGCTGTTCCCGCAGATAACAGATATCGGCGGTTCAGCACAGGGGATACCTTTCCGTCTGCGTACTGACTGCCCTGTAAATCATCTCTTTCCTGGTTTTTTTCGCCTGGTTCCTGATTGTCGATCTTCACCATTGTGTCGCCCATCAGTTGTCGGACGGATTTTGGCAATGGTTGTGGTTGGTTTGGATCTCCACTGGCGGTTTGGGGACTGCCGTTGGCTATCACATTGACGGCTGTTTGATCGGCCTTTTGTGATGCTGGCGCTGACGGGGCATTGTCTGCCTGTTTCTGTTCTTGCTCCTCCTGTGCTTTCCTGCGGTTAGCGGCATCGAGTTTTTCCTTTTCTTCGATGTTTTTCATCATTGTTGTCAGTGAAACGCCATTGTTGGTATTTGTGACCAGTGTCTCGTCAGACTTCTCAAGAGGTGTTTGCTGAACGACCTGTTCTCGTGAAAGAACTTTAAACAAGATAATGATCACAAATAACGCGGCAATCAGTAATATGATGAGGATTAATATTTTTTTTGGTGCTGCTTTACCTTTTACTTCGATAATTCCGCGATTTCCCGCGTCATTATTTTTTTCATCATCTTTATTTTTCATAGCTGAACTTCTCTACTCGTGTTTTTCCGTTTGACGTACCTTTTTTATTCCACGGACGATTAACGATTGATGATGTTCTGATATCTGCAACCTGATCACCCAGGCGCAAACGGAGATTTTCAGCCACGTCATAGTAAACCATTGTGTTTTTATTTCTGTCTCCGTTAACCAGATGTTCATTTCCATCGGCATCAACACGATAAAGGACCGGAATTTCCTGTTTATTAGTCCATCTCATGCATGTAAATTCGCCATTATCCCATACCTGATAAGGGAAAATTGATTTGTCCCCCTTGAGTTGATAATGACCATTAATTATTCCACCATCAGTGCATGGAAATGGCTTTTCAGTCATTACGGTAGCTGACGGTGGTTTCGGATAATTAAAACGCAGAATATACGTGGGTTGTTTCGTGGTTGATATCAGGTAAAGAGGATATATATGCTTATTGGTCTGAACGGTAACGTTTGTATCCGGTTCTTCTGCTTTTGGACGCAGAAACAGAGTGTTGTCCCTGACCGATACTGACCAGGCTTCTGGATCACCAATTCCGACATCTTTTATTGTTTCGTCTTGTCCGAACTTAATTGTTGTTTGTGCGCCTGCTTTTACGCGCACATTAAATACATCCTGCTCGTTATAATCTACGGTCTGAATACGCCCGTCTCGTTCCGATGGTGTGCCGTACATAGCTGCTAAAGCACCAGGTACTTGTAGCATCGACAAAAGAACTATCGCAGTAGTGCGTTTAAGCATCATTTTATAACCTCTGGATCAGGTTGGTAGCTCAGAACTTGCAGTCCCAGCGGATTCACAAGGCGCTCTTTTTCTGTTTTGATATCTTTATCCCAGTCAAAAGAAATCGTCGAAATCCACTCTGTTGCTCTGTATCCGGGGGCGGGTTTACCAGATAAATCCAGTACCATTTTTTTGAAACGCACCTGCGCCATATCCTTCCTTAGAAGGATTACTGAATTAATCTGGACCTTAATTTTGTAGTTGTTTTTCAGAATGTTCAGTGGTGAGGAATCAGCACGAATCATGGTGTCATAAGCTGAAAACACCTTTTGGGAAGACATTGTTTTTACTGTATCGGCCTGTTCCTGGATAGTCTGCCAGTCGTATGCTTCATAGTTAATGAGATATTTTGACAGGAAATATTTAGTCTCAACATCCTGATAACTTTCCTTAGCATCAGATAATTGAGGGGCAATATCAGTATATCCAGTGCTGTTATCCACTCGCACAAGGAACGGTACAGCGGTTTTTAATGGAGCAAGGGAGGCTATTGCGAAACCAAGAATGCAAATTACAACAAATTCACCAATGGCGAGAATGGTAAATGTTTTTGCCTTTTTCTTTATGATTTCAATTTCACTACGCTCAAAGTCACGTATGGCCTTGAAATAATCCTTTTTTTCGTTCTTTTCTTCAAATTTCTTTTTGGAGGCTTCTTTTTTTGAATGTTCATAATAGTTTGTGTTTTCCATTAATAATCACCTGCTTTGATCTGTTCAATTGCTGTGTTTAACTCAAACCATCCCCCTTTTTTCTGCACAGGATTGGGGGTGTGAGAACATGCACTAATTAATAGCGCGGAGGCAATTATGAGGGGGATTTTCATATTGTTTTCCGTTCTGTATAAAACAGAGAAATTAACCGTTCAGGCGTTTCAGGAGTTCTGGTATCCAGATATCAGGAGCTACATCTCCTTTTTCAGCAATAATCTCATCAAAAATAGACAGGCCAGTTTCAGAGCCAGAAAGCACGGGGATAAACTCATCGAATCCAAACAAATCCATTTTGGCGAAAACAGAAGAGCCAGATTGCTTGATGAGCATGGTCCTTGACTCCTTCGTCAGTTCTTTGAGTTTTTCAAACTCTTTTTCAGTAAGCCCAATTTCTTTATATCCTTCCCATTTTGCATCGGGATTGGGCAGGAGAATTTTCGTTGCGGTTTGTTGTACCAGCGCCGCAAAAATTGCGCAGTTTATGGCGTCTTCCGGGGACTGTGATGTTAACCACATCATTTCACCTTTCATGCGCCCCGCTTTCAGTGCGCTTTTAATCATGCTTTGTGTCATGGGAAAGTTTGCTGGCATCCAGAACTCTTCAATAATGGAGAGCATCAGATTGCCGCCGCGTTGCATGATTTCTTTGTAGAAAAACAGGACCGCGAGTAAAGGTTCACAGGCAGGATGAATACCGTTTTTTGTGTCCAGGACAACGGTTGTATCAAATCCCACTTTTTTATGATGCAGTGGATTGAAGGTATTCCGGGGGGAATCCACCGCCCAGGCATATTCGCCGTTGTCGCACCATTTGGCGAGACGCGTCTGAAGTTCAGGACTGACTATATCGAGAAGAAGGGCTGTCCGGCGTTCTTCGACAGGCATTCGCATAATGCTTTCCACGGCAGCGTTTAATTCAATGCCATGTTCATCAGAACAGGGGTTGCCCTGGTTGTCTCGTGCCAGAACCTGTGTCCAGCGTTTCAGAAATGCGAGAAGTCTGTGCCAGACGGGAGATTCCGGGCCTTCTGCCAGTTGCCACGGATTGCAGCCGGTATAGATACCTTCCTGCAATGTGAAATAGCTCCCTCCGTATGCGCGGACAAAAAGTTCGGTAGAACGGTTGTAATCCACAACAAACATTAAAGGGTCAAAGCGTTGAAGAAAGCCGCTGGCTGTTGCTTCAAATGTTGTTTTCCCTGTGCCTGTTGCGCCAAGAAACATTGCGTGTCCGGCAATTTTTTGCCCGGTAACATTTTTTTCCGGTGGGCTGTAATGTGTATTGAACCAGTAAACGCTGCCTGAATCCGTTTTTAAAGGCATTATGGCGGAGCCATCACCAATAGGATTGCCTGATTTTTTCCCGGAGGAATAATTGTGAAAAGACATCAGACAGGCCAGGTTGGTAATTGTTCTTCGGGTATCCAGTGGACGCCTCTTATTCAGCGGCATATGACTGAAAAAAACAAAAGGTGATGCGAGTGAGGCGCGACTGAAGCGGAATCCCTTACCCGAAGTAATAAACTCAGCAGAGAGCTTTATACCATTGCTTCTGGCCTGATCTGGTGTATCACCGAATACCGTTAATGCGCAGTGTAGTGAACCAAACAACGTAATTCCGGCTGCGACCGCTTCTTTACCAGCTTCGAGTTCTTCCTGCTGTATTTTGGCTGCATCATTAGCTGATTGTAGTTTGTTTAACTGTGAATCGATATTCTTCAGGGTTTTTGTTGGTGACTCAAAAATAAAGGATTGAGTAAGAATAAATTCATAGGGTTGTTTGAGAAGGAAATCCCATTGTCCGGGGGTTGTTTCTATCGGGAAATCCTTCAAAATATAATTCGTTGCGAATTTTTTACTATCCGATTCGTTGTTACGCAGTTCAAGAACATCAGCACCAAAATACCATTCAGAATCACAAATACTGGATGATAAGGGGGTGGATGATAAAGGAATCATGTTATGTTCTGCATTTAGCAATAGTGATAGATAGTCTGCCACCTCACTAATATAAGTATTGTATACGGATAATAAAGAAGCGTTAAATGGTAGAAGTGCTTTTTGCGCCTGTTGTGTCATTTCATTCATGCGCTCGATTCCGGTATCAACATCATCGTAGGGAATCCCAAATGTCAGATACCATGTTGATTTATAGAATGCGCTGGATGTGAACAGAGCAAGGTATTTTTTTGAAAACCTGCTTAAAAAATCATTGTCATAATTCCATTCTCCATTGATGGTTGCTCTTTTCTTGATAAGATGAGTCCATAAATACAGGTCGCCTTCCTTTCCTAACCCAATCAGGAAGTTTTTAACGCTGTTAAATAAATTTGTAAGAACATTATCGTTCTCCGTCTCAAATGGAATGCCTGCTACAACAAGTGAAGCCAGCATTTTTCTGTCAGAAGTAAAGATAATACTGTCTGTTACATGGAAGCGATAATCGGGATATTCACGAGCAACATCCATATTGTCACTGTTTTTTAATTTGATTTTGTGCATATATGTTACCTGACAGATGAATAACCAATAATTAAATCTCTGTGGCGGATTTTTAACAAAAGCCCCATAAGGTTTAAACGGATGACTCTTAGTGCGTTTGAGTCATTTTCACAGGCCAGTTTTACTATAAAAAGAAACAGCGCACATATGACAGGAATAATTATCCCTTTTATACCCCAAAGGAAAATAGCGGGGAAGCCTGTCAGAACAGCGAAAAAAGCCGTGAACAATAACAACATTAAGGGTATACCAGCAATTAACGCAGGACGGTTAAACCCGTTATAAGTCATGAATGACTCACTGACATGTTCATTTACTTTGTCCATAACTCACCATGAAAAAAGCCGCAATTCACACATAAGGTAAATATGCGGCACAGTTGTATATATGGATTACTTATTTGAAGAGTTCCAGCGCCCAGTTCCCGGCGTAAAGTGCACCGCCAGCGAGGGAACAATAGACAAGCGCCATTCCTACATCAGACCAGCTTTTCTTCTCCATGAATGCCATGATGACGTTATAGATCAGGTAACACAAAGCACAAACACCACCAAAGCTAAATGCCCATGTCTTAAGTTCTGTCATGGTATTTGTTGCGGTGTCGAGTCCACCTGCATAGGCAACGGAGGCGGTTAGTGACAGGAAAAACGCCAGGATAAAATACTGCAAAGTTGATTTGACTTTCATTAATTAAAATCTCCAAAAACATCCCAGTCCGGTGATGTTTTCTCTTCATCATTCGGGGGTTTTATTTCTGATTCATCAGTTTTTTCACGGATTGATGGCACGATATAGTTATTCGCGTTACGAATAACCCGTTCCAGATAAGAAGTGTTGTTGAATTTCTTTTCTTTAATAAATCCTGTTGATTCGTTACCCGAATAATACAAAGATAAAGCGCGGAGAATTTTTTGATTACTACTACCCTCCTTTTGTCTCAGATAATTGTCCGTCAGGACTGCCGCTCCTGCTTTTATATTTGTACACGGGTCAAAAACTGTTTTATTAGTCAGATTGAGTGATGGGAAATTTTTGCTGTATATCTGTGTCAGACCTGCACTAAATCTTTTATTTTCTGCTGTTAATTTTTCTGCATATTCAATAGCTCCTTTTTCATCTTTAAAATACTTACTTGTTCCGTCAGAAACATTCGCTATTACATATGGATTAGCCCCTGATTCAGTCATAATCAGGGCATTCATCGTATCGGGTGATACATCAGGGGCACACTGACTTATCAGTGCTGCCAGAACAGCCGCTGATAACTGCATAAGGGATTTATCTCCTGATATTAAATTCGTGAAGAACGTTTTTTCCTGCGGGGGCGACGAACGATACTGTCCAGCTCATTAGCGCCGGAAAGAAGTTCTTTCAGCGAGACAATTTTTTCCCTGACAGGTATAAGGGATTTTGCCCCCAGACATTGTTCAAAGTCTGATTTGTACAGGTCGTATAAAACAAGCGTATGTTTTACATCCTGTGACAGATAATTATGTTTATTCTGGATGTTACCTTGTTCGATATTTTTGATAACATCTGCTGAACGATGAATTAAATCTGTATAGATTCGGGTGTTTTTAAGGGGATTTATTTTTATATATTCAGAAGCAAGCTGAATAAGATTGCATCGTAAGATAAGGACTTCACGAGTGGCAATATCAAGCTCGCTGAGATCATCATTATCCTGCATGGGTATCTCCGGTAAAGATTTTCTGGACTGAAGTCCGCGCCGTAGCGATATATCCGGCCCACATAATGATGTGTCGGATATGCAGACTCCAGTTCAGAATCCCCTTGTCAGGGGATATTTAGTTAATGAGTACAATAGCCTCTTCTGGAATGGAGGAAGGAACGGGAACGCCCATATCATTCCCCTGATTATCGACAATCTTCTTATTTTTTACTCTTCCTAACATTGCTGAATGTTTTGTTTGCTGCTGCAAAGCAGACATCAGACCTTCTGTATCGGCAGACCAGATATAAACTCGCTTACCGTAAATAATATGATGAATATCATAAACGGGTGTTATACCGATTTGCTCAGATGTTAACGTTCCTCCTGAATTACGTTTGTCTGGATATTTATAAAGATAGTCATTGATAACATTTGCGAAATGGACAAATTCAATTGCTTCCTGTTTTGCAGGAAGCAACTTCATTCGCTGTATGGTTTGGTTATTGTTATCTGTCAGATAGTAACTGCCTATTGACAGGAATATCAGGAGCAAGCTGAAGAACCAACCCATAGCGATTACTCCTGTTGTGTGTCTTTAATTGTGATTACGCATTGTGAAATATTCCTGAATGCGTATAACGGTTTATCTGCTGAAAGATAAAGCATCAGCGTATCTTTTAATGCAGATTCAAAGTTTCCCCGGAAAATAAGAGGAGCATCTATTCTGTAGTTCACAGGAGTATCCCATACAAGATTCCAGGTTGAATTACCACACGGTGAACTTGACGCCCATTCGGTGAGGCCATCCCGCAGCGTCGAACCTTTTTTAAGATTCCAGGTGAGTTTTTCTGTCTGTTTTTGCGGAACGGTGACAGGCGTTAGCGGTTTTTGAGGTAACTTGTCAGAGGAAACTGGTGTCCTGACAGGAGATGCAGGGGTTTTGTCAGGAGATACAGGTGCTTTTTGTGATGTTTTCCCTTTCTGTTGTTGAAAATCAATCAGAAGATGTCTTTTTTCGGTGTTCTTAATTACCGTCAGACCGTAGTTGCGAAGCATTTTATCCAGGACATAAGGCCATTGATCGTTGCCCTGCCAGGATATCGTGCGACGGAAAGTTTTCGTTACCTCCGGGGATATTTCAGCCGTCCAGTCCGATGGGACAATTTCTTTTAATGCTTTTTGCAGCGAGCCTTTACCATCATGGCGAACAAAGTAAATTTGCTTTTCAGGTGTTGATGGGCCTGTAAGCGTGGTGGCCGGAACAGCGTTGTTAGCGTTTTTTTTCAGAAAACCGCTTTTTGACATTAGCGGTGGTTGTTCAGGACGCGGTTTTGTCTGCTGATCAGTCTTTTGTGCCGTTTGTGCCTGATGGTTGTTGCTACTGATTGGCTTTGCGGGAGAGAGTGCTGGTTGTTCAGAATGCTGTTTAGTTGCTCCTTTGGCTGCTGTTTTTATCTCACCAGATGAAGTTATGCTGTTTGTTGTCGGCTTCAGAAAACTATTATTGCTGCCCTGGTTTTTTGGCGCTGTCGCTGATTGCTCAGAACGTGGTTTTACGGTTTCTGCAATACGAGGCGCGGGAGTTACGTTTATGGCTTCCTTTTTGAGATAATTATCATTGCTGTATTGAACAGTGCTGTTTGGCTGTGACACGGGCTGCTGAAGGGCCACCCCGTTATAGTATGTGTCTGCTCTTACCTGCTCAATTGTGGTATTCAGTTCATACCAACCTCCTTTTGCCTGTGCGGGTTCAGGGGCATGGGAACACGCATTCAGCAATAATGTGGTGGGAATAATAATGAGCTTTTTCATGGTTAACCCCAGGAACGTCCGAACTTACTCATGATTTTTGAGATCGCCGCAGGGTCTGCGGTCGTGCATTGATTAAGAAACGCTTTTCGGGCATCAAAGGTTTTGCTCCAGCGAATAGATCCCTTTTTCTTTTTGATGATATTAAAGAAGTCCTTTTCGGCGCTTTTGCATTCGCTCCCGCCGCCGCTACCGATAGCTTTACCGTACAGACAAAGAACGGAAGCACACGGATCAGACGCATATACGGGGGCAGTGAGTGCGATAGCACTTGCGGCAACCATCGATAAAATGATGGATTTCATGGTGTTCCTCCATATTTCAGGCAAAAGAAAACCCGCACGAGGCGGGTTACTTGACAGGTTTAAAATATCAGCGGGTATCTGCCATGTGATAGACGTCGCTGCCTTCTCTTTTTCCCACAGAAATCACAAGCAAAACGACCTTCTCATCAATTACCTGATAAACGAGCCTGTAACCGGATGCGCGAAGCTTAATTTTGTAACGGTCAGTATGACCATGTAGCTTGGCTGCGGGGACGCGTGGATTTATCAGGCGTTCTGACAGTTTCTTTTTGAATTGCTCACGAACAGTGTGACCGAGTTTTTGCCATTCCTTCAGTGCTCTGTGGTCAAATGCCAGTTCATAAATCATCCAGGTTAACCCTTACAATGTCTGGATTTTTCATTCTTTCATCAGCAATTGCGTTGAGTTCAGCGTCTTCAGCGAGTTCCCGGTAGTAGGCATAAAGTTCTGGTGGGACACAGTAGAACGCTGGTTCATTTCGGTTCAGGATTGCGACGGCATCCCCTTCTCCTTCGGCTACAGTTCCCATAGGATTTTTTTTCAGGTCAGTAATACTGGCTGCTGTCGTAGTCAGAATTTGGTAGCTCATAAGTCCTCCATTTGTATCATGGAGATTGTAGCACCTTAAAAAGACTTTTAAAGGTGCTTTTGATTGTGGGGAACGACAAAAATGATCCGGGGTTGGGAATCAACGCACGTGTGGCGTAATGATGACAATCCGGTTCTCACTTTGGCTATCAGCATTAACTCCAGTAAAACCACTCACGATAAGGGGCTGGTCCGGTTTGAGTAAAATATTCTGCTTGACAGTCAGTTTCCGTATCGGGTCAGTGGTTGTGAGTAGCAGGTGGAAATTTCCACGCTCAATCTCCGGTGCTATCCGCATTGTGAAGCCAGCAGTCCGATCTTTTTCAGCCAGTGTAGTGTCACCGGATATTGCCTGACGGGGGATCAGTTCCGTGGCAGGTTCCCAGCTTCCGGCAGATGTTGTTTGTGATTCAACGCACGGGGTACTCTGCATTATTTTGCGGATTGTTTCGGAGGATATCGCCTGACCGTCATCAAAGAATGCACTACGGAAAATCACGTTCTGATTTTTGGCAAACAAGCCTGGATGCCGCTCGCTCAGACTTTCGTATATATTCATACCATTCAAACAAACAGGAGAACTGTCCGGCAGGTCTGTTCGCACGGTATCAATTTTCAACGTTATCTGGTGGTCATTTGAGGTTAACGGTGACTCAGGTACTGGTTGTGTTTTGCTATCCGCGCAGCCTGACAGTAATGCGGTGGCGGCAACCATCGATAAAATGATGGATTTCATGGTGTTCCTCCATATTTCAGACAAAAGAAAACCCGCACGAGGCGGGCTTTGTGACGGGGGGAGAGGAGAACGTTATTGTAGACGTTCCATTAATTCATTTACTTTCTTCTTGTGCTCTTCTTTTTCCTTGTAGGATTGTCCAAGCATCTGATTTATAATTTCTTTTCTCTTGATATCATACAGTGCTTCGTTGGCATTCTTACAGTTATTATTTGTAATTTCACCAGCTTTGCATTTATCAGATATCTTTTGGGCTTCATCCTGGTGTTCTTTATAGTAAGAGACATCATAGATTTTGTCCTCACATCCAGTTAGCAAGCCACAGAATAAAGCTAGAGAAATGATAGTACCTTTATTCATGGTATGTATTCCTTAATCAAGGCGTCTTCTCATTTCACTAACTTTCTTTTTATATTCACTATCTTGTTTTTGCTGAAGTGCAGCACGAGCATTATCGCAGTTTTGATCGGTTATTGTACCTTTCTTGCAATCTTCTAAAGTTTTAGTGGCTTCACTGATGTTGTTTGAGTAATATTCTACAGAATATATTTTTTCTTTACATCCCACTAACATTAAACTGGCGAAGATAATTGATGAGGCGATAATTGCATTTCGTGACTTATTCATATCAGCTTAAATCTCTTCTCTTTTGGCGAACATATGTTTCATATTGAGCTTTCCGCTCATTCTCTAGTTGCCGTTCAGCAATATCATATTGCTTCAATTGTAGCTCCATTGTCAATTTTTCTGTATTCAATTGGTTAGATAAGTCAGCTTTTTTTTGCGGAGTATCTGCTGACGCGAACTCCTTCTGTAACGTGGTTAATCTCTCAGCCCTTTCATGCATTTCATTGTTGAAATTTTCATAGAATTTGATCTTAGCCAGGATTGCATCATATTTTTGTTGCGTCTCTGCAACGTTGCTTGATAAGTCATATTGCGATCGCAAAGAGCTAAGATCTGATTTTGCACTATTTGTTATTGTTGATAGAGAATCCTTCATATAGCTGGATGCGCTATCAAAGCCATTGCTGAAATCAGTGAAGCCTTCGAGTCTTTTCTTCTGTTCTTCAGCGTATCTTATGGTCTGTTCATATTGTTGCTTGAGTTGCTCAAGCTGTCTTGCCGCTTCAGCGGCACGATTTAGCCCTTCTTCAACTGTTTTAGCTATCGCCGTAACGTCTACTACTGGAATACCAGACGCAAAGATTGATGGTGATGTTATGGTAATTATTCCAGCAAAGAGAAATGATAATACTTTATCTTTCATGTTAATCACCCCGCTTTAAGTTTACTTCCACGGTTTAATCCCTGAACTAATGCTGATGCAGAACGACTTCCTGCATTAAAGAATGGATTTGGCATTTTGCTGGCAAAACCATTGGTGAAAGATCGGAGTCCTGATGCTCGCATAAAACCGGATGCAACCTTACCTCCAAAGCCATTAGCGGCTGATGTAAGGCCATTTATACCTACCCCGCCAGTAAGAGTTGAGCAGAGAGTACCTACTTGCTCAACAAGGAATATAGATATAACAGTCACCATAAAGAAATAAACTACTGTTGTGAGAGTTATATTTCCGCTTGATGGGACTGTTTGCTCTACAAAGCTGATGACGAAGCTGAACGATATACTATAAAAAAGATTTAGCAATATATAATTTAAACAGCTACCTACCCATGCGCTAAACATATTTCTTGTCGAAGCAAATAAAGAAAAACAGATAAATAATATTCCGGCGGATAAGAGAATGCCAACCATGAATGTTGATAGGGTTAGAAATACAGTGGTGTAATAAATAAGCAGCAGCCCGCCAACATATCCAACCCCCCAAATTAGATATATGTATAGTTGATCTGTGAAGCTATACCACTCCAGAGTATCTAACTCATTATTCTTAAAATCATTTAACGTTACTGAAAGCATATTCCACAAATTATCAACACTGGTTGCAGTTCCTGAACCTCCAGTAACAGCAGCAGAAAGGTCGGAGCCAGCGTGCATTACAAAAGGAATAACAAACTGGCTGTAATAAGGCGCTGAATAAGTGAATGCACCAACCAGGGCGAATGCACCGATATTCTTAGTCACCTCGCTCATCAGGACGTCACGTTGGGCATACGTGATTTCATATATTAGATAAATAACGTATAAGGCTATAGCCGCAAAAAATGTAGGAGATATTGCCTGTGCGACCTTAGCGGCGTTCGTCGCCGCTGTGGATTCAACAATGGTTGTTACTTTCTCAAGAAATTCTGAAACAATGTTCATAGCTTAGTCCTTCTGGCAGATGAAGAGTTCACCAGAGGCACTAATATTTGGCTTCAGCTTAATTACCAGGTCTGTGCCGAAAAAAATTGCTTCTCCAATTTTTTGGAAATTTAATGTGGATTCTTCTTTATTGTAACTATAAACGTTGTGGGTCGCTGTCGATGTTTTGGTTTTACGTGGTGTAAACGCTTTTTTAATGATGTAATCGAGAGCGTGGCTGTTATGAGATGGTAAAGTGATTAAATCGTCGTTTTTTTTCATAATGTAGTTCTGATTTTTGAGGTTGAAGATAATTGTCGGTCAATGCTTTTGATTTTTTTTCGTATCTCAATTGGATTCGCCTCAGATAAGTTAGCAATGGATAAAGCCAGTTCGATTTGTAAAATTTTCCTTGTTTCGATGAGATTGATTTTTACTTGTGGTATATCACGGAAGACACCACAGCCTGTAATTGCTTTTGAAACGACACGTTCTGTATTCGCTATGCTTTTCTTTACTGTTCGAGGGGGGACATAACGTGCACGGCGAAGTTTTCTCAGATTAGCTTTTGCTTGAGTCATATTTGTATGCCATGAATAAATAAATGCTTTCTTCAAAGCCTGAATAAGCATAAACCAGTAATATGGTGGCTAAAGGCAAAGAAAAGGTGAAATGAATAACGGCATTCAGAACTGAACCGGTTAATGTATATAAGACAAACGGTGCTGAAAGTATGAAAGCACCCAGCAGGAAAGAAAAAACATTGCACGCCATTTTTAATAAAAATATTTTTATTAGGTGATGTTTTTTTCTGTACATTTCATCGTATCCTTCAGGGGGCTGTCACCTTCTCACGTTGTTGCTTCCATCGCGAGAGCTTCAACAAGTTCATTGATACAAGTCCGTTCTGCCGGAACTTCCCGACCTGTAACCGGGTCGAAAACGATATGCTCATTTTCAACGTAACGCAGGCGATCGTAGATTTTTTGACGAGCTTTTATCTTAATCACGTCCAGCTCTGCATGTTTTAACTTTTCTCGTCTATCGCCGATGAAAAATATTATCCATATGACAGCCATGATAATTGCCGTACAGGGGAGCATGTAGCTTAATACTTTTACAGCCACAGCAAATGTATCTTTGGCCCTTCCTGCTGCTGATTCAAGGATAAAAAAAGCTACAGTTATTATTATGACTGTGTATGTCTCGTATTTATTTACACCTCTAATACCAAATTTGCGTAATGTGGCTTTAGTTACAATCTCTGAAAGAGAAACACCTTCATTTGCGTTTCGCATTGAATTTTCCTCATACTATTCCTGTGACACTTAACACCATCAATAAAATATGTAATGCAGTAATGTTTTTTAGCGTTTTTTTGTAGGCTTCAATAAAATCCTTATCCAGAAGTCTGATAATAAAAATACATAGTGCATTCATCGCTGCTCCTGTAAGCGGAATTAACAACGAAAGATGCATGATTTATTCCTTGTTTTCACTCTTGTCTTCGATGGTCATTACTTTAAGAGTTATTCCTTGCATGTCTGTAACAATAACTCTTGTTCCCGCAGGGTAGTTTTCATTGTGTAACGTCTCAACAGGCCAGAATGTATCACCAATGAGTAATTGCCCCTTGCCGTTAACAATATCGTCACTGAGCGTAAATTCTTTTCCTTTAAAACGTGAATGCCCTGTGTTCACCAGGAGAGTGTCTAATTTCTTATGTTTTTTGTCATAGAAGAACTTAATTATCGACGCCAGTATGCTGATTGACGCAAAGAAACATATATTGGCTTCTTGTGATATCGGAAGAAAAAAGCCCATAATGGCGCTTGATAATGCACCAAGACTGATTAAGAGTAACCATCCCGTTCCGGTTATAATTTCAAGTGCAATACACAGAAGAAACAGTGTAAACCATAACATGGCTAATCTCCATGATAAAAGGCGGAGAGTGAATAAAGATAATAAAGGTGGTAACAAACGGCTAATACATTAAACAACAGACATAAACCGAAGAATATGCCATTCAAACTCCATATAAGCAGAGGCATCGTTGCTATCATGAGCGTCGAAGATCCCCAGTCTAGCCATCTTGGAAATATGGAGTCGATAATATGCTGTAGTCTTTCTCTTTCATTATTATCTCTTGTAATAGAAAGGAGAAAAAACCATGTTGACATGGGAATTATATACAAAATGATAATGATGCATGTATAAGTTTTCATTTTAATGGCTCACTCAGGAGTTAAGATAAGGACGGTGATTAAAATAATATTGTCACCGTCCGCTATCTTTTTTAAGACAAATATTGTTGAGCCTGTTTCATATCAACTTTGGGAGTATCCTGTGATACGTTTTTTAGCAGTTCACTGATACCTGCAACCGAACTCACCAGGCTACTTGAATCAAGAGGCATCATAACCAGTTTACTGTTTGATGCAGTGCCAATTGCCTGGAGTGCTTCAGTGTATTTCTGCGCAATAAAGTAATTAACAGACTGAACATCACCTTCAGCGATTGCATCCGACACCAGTTTTGTTGCGCGAGCTTCCGCTTCTGCCTGACGTTCGCGTGCTTCAGACTGAAGAAATGCTGATTGCCGTTCCCCTTCCGCTTTCAGTATTTGAGACTGTTTTTCCCCTTCTGCCTTCAGAATTTCTGACTGGCGGATACCTTCCGCTTCAAGAATCCGGGCACGCTTAGTTCGTTCCGCTTTCATTTGCGCGTTCATCGCTTCAGTCAGTTCTTTTGGTGGTTTAACATCCCGAATTTCAATACGTGTGACCTTAATTCCCCACGGGTCAGTCGCATAATCGACAACCGTTAACAGTTTAGAATTGATGCTGTCACGTTGAGAAAGCATATCGTCGAGATTCATTCCACCAACGACAGTTCGGATATTGGTCATAACAAGATTTGAAATAGCTGATGCAAGATTATCGACTTCATAAGCTGCCTTCGCTGCATCAATGACCTGGACAAAACAAACAGCATCGATAGTTACGTTTGCATTGTCTTTCGAAATAACTTCCTGTTTAGGAATATCCAGGACGGTTTCCATCATGTTGATCCGTTGACCTATGCGGTCCATAAACGGGATCAGGAAGTGAAGACCGGGGGAAAGCGTATGGGTATATTTACCAAAGCGTTCCACTGTCCAGGCATTACCTTGTGGCACGATTTTGACGGCAGATTTAACGAATATCAGCGCGACTAAAATTAATAAGAGTAACGGAATAGATGTAACTATTGGCGTAATTATAGCGTCAATCATGATTAATCCTCTCCCCATTCATCACAATATGCTTCAACCGGAGTTTTTCCTGCTTCATAATCACCACGCCATGCTTCCGCATCGGCGGCATTTCCACCGCGTAACTCTGCGTAATCCATTAATAGCTCGTGCCATTCTTCAAATGTTACGGTTTGTTTAGTGGAACCAAAATCAGCCATATGTATTTCCTGCATTTTGTGGAGGTATCTCTTTGCAAATGAAATCTGAAATATGAACATCAACAGAAAAACACTTTCCTGCTTCTTTTTCTTTTGAGAAGTTTTTTCTGCATTCTTCAGCGGTCATAGGGAAGTCGCTGTAATGAGTCCATTTCACGGGGCCACCACCTGGTTTTTTGATGGTTGCGTTTATTTGGTACTTAGGCATTCAAAAATACCTCCACTCGATCACCTGTCTTTCTTGAACACATCCAGCGGCGCAGTTCTGTAAAACTATCCTGGATCTCACAACGGGTACTTTTCATTGTGACAGTTGGTATATTGTCGTAGCTGGCATATGCAATCTCTTTTTTGGGTGTGCGCAATATGCCTTTGTCAATATCAAGAATCCTGAACGCGCTAATTCTTCTTTTGTGCTCGTTAAATAGTGTTCGTTTAGTTGCTGTTATCTGCTTCTGTGCTTCCTGGTAAATGTAGGTAAGTACATCATAAGATTGTTGCTGTGTAATAAGGAAATTACGGCTAACATCCGTTGTTGTGAGCGTTTTATTATTTCTTAATCCCCAATAAGCCACGATGATATAGAGTGGATATCCTTTCAAATCTGCCACACATTCAGGAATGACAGGATTAATTTTTAACTTACGCGCCATATTTTCTCCCGGTTGTTTGTCTGAAATAGAAACATCCCCGGAATACAGCGTCATAAGCTAATTATGAAAAGTTGCAGCCACTCACTACAGGCTCTTTATCTGATTGTTCATCATCATGCATAGCTAAACCCCCTTACCTGGAGTGGAGACTGGCAAATCTGGTGTACTTCTGCGTTGCAGCCTGTACGACTTGCTCAGGGAGAGAGTGAGTGAGATTAACTCAACGCGAGTTCACCAAATAAGGGGGTTTAAGTATGCAGAATGATATTTGCGGATAAAGAGTCGTTACCTGTGTAGATGGCGCGGCACGTCACTCTGTTACGTAGCCCGTTGCTGACAGATGCCAGCGGTGCTTGAAATGCACGAATAATAACCTCCTTATGCCAGGGCTTTAGGCAATGACCGCTGTCCGGGGTTGTTAAAGAGCATGAGATGACAAGCGTTCTAACGGGCGTCAAAGAGTGCTCATTTTCCAGGCAATGAGCACAAAAAACGCGATGAACGAGCTTTTTACAATCATCTGGATAGTATTGTGTGTGCGACATTTAAAACCTGTCAATGCCACGAGAGCGATCTAATGATCTGGATAACACCTAAAAAATAGAGTAATATTCTATTTGTTAAATGATGTACGATCTAAAAAT
