# Supplementary material for: Dissemination and Mechanism for the MCR-1 Colistin Resistance
Source: PLoS Pathog. 2016 Nov 28;12(11):e1005957. doi: 10.1371/journal.ppat.1005957 (PMC5125707; doi:10.1371/journal.ppat.1005957)
Supplement: S1 Table — (DOCX) [file ppat.1005957.s003.docx]

**Table S1** Strains and plasmids in this study

| Strains or plasmids | Characteristics | Origins |
| --- | --- | --- |
| Strains |  |  |
| MG1655 | A wild type strain of *E. coli* | Lab stock |
| DH5α | A cloning host of *E. coli* | Lab stock |
| BL21(DE3) | An expression host of *E. coli* for protein production | Lab stock |
| Plasmids |  |  |
| pET28(a) | The T7-driven expression vector, Km^R^ | Novagen |
| pET28*::mcr*-*1* | pET28(a) encoding the *mcr*-*1* gene, Km^R^ | This work |
| pBAD24 | An arabinose-inducible expression vector; Amp^R^ | Lab stock, [[48](#_ENREF_48)] |
| pBAD24::*mcr*-*1* | pBAD24 carrying the wild type version of *mcr*-*1* at the two cuts of EcoRI and SalI; Amp^R^ | Lab stock |
| pBAD24::*lptA-*ng | pBAD24 containing *Neisseria gonorrhoeae lptA* at the two cuts of EcoRI and SalI; Amp^R^ | Lab stock |
| pBAD24::*mcr-1*(ΔTM) | pBAD24 encoding the transmembrane region deletion version of *mcr*-*1*; Amp^R^ | Lab stock |
| pBAD24::*mcr-1*(E246A) | pBAD24 encoding the mutant version of *mcr*-*1* (E246A); Amp^R^ | Lab stock |
| pBAD24::*mcr-1*(T285A) | pBAD24 encoding the mutant version of *mcr*-*1* (T285A); Amp^R^ | Lab stock |
| pBAD24::*mcr-1*(H395A) | pBAD24 encoding the mutant version of *mcr*-*1* H395A); Amp^R^ | Lab stock |
| pBAD24::*mcr-1*(D465A) | pBAD24 encoding the mutant version of *mcr*-*1* (D465A); Amp^R^ | Lab stock |
| pBAD24::*mcr-1*(H466A) | pBAD24 encoding the mutant version of *mcr*-*1* (H466A); Amp^R^ | Lab stock |
