# Supplementary material for: Dissemination and Mechanism for the MCR-1 Colistin Resistance
Source: PLoS Pathog. 2016 Nov 28;12(11):e1005957. doi: 10.1371/journal.ppat.1005957 (PMC5125707; doi:10.1371/journal.ppat.1005957)
Supplement: S2 Table — (DOCX) [file ppat.1005957.s004.docx]

**Table S2** Primers used in this study

| Primers | Primer sequence |
| --- | --- |
| *mcr*-*1*-F(BamHI) | 5'-CG *GGATCC* ATG ATG CAG CAT ACT TCT GTG-3' |
| *mcr*-*1*-R(XhoI) | 5'-CGG *CTCGAG* TCA GCG GAT GAA TGC GGT G-3' |
| *mcr*-*1*-F(XmaI) | 5'-ACTTGA *CCCGGG* ATG ATG CAG CAT ACT TCT GTG-3' |
| *mcr*-*1*-R(XmaI, SGGRGG) | 5'-ACC ACC ACG ACC ACC ACT GCG GAT GAA TGC GGT GCG-3' |
| *gfp*-F(SGGRGG): | 5'-AGT GGT GGT CGT GGT GGT ATG AGT AAA GGA GAA GAA CTT TT-3' |
| *gfp*-R(SphI) | 5'-AGTGCT *GCATGC* TTA TTT GTA TAG TTC ATC CAT GC-3' |
| *mcr-1*-F2(EcoRI, 1-21) | 5'- AACC *GAATTC* ATGATGCAGCATACTTCTGTG-3' |
| *mcr*-*1*-F3(truncated, EcoRI, 541-561) | 5'-AACC *GAATTC* ATG AGT TTC TTT CGC GTG CAT AAG-3’ |
| *mcr-1*-R2(SalI, 1608-1626) | 5'-CCG *GTCGAC* TCA GCG GAT GAA TGC GGT G-3' |
| *lptA*_ng-F (EcoRI) | 5'- AACC *GAATTC* ATG ATA AAA CCG AAC CTG AGG-3’ |
| *lptA*_ng-R (SalI) | 5'-CCG *GTCGAC* TCA GCG CGG ACG GCG GCA G-3’ |
| *mcr-1*(E246A)-F | 5’-TTC GTC GTC GGT GCG ACG GCA CGC GCC-3’ |
| *mcr*-*1*(E246A)-R | 5’-GGC GCG TGC CGT CGC ACC GAC GAC GAA-3’ |
| *mcr*-*1*(T285A)-F | 5’-TGC GGC ACA TCG GCG GCG TAT TCT GTG-3’ |
| *mcr*-*1*(T285A)-R | 5’-CAC AGA ATA CGC CGC CGA TGT GCC GCA-3’ |
| *mcr*-*1*(D465A)-F | 5’-CTG TAT GTC AGC GCG CAT GGC GAA AGT-3’ |
| *mcr*-*1*(D465A)-R | 5’-ACT TTC GCC ATG CGC GCT GAC ATA CAG-3’ |
| *mcr*-*1*(H466A)-F | 5’-TAT GTC AGC GAT GCG GGC GAA AGT CTG-3’ |
| *mcr*-*1*(H466A)-R | 5’-CAG ACT TTC GCC CGC ATC GCT GAC ATA-3’ |
| *mcr*-*1*(H395A)-F | 5’-ACC AAA TGG GCA ATG CGG GGC CTG CGT ATT T-3’ |
| *mcr*-*1*(H395A)-R | 5’-AAA TAC GCA GGC CCC GCA TTG CCC ATT TGG T-3’ |
| ck-4-F | 5’-GAG CGG TAT CTC CTT TCT CA-3’ |
| ck-4-R | 5’-TCA TCA GTG CTC TGG AAC TG-3’ |
| ck-17-F | 5’-CAG GCC AGA GAA GAA CAT CT-3’ |
| ck-17-R | 5’-AGA AGT ATG CTG CAT CAT GAG-3’ |
| 16S-F | 5'-AAA TTG AAG AGT TTG ATC ATG G- |
| 16S-R | 5'-GCT TCT TTA AGG TAA GGA GGT-3' |
| *mcr*-*1*-F1 | 5'-ATG ATG CAG CAT ACT TCT GTG-3' |
| *mcr*-*1*-R1 | 5'-TCA GCG GAT GAA TGC GGT G-3' |
| *nikB*-F | 5'-GAT GAA CTT GAT CAT CGT GTT GT-3' |
| *nikB*-R | 5'-GTA ATT CTG ACG AAA AAG AGG A-3' |
| *pilP*-F | 5'-TTA AAG AAT AAG CTG GCG TTT C-3' |
| *pilP*-R | 5'-ATG TTA AAA ATA ATT AAA CCA ACG-3' |
| *virD4*-F | 5'-AAT GTC AAC ATG ATT GTT AC-3' |
| *virD4*-R | 5'-GAA CAT AAC CCG GAC CTG AAA T-3' |
| *virB4*-F | 5'-AAC TCT TTT TCA GTA AGC CCA AT-3' |
| *virB4*-R | 5'-TTA ATG TTT GTT GTG GAT TAC AAC C-3' |
| *tnpA*-F | 5'-GGT TTT CGG GCT TTT TAA GAG-3' |
| *tnpA*-R | 5'-TAG CAC ATA GCG ATA CGA TG-3' |
| *hp*-F | 5'-GAT AAG CAA ACT GGC ATC ACG-3' |
| *hp*-R | 5'-GAA CCC TGT ATA TAG CCT GTC-3' |
| F1 (2429-2447) | 5’-GCT AGT GTG CTT CTT GCG T-3’ |
| R1 (2891-2910) | 5’-GCG TAA AGG GCG GTA AAA GT-3’ |
| F2 (5073-5093) | 5’-GAA ACC TCG GAT ATA CAG TGT-3’ |
| R2 (5631-5650) | 5’-CCT GTC CGT TTG GAC TTA AG-3’ |
| F3 (7802-7821) | 5’-GAG TTT CCC CAT TCC GTT TC-3’ |
| R3 (8469-8489, 10660-10680) | 5’-GGC CAT GAT CAT GCT GAT TTG-3’ |
| F4 (8469-8489, 10660-10680) | 5’- CAA ATC AGC ATG ATC ATG GCC-3’ |
| R4 (8979-8999, 11170-11190) | 5’-GGC TCA CCA CAG AAA TCA TTG-3’ |

The restriction sites are underlined and bold italic letters.
