# Supplementary figures and images for: Dissemination and Mechanism for the MCR-1 Colistin Resistance
Source: PLoS Pathog. 2016 Nov 28;12(11):e1005957. doi: 10.1371/journal.ppat.1005957 (PMC5125707; doi:10.1371/journal.ppat.1005957)

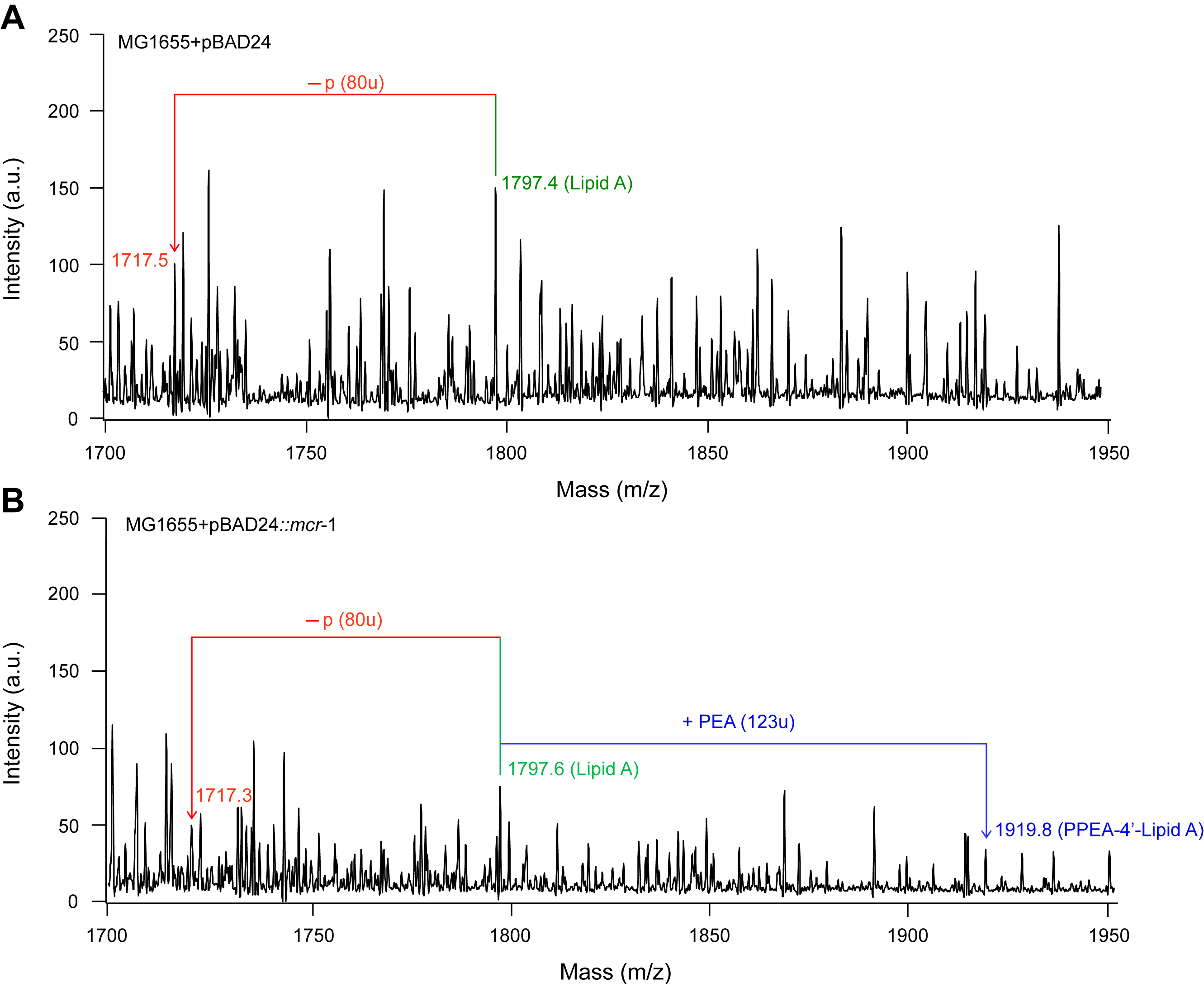

Supplement: S1 Fig — A. ESI-MS-based analyses for the lipid A profile of LPS extracted from the negative control E. coli MG1655 strain carrying empty vector pBAD24 B. ESI-MS determination of the LPS Lipid A components from the E. coli MG1655 strain with the plasmid pBAD24::mcr-1 Bis-phosphorylated hexa-acylated lipid A (m/z = ~1797) and the mono-phosphorylated derivative (m/z = ~1717) were detected in the two E. coli strains. The MG1655 strain with the expression of the mcr-1 gene was consistent with one PEA added to the bis-phosphorylated structure (m/z = 1920; i.e., 1797 + 123). (TIF) [file ppat.1005957.s005.tif]

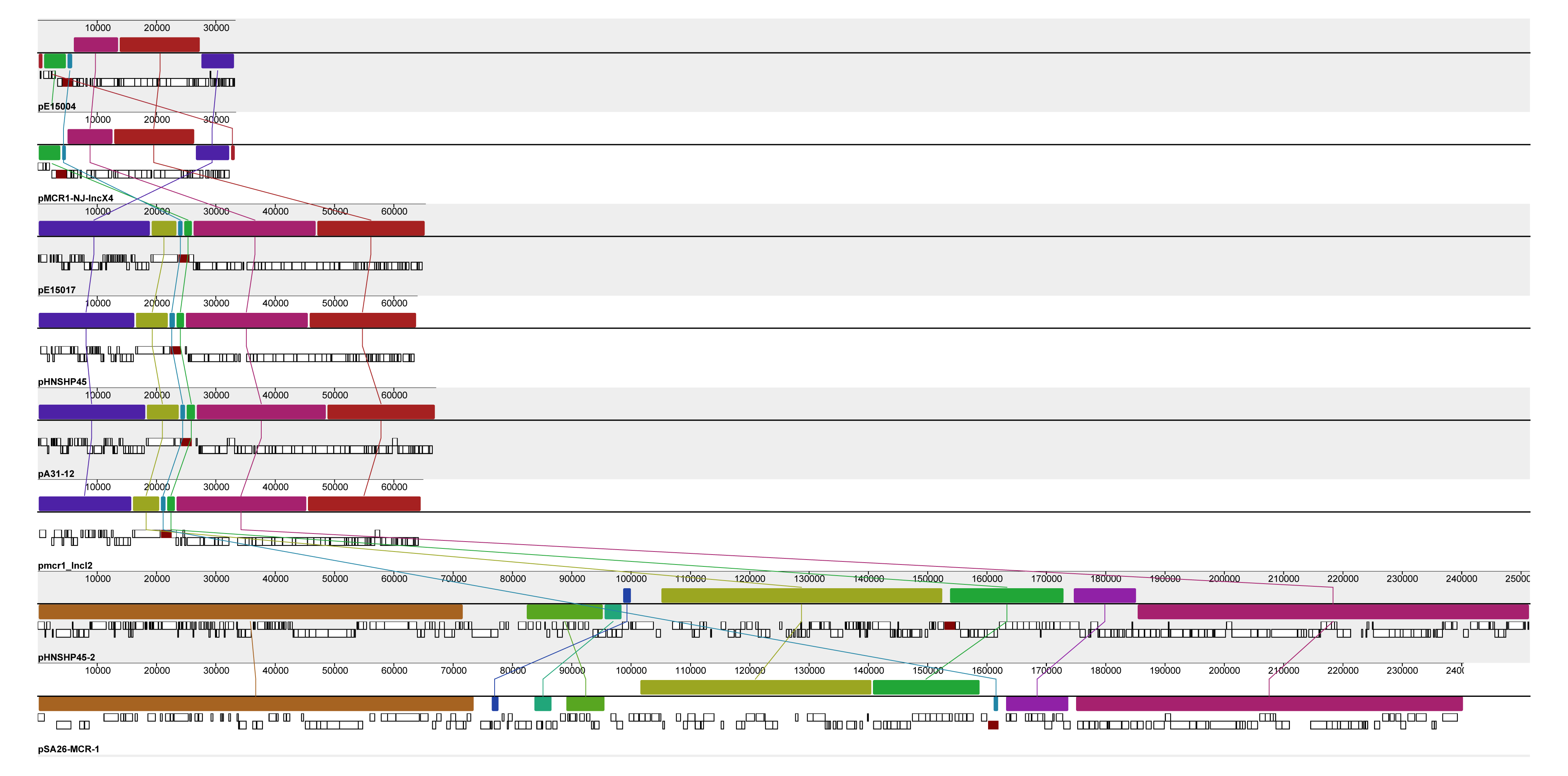

Supplement: S2 Fig — The comparative genomic analysis was performed using Mauve alignment software [49]. The alignment was shown using Mauve’s locally collinear blocks (LCBs). Each LCB is a homologous region that was shared by two or more plasmids. The comparative was viewed using the solid LCB coloring style, that is, solid color was drawn for each LCB. Open Reading Frames (ORFs) were displayed as blank rectangles below each plasmid sequence, and the solid red rectangles indicate the mcr-1 gene. (TIF) [file ppat.1005957.s006.tif]

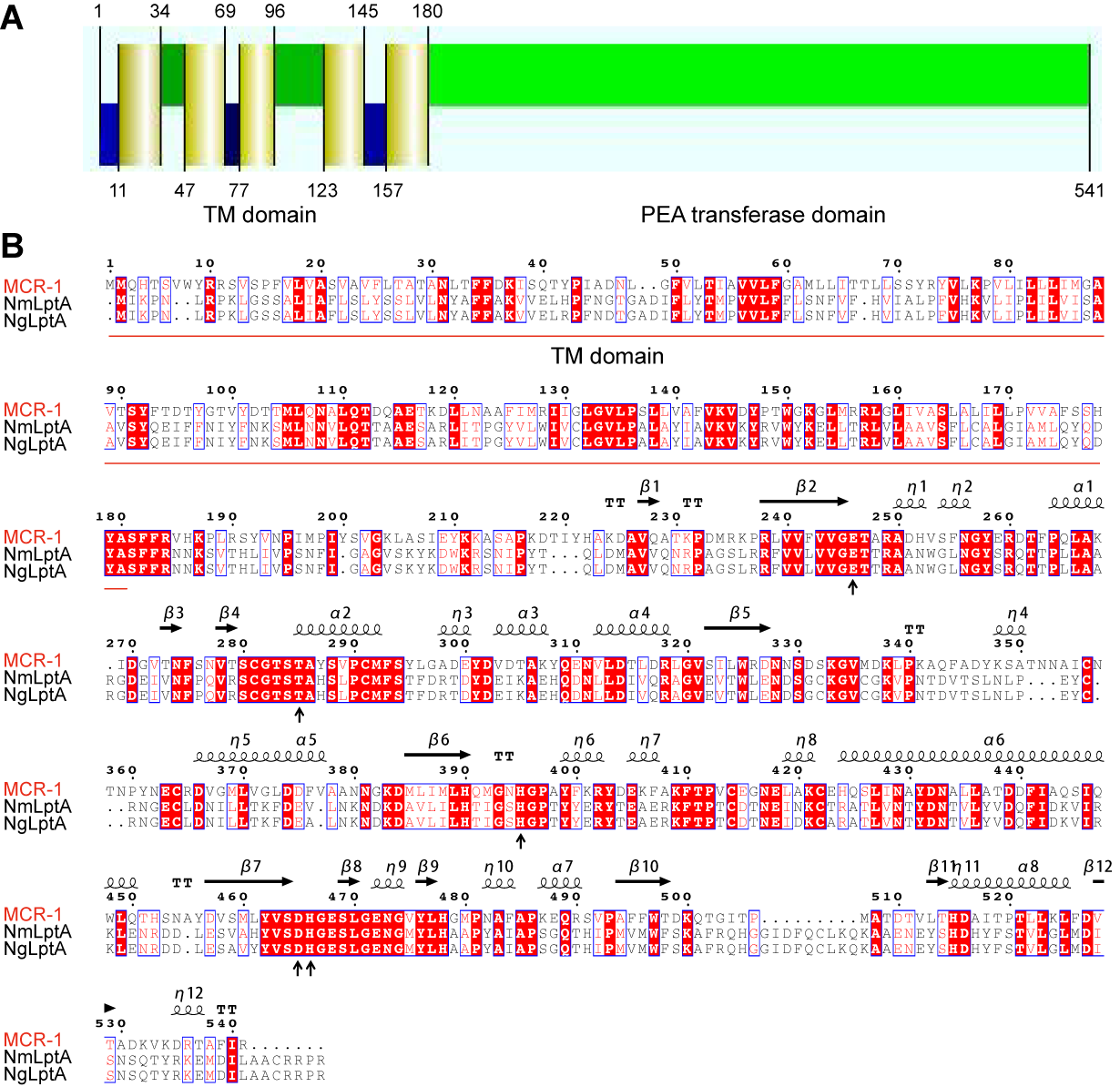

Supplement: S3 Fig — A. Transmembrane prediction for the MCR-1 protein B. Multiple sequence alignments of the MCR-1 protein with the Neisseria LptA protein The topological structure of the MCR-1 protein was predicted with Philius Transmembrane Prediction Server (http://www.yeastrc.org/philius/pages/philius/runPhilius.jsp). The alignment of protein sequences was conducted using Clustal Omega (http://www.ebi.ac.uk/Tools/msa/clustalo/), and the output was given following the process by the program ESPript 2.2 (http://espript.ibcp.fr/ESPript/cgi-bin/ESPript.cgi) [48]. Identical residues are in white letters with red background, similar residues are in the form with mixture of red/black letters, and the varied residues are in black letters. The important residues critical for Zn2+ binding and/or substrate binding are highlighted with arrows. Abbreviations: TM, Tran-smembrane; PEA, Phosphoethanolamine; Nm, Neisseria meningitidis; Ng, Neisseria gonorrhoeae, LptA, Lipid A PEA transferase (TIF) [file ppat.1005957.s007.tif]

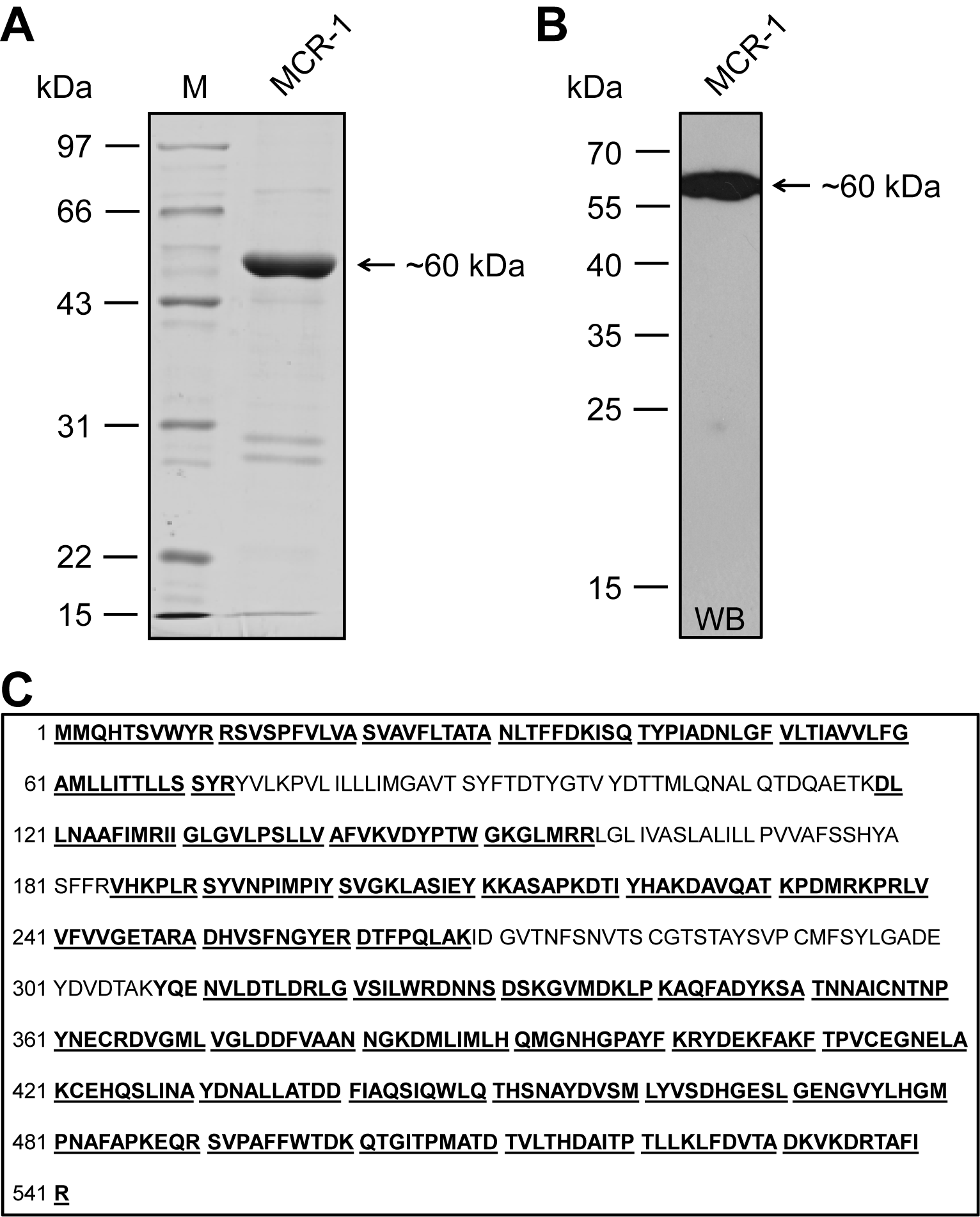

Supplement: S4 Fig — A. SDS-PAGE profile for the purified transmembrane protein MCR-1 B. Western blot analyses for the purified MCR-1 protein with the anti-6XHis tag primary antibody Designations: M, marker; kDa, kilo-dalton; WB, western blot. C. MS verification of the recombinant MCR-1 protein The tryptic peptides with 79.5% coverage to the MCR-1 sequence are given in bold and underlined type. (TIF) [file ppat.1005957.s008.tif]
